# Accelerated synthesis of immunomodulatory imide drugs and their derivatives via continuous flow chemistry

**DOI:** 10.1038/s42004-026-01956-1

**Published:** 2026-03-03

**Authors:** Tianyi Hou, Junrong Huang, Yongheng Li, Yuxiang Zhu, Hengzhi You, Fen-Er Chen

**Affiliations:** 1 School of Biomedical Engineering, Harbin Institute of Technology (Shenzhen), Shenzhen, China; 2 Green Pharmaceutical Engineering Research Center, Harbin Institute of Technology (Shenzhen), Shenzhen, China; 3 Engineering Center of Catalysis and Synthesis for Chiral Molecules, Department of Chemistry, Fudan University, Shanghai, China

**Keywords:** Chemical engineering, Diversity-oriented synthesis, Flow chemistry

## Abstract

Immunomodulatory imide drugs are widely used in the treatment of multiple myeloma, yet existing production routes are often lengthy and inefficient, underscoring the need for more efficient and broadly applicable synthetic strategies. Herein, we report the synthesis of lenalidomide (63% overall yield) and pomalidomide (62% overall yield) using an integrated continuous flow platform with residence times of 42 minutes and 52 minutes, respectively. Additionally, both continuous flow processes are readily conducted on gram scale. Compared with known methods, our approach improves solvent compatibility across the reaction sequence and enables uninterrupted continuous flow synthesis of both drugs. The products are obtained without the need for column chromatography, and both immunomodulatory imide drugs (IMiDs) can be accessed from a common intermediate through our developed route. Furthermore, we explore the synthesis of CRBN ligand-linkers under continuous flow, affording a series of derivatives with diverse properties in yields exceeding 90%.

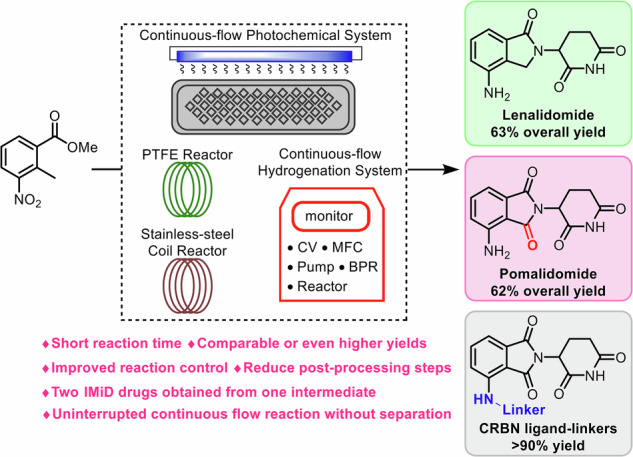

## Abstract (web summary)

Immunomodulatory imide drugs, including lenalidomide, are crucial to the treatment of multiple myeloma, yet existing manufacturing routes are inefficient and time-consuming. Here, the authors present an integrated continuous-flow synthesis platform with improved solvent compatibility and process efficiency for the production of lenalidomide, pomalidomide and their derivatives.

## Introduction

**Fig. 1:**
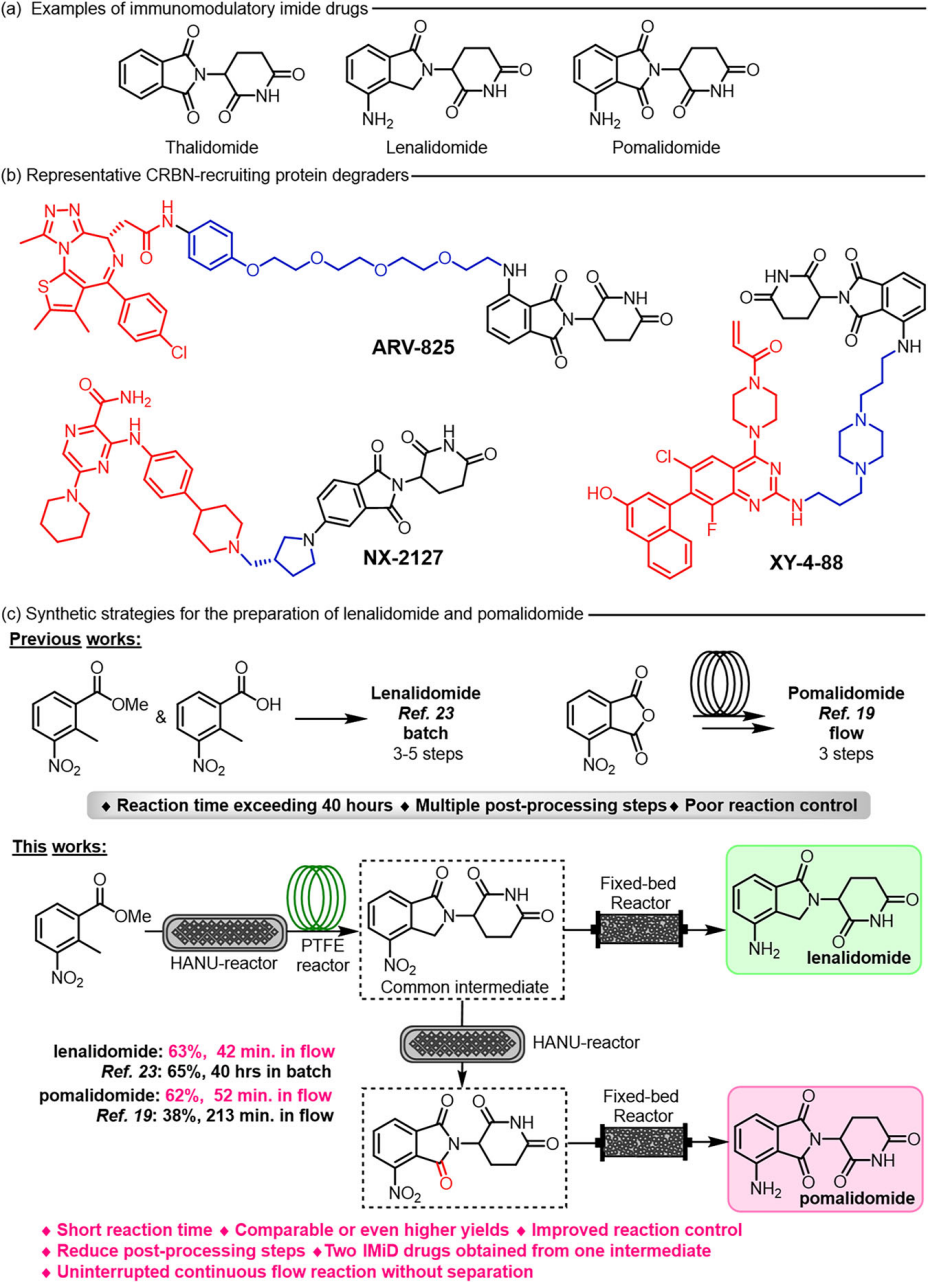
Immunomodulatory imide drugs. **a** Examples of immunomodulatory imide drugs; **b** Representative CRBN-recruiting protein degraders; **c** Synthetic strategies for the preparation of lenalidomide and pomalidomide.

The discovery of immunomodulatory imide drugs (IMiDs) traces back to the 1950s, when the pharmaceutical company Grünenthal developed thalidomide as a treatment for insomnia^1^. Decades later, its anti-inflammatory, anti-angiogenic, and anti-myeloma activities were elucidated, leading to the emergence of the IMiDs class, which includes lenalidomide and pomalidomide (Fig. 1a)^2–4^. In 2010, Ito et al. discovered that IMiDs bind to cereblon (CRBN), thereby enabling their use as CRBN-recruiting ligands in proteolysis-targeting chimeras (PROTACs), a class of heterobifunctional protein degraders (Fig. 1b)^5–12^. Today, IMiDs rank among the most widely employed CRBN ligand–linkers in PROTACs design^13,14^. Driven by therapeutic applications and substantial market demand, numerous synthetic routes to lenalidomide and pomalidomide have been developed^15–20^. Most approaches converge on catalytic hydrogenation of the nitro group in the key intermediates 3-(4-nitro-1-oxoisoindolin-2-yl)piperidine-2,6-dione and 2-(2,6-dioxopiperidin-3-yl)-4-nitroisoindoline-1,3-dione to furnish the final product. Despite the structural similarity of the two precursors, the reported syntheses proceed through markedly different pathways. The majority rely on three or more batch steps, with the longest sequences comprising up to five steps and requiring more than 40 h^18,21–23^. These routes typically involve extended reaction times and laborious post-reaction workups (Fig. 1c). In 2022, Maria Ivanova et al. reported a stepwise continuous flow synthesis of pomalidomide starting from Boc-L-glutamine, delivering an overall yield of 38%^19^. However, the individual steps were not telescoped in series, and to date, no fully integrated continuous flow synthesis of IMiDs has been reported.

In addition, in the context of designing CRBN ligand–linkers for PROTACs, rapid access to CRBN ligand–linkers remains critical for the exploration and optimization of new PROTACs, particularly at the preclinical stage, despite advances in computational tools for degrader modeling and evaluation^24–28^. Pomalidomide derivatives constitute one class of CRBN ligand–linkers, their synthesis typically relying on alkylation of aryl amines, which are often weak nucleophiles and display poor chemoselectivity^29^. Acylation provides convenient access to pomalidomide derivatives; however, incorporation of an additional amide linkage increases polar surface area and introduces additional hydrogen-bond acceptors, features that can be detrimental for certain protein degraders^30^. In contrast to the aforementioned methods, nucleophilic aromatic substitution (S_N_Ar) of 4-fluorothalidomide provides pomalidomide conjugates in a highly selective manner. Currently, variations in solvent choice^31^ and the use of elevated temperatures^32^ have been explored to enhance the synthetic efficiency of this S_N_Ar transformation. However, despite these improvements, continuous flow technology, which offers unique advantages for enhancing synthetic efficiency and industrial scalability, has not yet been applied to the synthesis of pomalidomide derivatives.

During the last two decades, flow chemistry has emerged as a powerful platform for improving the efficiency of complex multistep syntheses, owing to its superior mixing, rapid heat and mass transfer, enhanced safety, simplified workup, reduced waste and energy consumption^33–43^. Encouraged by these advances, we have developed a fully continuous flow synthesis of lenalidomide that overcomes the solvent compatibility issues in the multistep process. By implementing an integrated continuous flow system, we successfully achieved the continuous synthesis of lenalidomide and a series of structural analogues. Recognizing the structural similarity between two intermediates, we designed a synthetic route to access a pomalidomide precursor, thereby enabling the preparation of both IMiDs from a single common intermediate. Furthermore, we have employed continuous flow methods to enhance the synthesis efficiency of CRBN ligand–linkers and to enable the preparation of several PROTACs of interest. Collectively, these advancements are expected to enhance the overall efficiency of IMiDs and PROTACs synthesis, providing a basis for further industrial applications.

## Results and discussion

### A three-step continuous flow total synthesis of lenalidomide

Details of the continuous flow system we constructed are provided in Supplementary Note 1. Our three-step continuous flow synthesis of lenalidomide (**5a**) began with the bromination of commercially available methyl 2-methyl-3-nitrobenzoate (**1**) using 1-bromopyrrolidine-2,5-dione (NBS) to afford methyl 2-bromomethyl-3-nitrobenzoate (**2**) under flow in a Wohl-Ziegler bromination reactions shown in Supplementary Table 1. Conventional thermal conditions using radical initiators such as azobisisobutyronitrile (AIBN) and benzoyl peroxide (BPO) present explosion hazards, whereas photobromination avoids these reagents and associated risks^44–48^. In addition, the flow photochemical setup offers superior light penetration, precise control over residence time and temperature, and improved energy efficiency^49–51^. Since MeOAc, the solvent commonly used in batch photobromination, afforded only a 51% yield (Supplementary Table 1, Entry 1), we subsequently optimized the solvent and identified acetonitrile as the optimal medium (Supplementary Table 1, Entries 2-4). Using acetonitrile as the reaction solvent, we found that the conversion rate increased further as the light intensity decreased. A 10 min residence time (*t*_R_) at a light intensity of 403.2 W resulted in complete conversion of **1** (Supplementary Table 1, Entry 12).

In the second step of the cyclization reaction (Supplementary Table 2), the base plays a key role in this reaction, facilitating the deprotonation of the ammonium salt and acceleration of the cyclization step. We first screened various organic and inorganic bases and identified DIPEA as the optimal base, affording the product in 64% yield (Supplementary Table 2, Entry 7). ^1^H NMR analysis of the reaction mixture discovered peaks corresponding to the uncyclized intermediate. However, extending the residence time failed to improve the yield. Similarly, neither increasing the equivalents of base nor raising the temperature improved the yield (Supplementary Table 2, Entries 8–10).

This troubling issue prompted us to consider the cause of the incomplete cyclization, and we speculated that DIPEA may not have fully performed its intended role after mixing. We observed that a DMF solution of **3** and DIPEA gradually darkened upon heating, which could be attributed to the slow deprotonation of **3**. To investigate this effect, we performed a control experiments (Supplementary Table 2, Entries 11–12). Specifically, **3** was preheated with DIPEA in DMF at 50 °C for 10 min until a blue–purple coloration developed, and the resulting mixture was then introduced into reactor II at 80 °C with a residence time of 30 min, successfully affording the desired product in an increased yield of 83% (Supplementary Table 2, Entry 11).

In the third step of the synthesis, the batch reduction of lenalidomide precursor **4** is typically carried out using water or methanol as the solvent, which leads to a paste-like reaction mixture due to the low solubility of **4**^17,22,23^. Such a mixture is likely to cause blockages in the piping and packed bed, and to avoid a solvent switch and potential compatibility issues with the solvent used in the previous step, we selected DMF as the reaction solvent, which also proved suitable for the hydrogenation step in our flow setup. Initial screening of the commercially available catalysts identified 5% Pd/Al_2_O_3_ as the optimal catalyst (Supplementary Table 3, Entries 1–4). Further optimization of the flow rate established the optimal conditions as 5% Pd/Al_2_O_3_ at 100 °C, back pressure 0.5 MPa, and *t*_R_ = 2 min, leading to the production of lenalidomide **5a** in 98% yield (Supplementary Table 3, Entry 6).

**Fig. 2:**
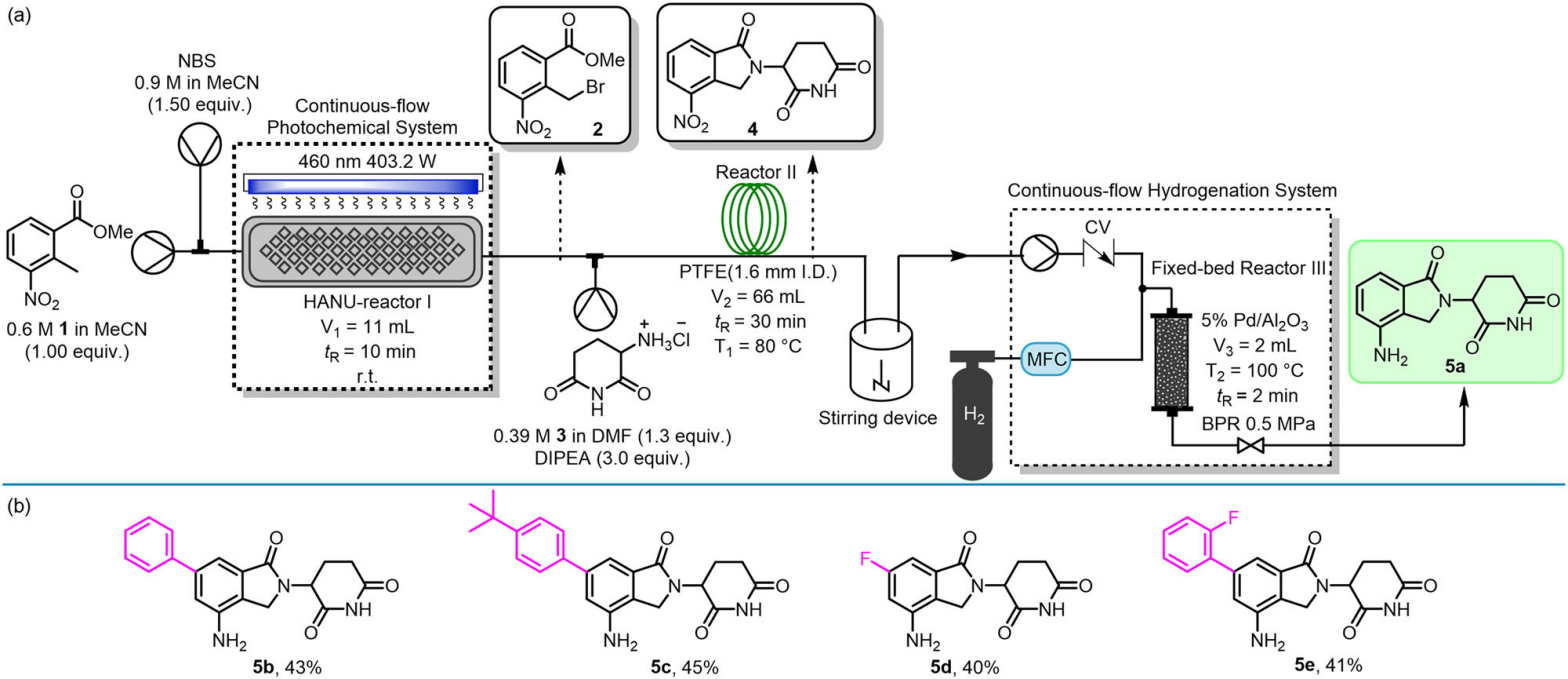
Continuous flow total synthesis of lenalidomide 5a and its derivatives 5b–5e. **a** Schematic diagram for the continuous flow synthesis of lenalidomide **5a**; **b** Synthesis of lenalidomide derivatives **5b**–**5e**.

After successful stepwise optimization, we eventually developed a three-step continuous flow total synthesis of lenalidomide **5a** from commercially available starting materials, as summarized in Fig. 2; a photograph of the experimental setup is provided in Supplementary Fig. 1. The synthesis of lenalidomide **5a** started with the photo-bromination of **1** (0.6 M, 1.0 equiv.) with NBS (0.9 M, 1.5 equiv.) using MeCN as the solvent. The photochemical flow system comprised HANU Reactor I (11 mL work volume), an LED light source equipped with a recirculating chiller, and associated optics. The stream was irradiated at 460 nm at room temperature (lamp power 403.2 W) with a residence time of 10 min. Prior to mixing, a DMF solution of **3** (0.39 M, 1.3 equiv.) and DIPEA (0.90 M, 3.0 equiv.) was preheated at 50 °C for 10 min to develop a blue–purple coloration. This preactivated solution was then merged via a T-mixer with the reaction stream from the first step (bromide **2**), and the combined stream was directly delivered to PolyTetraFluroEthylene (PTFE) Reactor II (1.6 mm i.d., 66 mL work volume) at 80 °C with a residence time of 30 min. Subsequently, the output solution **4** from Reactor II was treated with H_2_ (20 sccm) and passed through a 5% Pd/Al_2_O_3_ packed-bed reactor (2 mL work volume) at 100 °C with a residence time of 2 min and a back pressure of 0.5 MPa.

Finally, lenalidomide (**5a**) was obtained by evaporation of the collected reaction mixture, followed by precipitation upon addition of water. Compared with the traditional three-step batch synthesis, which requires over 40 h, our continuous flow synthesis eliminated intermediate work-up and isolation steps, and achieved completion of the sequence within a total residence time of 42 min, delivering lenalidomide in an overall yield of 63%. Notably, no column chromatography was required for purification in the final step; simple crystallization afforded the pure product. To evaluate the scalability of our flow system, we conducted a gram-scale synthesis of **5a**. After 2 h of continuous operation, 1.03 g of **5a** was obtained, corresponding to a productivity of 0.515 g/h. Moreover, the versatility of this assembled continuous flow system was exemplified by the successful synthesis of a series of lenalidomide derivatives **5b**–**5e** with the same procedure, leading to the overall yields ranging from 40% to 45% (please see SI for details).

### Two-step continuous flow synthesis of pomalidomide

Given the close structural similarity between lenalidomide and pomalidomide, we next sought to extend our strategy to pomalidomide. Upon completion of the continuous flow synthesis of lenalidomide, we therefore designed a synthetic route that relies on the C(sp^3^)-H oxidation of **4** to install an additional carbonyl group, thereby furnishing the pomalidomide precursor **6** via a pathway distinct from previously reported methods. Initial screening of the benzylic C(sp^3^)–H oxidation protocols revealed that SeO_2_, I_2_, and PhI(OAc)_2_ all failed to convert **4** to **6**, leaving the starting material essentially unchanged (Table 1, Entries 1–3). Using H_2_O_2_ as the oxidant afforded merely 5% conversion (Table 1, Entry 4). A previously reported method for synthesizing thalidomide derivatives described a benzylic oxidation side reaction^52^. Inspired by this report, we applied the same conditions to 4, but observed only consumption of the starting material without formation of **6** (Table 1, Entry 5). Pleasingly, adding water as a nucleophilic reagent along with a large excess of NBS successfully promoted the oxidation, leading to an 82% conversion (Table 1, Entry 6). A plausible mechanism involves over-bromination of **4** to the corresponding unstable dibromide **4a**, followed by the nucleophilic substitution by H_2_O to afford **4b**, and final dehydration furnished the desired product **6**. Encouraged by this outcome, we next screened a series of photoinduced conditions and identified NBS in MeCN:H_2_O (1:0.1) with 460 nm (20 W) irradiation at room temperature as optimal, which delivered **6** in 97% yield (Table1, Entry 7). This photoinduced strategy was proven to be more efficient than the thermal initiation strategy, requiring only 1 h to achieve completion.

**Table 1:** Optimization table for the oxidation of 4 and an elucidation of its plausible mechanism


Entry	Oxidants (equiv.)	Additives (equiv.)	Solvent	Conditions	Yield (%)^a^
1	SeO_2_ (1.3)	Pyridine (1.3)	DMF	rt for 5 h, then 80 °C for 15 min	–
2	I_2_ (0.04)	TsOH (1.0)	DMSO	130 °C for 16 h	–
3^b^	PhI(OAc)_2_ (2.0)	–	DMSO	120 °C for 30 min	–
4	H_2_O_2_ (5.0)	HBr (0.9)	CH_2_Cl_2_	r.t. for 12 h	5
5	NBS (2.0)	AIBN (0.1)	MeCN	80 °C for 9 h	–
6	NBS (4.0)	AIBN (0.1)	MeCN:H_2_O = 1:0.1	80 °C for 9 h	82
7	NBS (4.0)		MeCN:H_2_O = 1:0.1	460 nm LEDs for 1 h	97
8	NBS (4.0)		CCl_4_:H_2_O = 1:0.1	460 nm LEDs for 1 h	–
9	NBS (4.0)		MeCN:CCl_4_:H_2_O = 1:1:0.1	460 nm LEDs for 1 h	97

The table summarizes the optimization of the oxidation reaction conditions for converting compound **4**–**6**, with entry 7 achieving the highest yield of 97% using NBS as the oxidant under 460 nm LED irradiation for 1 h.

Reaction conditions: all reactions were performed using 144.62 mg of **4** (0.5 mmol, 1.0 equiv.) in the specified solvent (0.2 M).

^a^Isolated yield.

^b^Reaction was performed under 100 W microwave irradiation.

However, translating this photoinduced strategy to continuous flow proved challenging because poor solubility of **4** in MeCN led to the precipitation within the tubing. Our most straightforward approach was therefore to identify a solvent system that would ensure a homogeneous feed and suppress precipitation under continuous flow conditions. Disappointingly, none of the commonly used solvents dissolved **4** to an appreciable extent (Supplementary Table 4). Interestingly, we noticed that **4** formed a stable suspension in carbon tetrachloride (CCl_4_), suggesting a non-settling slurry feed could be used under continuous flow operation. However, using CCl_4_:H_2_O (1:0.1) as the solvent under otherwise identical conditions failed to furnish **6** (Table 1, Entry 8). By contrast, employing a MeCN:CCl_4_:H_2_O (1:1:0.1) mixed solvent afforded **6** in 97% yield (Table1, Entry 9).

On the basis of our solubility studies of lenalidomide precursor **4**, the reaction solvent for this flow step was revised, thereby resolving the in-line settling of **4**; optimization details are provided in Supplementary Table 5. Using a mixed solvent of MeCN:CCl_4_:H_2_O (1:1:0.1), solutions of **4** (0.2 M) and NBS (0.80 M) were stirred for 5 min and then fed into the continuous flow photochemical system, which was irradiated at 460 nm (576 W) at room temperature with a 10 min residence time, affording **6** in 98% yield (Supplementary Table 5, Entry 5).

**Fig. 3:**
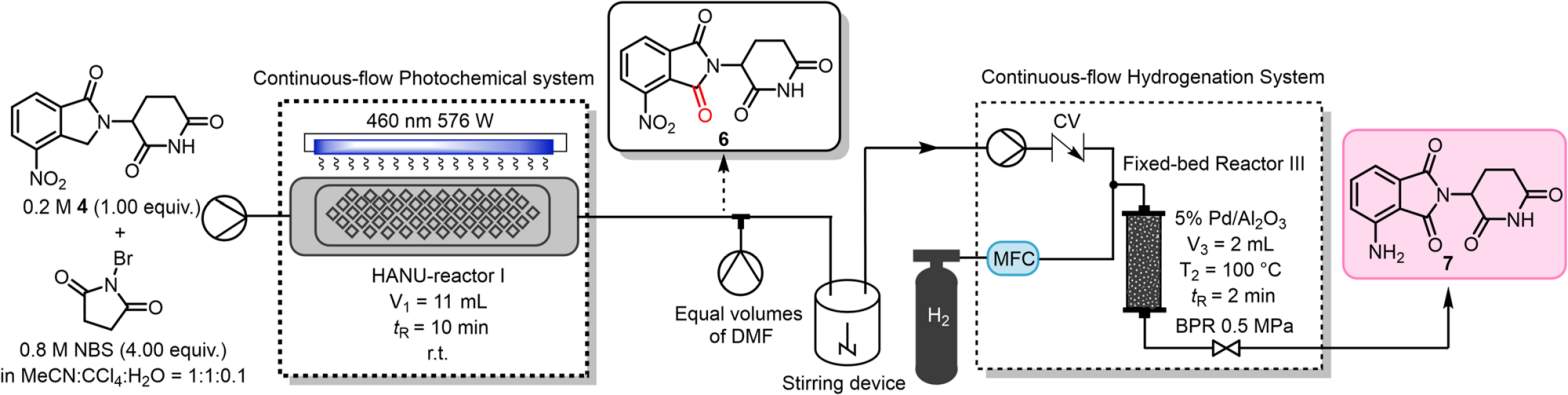
Schematic diagram for the continuous flow synthesis of pomalidomide. Synthesis of pomalidomide **7** from common intermediate **4** via a two-step continuous flow process.

Below we outline the two-step continuous flow sequence that furnishes pomalidomide **7**, as summarized in Fig. 3; a photograph of the experimental setup is provided in Supplementary Fig. 2. First, using a combination of MeCN:CCl_4_:H_2_O (1:1:0.1) as the solvent, solutions of **4** (0.20 M) and NBS (0.80 M) were combined, stirred for 5 min, and fed into the continuous flow photochemical system. The reactor was irradiated at 460 nm (576 W) at room temperature with a residence time of 10 min. We attempted but failed to feed this stream directly into the hydrogenation reduction system using the previously optimum conditions, which is attributed to a gas leak occurring through the packed bed. We suspect that at 100 °C, partial vaporization of the mixed solvent occurred despite the applied backpressure, leading to product precipitation and blockage of the packed bed. To suppress solvent boiling and improve solubility, we therefore introduced an additional DMF stream as a high-boiling and better solubilizing co-solvent. After mixing the two streams evenly through a T-mixer, the mixture was placed in a stirring device before being further introduced into the continuous hydrogenation system. The resulting solution was passed through a packed-bed hydrogenation reactor charged with 5% Pd/Al_2_O_3_ (2 mL work volume) at 100 °C (*t*_R_ = 2 min; back-pressure 0.5 MPa).

The collected reaction mixture was evaporated, and the crude mixture was then precipitated by addition of water to afford the pomalidomide (**7**). Compared with the previously reported batch synthesis and stepwise continuous flow synthesis, we have developed an approach involving the photo-induced oxidation of common intermediate **4** followed by hydrogenation. This two-step continuous flow process achieved a yield of 97% with a residence time of only 12 min, and an overall yield of 62% based on the starting material with a total residence time of 52 min. Furthermore, we evaluated its scalability by conducting a gram-scale synthesis. After 2 h of continuous operation, pomalidomide **7** was obtained in 1.206 g, corresponding to a productivity of 0.603 g/h, which is approximately tenfold higher than that of the previously reported method^19^.

### Continuous flow synthesis of pomalidomide derivatives and PROTACs

Upon completing the continuous flow synthesis of pomalidomide, we turned our attention to the continuous flow preparation of CRBN ligand-linkers. The optimization of reaction conditions was investigated using **8** and propargylamine as model substrates (Experimental procedures for the synthesis of compound **8** and CRBN ligand-linkers can be found in Supplementary Figs. 3, 4. Following literature-reported batch conditions^31^, a DMSO solution of **8** was first merged into a T-mixer with a DMSO solution of propargylamine and DIPEA. The combined stream was fed into a stainless-steel coil reactor (0.8 mm i.d., 2 mL work volume) at 130 °C with a residence time of 30 min, resulting in 41% conversion. Screening of the reaction temperature ranging from 90 to 190 °C revealed that the conversion increased with temperature, reaching a maximum of 63% at both 170 or 190 °C (Table 2, Entries 1–6). Increasing the residence time to 90 min was proven beneficial, with the conversion reaching 85% (Table 2, Entry 8), whereas further extending *t*_R_ to 120 min led to a slight decline in conversion (82%, Table 2, Entry 9). Finally, increasing the loading of propargylamine to 1.5 equiv. improved the conversion to 94% (Table 2, Entry 10), while a further increase to 2.0 equiv. did not provide any additional benefit (93%, Table 2, Entry 11).

**Table 2:** Optimization table for the synthesis of CRBN ligand-linkers


Entry^a^	Amine (equiv.)	Temp. (°C)	*t*_R_ (min)	Conv. (%)^b^
1	1.1	90	30	16
2	1.1	110	30	35
3	1.1	130	30	41
4	1.1	150	30	53
5	1.1	170	30	63
6	1.1	190	30	63
7	1.1	170	60	74
8	1.1	170	90	85
9	1.1	170	120	82
10	1.5	170	90	94
11	2.0	170	90	93

The figure illustrates a continuous flow setup for the amination reaction and presents the optimization results of temperature and amine equivalent, with entry 10 achieving the highest conversion of 94% at 170 °C.

^a^Reaction conditions: all reactions were performed with 165.73 mg of **8** (0.6 mmol, 0.2 M in DMSO) and DIPEA (3.0 equiv.) using the flow setup shown in Table 2.

^b^Conversion was determined by ^1^H NMR analysis of the crude mixture using 1,3,5-trimethoxybenzene as the internal standard.

**Fig. 4:**
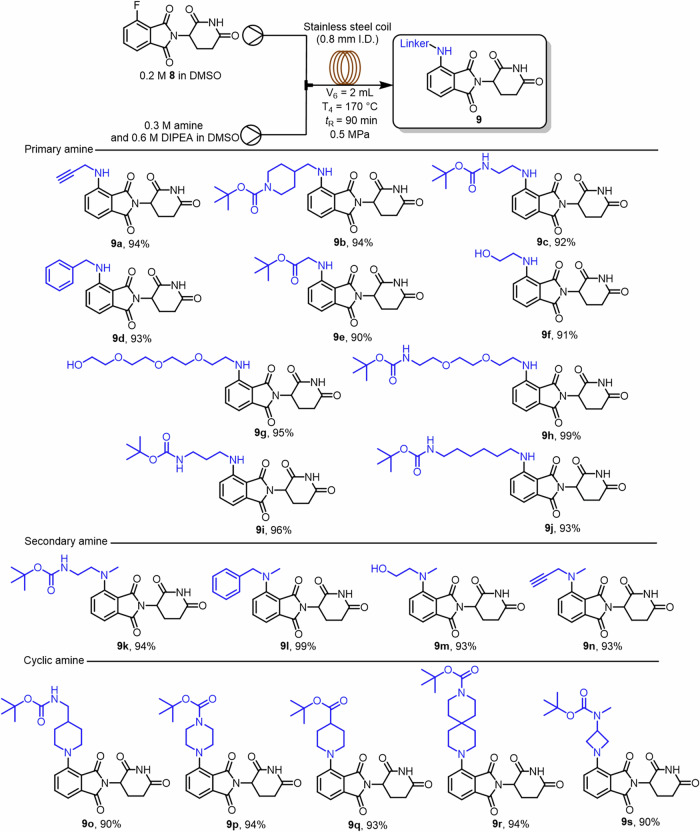
Substrate scope for the S_N_Ar reaction of various amine with 8 under flow. This figure illustrates a diverse library of amine derivatives synthesized under continuous flow conditions.

With the optimum conditions in hand, we sought to explore the amine-nucleophile scope under continuous flow conditions (Fig. 4). Solutions of diverse amines (0.30 M, 1.5 equiv.) and DIPEA (0.60 M, 3.0 equiv.) in DMSO were combined via a T-mixer with a DMSO solution of **8** (0.20 M). The resulting stream was subsequently fed into a stainless-steel coil reactor at 170 °C with a residence time of 90 min under 0.5 MPa back-pressure, furnishing a series of CRBN ligand–linkers in yields above 90%. The prepared compounds include four mono-Boc-protected primary amines. After Boc deprotection with trifluoroacetic acid (TFA), the resulting amines were coupled to the protein of interest (POI) ligand via amide-bond formation. Compounds **9f** and **9g** bear terminal hydroxyl groups that can be selectively elaborated to furnish PROTACs. Secondary and cyclic amines reacted with **8** under the same continuous flow conditions used for the primary amines. Incorporation of cyclic amines imparts greater rigidity to the CRBN ligand–linker and may benefit ternary-complex formation and degradation performance for certain PROTACs. Compared to synthesis under batch conditions, our method offers greater scalability and only requires a residence time of 90 min to obtain the desired compounds, with yields exceeding 90% for all obtained compounds.

**Fig. 5:**
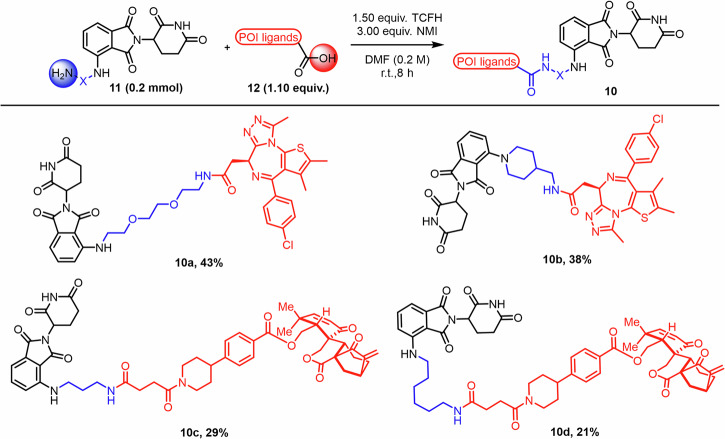
Synthesis of PROTAC degraders. This figure shows the synthesis of PROTAC degraders via amide coupling reaction mediated by TCFH and NMI in DMF.

We coupled the CRBN ligand–linkers generated in continuous flow with a set of POI ligands of interest (Fig. 5). First, we synthesized JQ1-based PROTACs targeting BET (bromodomain and extra-terminal) proteins^53^. In this study, we primarily used JQ1-acid as the small molecule binder, and **9h** and **9o** as linkers to generate **10a** and **10b**. After Boc deprotection of **9h** and **9o**, the reaction mixture was concentrated and directly subjected to TCFH/NMI-mediated peptide coupling without column chromatography, affording the corresponding conjugates in 43% and 38% yield, respectively. Additionally, compounds **10c** and **10d** were synthesized by conjugation with the natural product Laxiflorin B, a known inhibitor of ERK1/2^54^. The Boc protecting groups of intermediates **9i** and **9j** were first removed, followed by removal of excess solvent under reduced pressure. Subsequently, the resulting products reacted with maleic anhydride and then coupled with Laxiflorin B, which had been pre-functionalized with a linker, affording **10c** and **10d** in 29% and 21% yield, respectively. Further investigation of their biological activity is in progress.

## Conclusions

In conclusion, we present an efficient integrated continuous flow synthesis of lenalidomide and its derivatives that proceeds without intermediate isolation or purification. In addition, we developed a concise synthetic route for pomalidomide via a common intermediate. By addressing key challenges, including solvent incompatibility and compound precipitation issues, we successfully achieved the integrated continuous flow synthesis of lenalidomide (63% overall yield) and pomalidomide (62% overall yield), with a remarkably residence times of 42 min and 52 min, respectively. Both continuous-flow processes can be scaled to the gram level. The derivatives of lenalidomide were synthesized using the integrated continuous flow system, with yields ranging from 40% to 45%. Furthermore, we optimized the synthesis of CRBN ligand–linkers using continuous flow, enabling the production of a series of CRBN ligand–linkers with different properties in yields above 90%. Collectively, this work demonstrates a coherent end-to-end route from the fully continuous synthesis of lenalidomide to CRBN ligand–linkers, providing versatile intermediates for subsequent batch preparation of PROTAC degraders. Compared to traditional batch synthesis, our integrated continuous flow system is more flexible and efficient, with shorter reaction times, simpler operation, enhanced safety, and greater potential for industrial-scale manufacturing.

## Methods

### General methods

The stepwise optimization for the synthesis of lenalidomide is described in section “3. Supplementary Method 1”, and that for pomalidomide in section “4. Supplementary Method 2”. Procedures for the continuous flow preparation of diverse CRBN ligand–linkers are given in section “5. Supplementary Method 3”. NMR datasets and spectra are available in sections 6 and 7 of Supplementary Information.

## Supplementary information

Supporting information

## Data Availability

All data generated during this study are included in this article and Supplementary Information. Experimental procedure, condition optimization, product characterization, and NMR spectra are provided in the Supplementary Information.
