# Supplementary material for: Accelerated synthesis of immunomodulatory imide drugs and their derivatives via continuous flow chemistry
Source: Commun Chem. 2026 Mar 3;9:154. doi: 10.1038/s42004-026-01956-1 (PMC13066112; doi:10.1038/s42004-026-01956-1)

Supporting Information

Accelerated synthesis of immunomodulatory imide drugs and their derivatives via continuous flow chemistry

Tianyi Hou[†,1], Junrong Huang[†,1,2], Yongheng Li[1], Yuxiang Zhu[*,1,2], Hengzhi You[*,1,2], Fen-Er Chen[*,2,3]

[1] School of Biomedical Engineering, Harbin Institute of Technology (Shenzhen), Taoyuan Street, Nanshan District, Shenzhen 518055, China.

[2] Green Pharmaceutical Engineering Research Center, Harbin Institute of Technology (Shenzhen), Taoyuan Street, Nanshan District, Shenzhen 518055, China.

[3] Engineering Center of Catalysis and Synthesis for Chiral Molecules, Department of Chemistry, Fudan University, Shanghai 200433, China.

† These authors contributed equally.

E-mail: youhengzhi@hit.edu.cn; zhuyuxiang@hit.edu.cn; rfchen@fudan.edu.cn

Table of Contents

1. General Information……………………………………………………………………..…………S1
2. Supplementary Note 1: flow experimental equipment information……………………..…………S1
3. Supplementary Method 1: continuous flow synthesis of lenalidomide……………….…………….……..S1
   3.1 Continuous flow synthesis of 2-bromo-methyl-3-nitrobenzoic acid methyl ester through photoreaction……………………………………………………………………………….……………S1
   3.2 Continuous flow synthesis of lenalidomide precursor **4**……………………………….….………..…S3
   3.3 Continuous flow hydrogenation of lenalidomide precursor **4**……..…………………..…………..S4
   3.4 A three-step continuous flow total synthesis of lenalidomide……………………….……….…...S6
4. Supplementary Method 2: continuous flow synthesis of pomalidomide………………...………...S6
   4.1 Solubility test of the pomalidomide precursor **4**……………………….……………………...…S6
   4.2 Continuous flow photochemical synthesis of pomalidomide precursor **6**………….……….……..S7
   4.3 Two-step continuous flow synthesis of pomalidomide………………………….….…….….…S8
5. Supplementary Method 3: continuous flow synthesis of CRBN ligand-linkers………....……………S9
   5.1 Continuous flow synthesis of compound **8**………………………..…………….……...….... S9
   5.2 General Method for continuous flow synthesis of CRBN ligand-linkers…………..………….. S10
6. Compound data.……………….....………………………………………………………..………S11
7. NMR spectra of compounds…..…………………………………………….……….……………S26

1. General Information

All reagents and solvents were purchased from commercial suppliers without further purification. All reactions were monitored by thin-layer chromatography (TLC) and NMR instrument. ^{1}H NMR, and ^{13}C NMR spectra were recorded using Q. One Instruments Quantum-I 400 M spectrometer and Bruker av-500 NMR instrument using $CDCl_3$ and DMSO-d_6 as the solvent. Chemical shift values are reported in ppm with the solvent resonance as the internal standard ($CDCl_3$: δ 7.26 for ^{1}H, δ 77.16 for ^{13}C; DMSO-d_6: δ 2.50 for ^{1}H, δ 39.52 for ^{13}C). Data are reported as follows: chemical shifts, multiplicity (s = singlet, d = doublet, t = triplet, q = quartet, br = broad, m = multiplet), coupling constants (Hz), and integration.

2. Supplementary Note 1: flow experimental equipment information

We employed an integrated continuous flow platform consisting of continuous feeding equipment, flow reactors, and a process-control unit, etc.

The feeding equipment: syringe pump (TYD02-04-CE, Lead Fluid Baoding Intelligent Equipment Manufacturing Co., Ltd., China); plunger pump (MPF0502C, Sanotac, China); peristaltic pump (LM60B; Runze Fluid, China).

Continuous reactors: stainless steel coil (0.8 mm i.d. 1.6 mm o.d., Runze Fluid, China); PTFE coil reactor (1.6 mm i.d., 3.2 mm o.d., Runze Fluid, China) and PTFE fittings (Runze Fluid, China).

Continuous system: continuous flow photochemical system (Shenzhen E-Zheng Tech. Co., Ltd.); continuous flow hydrogenation system (Oushisheng, China).

Process control unit: check valve (Runze Fluid, China; back-pressure regulator (Shenzhen E-Zheng Tech. Co., Ltd.).

Temperature control unit: magnetic hotplate stirrer (IKA C-MAG HS 7, IKA Instruments and Equipment Co., Ltd.)

3. Supplementary Method 1: continuous flow synthesis of lenalidomide

3.1 Continuous flow photochemical synthesis of 2-bromo-methyl-3-nitrobenzoic acid methyl ester

Supplementary table 1. Optimization table for bromination of **1** in flow. (a) Injection pump; (b) Continuous flow photochemical system; (c) Collection unit.

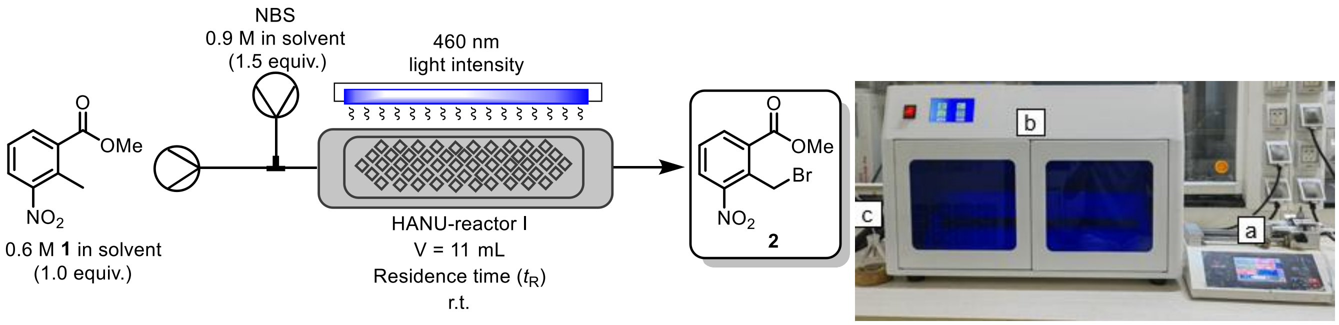

Entry	Solvent	Light intensity (W)	t_R (min)	Conv. (%)[a]
1	MeOAc	576	30	51
2	THF	576	30	2
3	Acetone	576	30	68
4	1,4-dioxane	576	30	4
5	DMF	576	30	-
6	MeCN	576	30	78
7	MeCN	460.8	30	90
8	MeCN	345.6	30	95
9	MeCN	230.4	30	93
10	MeCN	345.6	20	95
11	MeCN	345.6	10	88
12	MeCN	403.2	10	99 (96)[b]

Reaction conditions: All reactions were performed with 234.2 mg of **1** (1.2 mmol, 0.6 M) and 320.4 mg NBS (1.8 mmol, 0.9 M) in the specified solvent at room temperature using the flow setup shown above.

[a] Conversion was determined by ^{1}H NMR analysis of the crude mixture using 1,3,5-trimethoxybenzene as the internal standard.

[b] Isolated yield

With the lamp set to 576 W (100% power) and a residence time (t_R) of 30 min, 51% conversion of **1** to **2** was obtained (Entry 1). The solvent commonly used for batch reactions did not perform well in continuous flow reactions. We screened different bromination solvents and determined that acetonitrile (MeCN) was the best solvent, which increased the conversion to 78% (Entry 6). In subsequent experiments, increasing light intensity did not necessarily increase conversion, while the conversion increased from 78 to 90% when the light intensity decreased from 576 to 460.8 W (Entries 6-7). The reaction performed at 345.6 W provided superior results (95%, Entry 8). We examined the effect of residence time on the conversion, and ultimately, we determined the optimum condition with a residence time of 10 minutes and a light intensity of 403.2 W, which completely consumed **1** to provide the desired product **2** in 96% isolated yield (Entry 12).

Isolation procedure: After completion of the reaction, all the outflowing reaction mixture was collected

and saturated aqueous sodium sulfite was added. The mixture was stirred for 30 min, followed by extraction with ethyl acetate. The organic layer was washed with saturated brine, dried over anhydrous sodium sulfate, filtered, and concentrated. The crude product was purified by column chromatography using a certain ratio of ethyl acetate to *n*-hexane. The obtained product was dried under vacuum at 50 °C for 12 h to yield the final product.

3.2 Continuous flow synthesis of lenalidomide precursor 4

Supplementary table 2. Optimization table for the cyclization of **2** in flow. (a) Injection pump; (b) PTFE coil reactor and heating unit; (c) Collection unit.

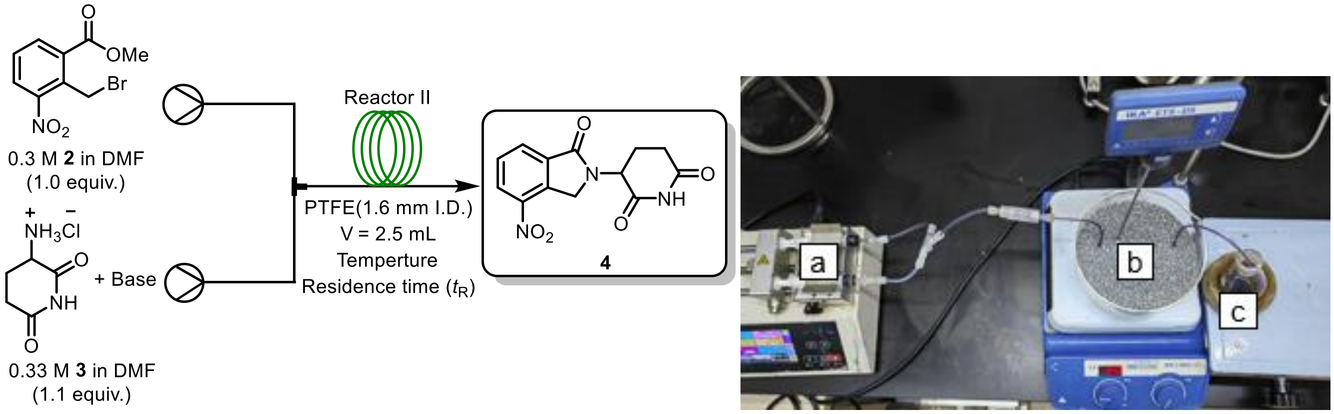

Entry	Base	Equiv.	Temp. (°C)	t_R (min)	[a] Yield (%)
1	Morpholine	3.0	80	30	13
2	TMEDA	3.0	80	30	20
3	Et_3N	3.0	80	30	61
4	Na_2CO_3	3.0	80	30	45
5	Imidazole	3.0	80	30	15
6	K_2CO_3	3.0	80	30	46
7	DIPEA	3.0	80	30	64
8	DIPEA	3.0	80	60	64
9	DIPEA	4.0	80	30	62
10	DIPEA	3.0	100	30	61
11[b]	DIPEA	3.0	80	30	83
12[b]	DIPEA	3.0	80	45	82

Reaction conditions: all reactions were performed using 246.6 mg of **2** (0.9 mmol, 0.3 M in DMF) and 162.9 mg of **3** (1.1 equiv., 0.33 M in DMF) under the flow setup shown above.

[a] Isolated yield.

[b] Before injection, a DMF solution of **3** and DIPEA was heated at 50 °C for 10 min until a blue-violet color was observed.

A continuous flow cyclization process was investigated to convert compound **2** into the desired 3-(4-nitro-

1-oxoisoindolin-2-yl) piperidine-2,6-dione (**4**) in a PTFE reactor II (2.5 mL work volume), as shown in Supplementary table 2. We prepared solutions of **2** (0.3 M, 1.0 equiv.) in DMF and the 3-aminopiperidine-2,6-dione hydrochloride (0.33 M, 1.1 equiv.) with an organic base in DMF separately. Due to low solubility of compound **3** in DMF, dissolution was assisted by ultrasonication or mechanical shaking after addition of an organic base. The two solutions were loaded into syringes, delivered by syringe pumps, and combined in a T-mixer. The reaction was carried out in a poly(tetrafluoroethylene) (PTFE) coil reactor II at a temperature of 80 °C. Notably, due to the limited solubility of reactant **3** in the organic solvent, we added an excess of the base and screened different types of bases. Optimization identified DIPEA as the optimal base, affording an isolated yield of 64% (Entry 7). Disappointingly, extending the residence time to 60 min and increasing the base loading to 4.0 equiv. did not improve the outcome, the isolated yields were 64% and 62% respectively (Entries 8-9). Further increasing the temperature to 100 °C with a residence time of 30 min was not beneficial (61% yield, Entry 10). We preheated **3** with DIPEA in DMF at 50 °C for 10 min until a blue–purple coloration developed, then the mixture was introduced into the reactor II at 80 °C with a residence time of 30 min, the desired product was obtained in 83% isolated yield (Entry 11).

Isolation procedure: The collected reaction mixture was concentrated under vacuum. Upon addition of deionized water, a solid precipitated; the solid was collected by filtration, washed with ethyl acetate (3×15 mL), and dried in a vacuum oven at 60 °C for 8 h to afford the final product.

3.3 Continuous flow hydrogenation of lenalidomide precursor 4

Supplementary table 3. Optimization table for the hydrogenation of **4** in flow. (a) Hydrogen generator; (b) Stirring and feeding unit; (c) Continuous flow hydrogenation reaction system; (d) Collection unit.

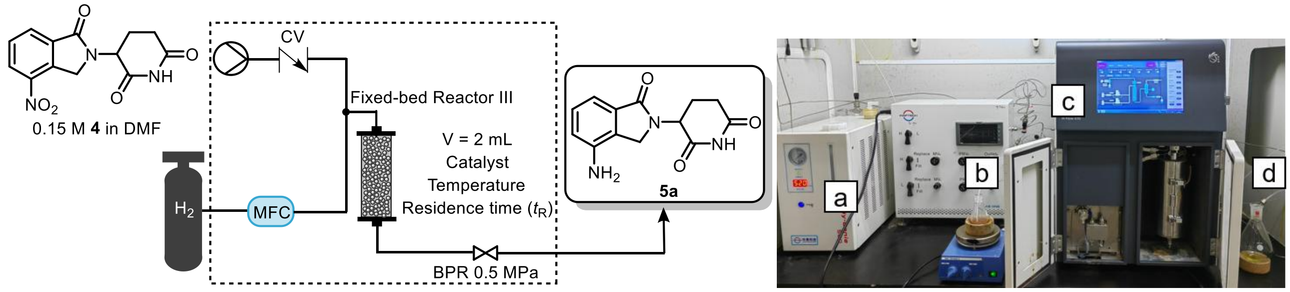

Entry	Catalyst	Temp. (°C)	[a] F (mL/min)	[b] Yield (%)
1	5% Pd/C	80	0.5	55
2	20% Ni/SiO_2	80	0.5	82
3	Raney Ni	80	0.5	99
4	5% Pd/Al_2O_3	80	0.5	99

5	5% Pd/Al_2O_3	80	1	86
6	5% Pd/Al_2O_3	100	1	99
7	5% Pd/Al_2O_3	100	2	78
8	5% Pd/Al_2O_3	120	2	77

Reaction conditions: all reactions were performed with 433.8 mg of **4** (1.5 mmol, 0.15 M in DMF) and H_2 gas (20 sccm) using the flow setup shown above.

[a] F: the flow rate of solution **4** (in DMF).

[b] Isolated yield.

We performed and optimized the hydrogenation of nitro group-containing substrate **4** in continuous flow, furnishing lenalidomide (Supplementary table 3). A solution of **4** (0.15 M) in DMF was combined with H_2 (20 sccm) and passed through a packed-bed continuous flow hydrogenation reactor (Oushisheng, China) charged with 5% Pd/C. The flow system was operated at 80 °C with a residence time of 4 min, and pressurized by an adjustable back-pressure regulator at 0.5 MPa. With all other reaction parameters held constant, the following heterogeneous catalysts were evaluated: 5% Pd/C, 20% Ni/SiO_2, Raney Ni, and 5% Pd/Al_2O_3. Both Raney Ni and 5% Pd/Al_2O_3 delivered high conversion (99%, Entries 3-4). Because Raney Ni is pyrophoric, 5% Pd/Al_2O_3 was selected as the catalyst for improved safety. Subsequently, we attempted to reduce the residence time by increased the reaction temperature. Performing the reaction with 5% Pd/Al_2O_3 as the catalyst at 100 °C and 0.5 MPa of H_2, the residence time was able to be shortened to 2 min. Complete consumption of **4** was achieved, affording **5a** in 99% isolated yield (Entry 6).

Isolation procedure: The collected reaction mixture was concentrated under vacuum. Upon addition of deionized water, a solid precipitated; the solid was collected by filtration, washed with ethyl acetate (3×15 mL), and dried in a vacuum oven at 60 °C for 8 h to afford the final product.

The synthesis and isolation of lenalidomide derivatives 5b-5e were following the same procedures as lenalidomide 5a.

3.4 A three-step continuous flow total synthesis of lenalidomide

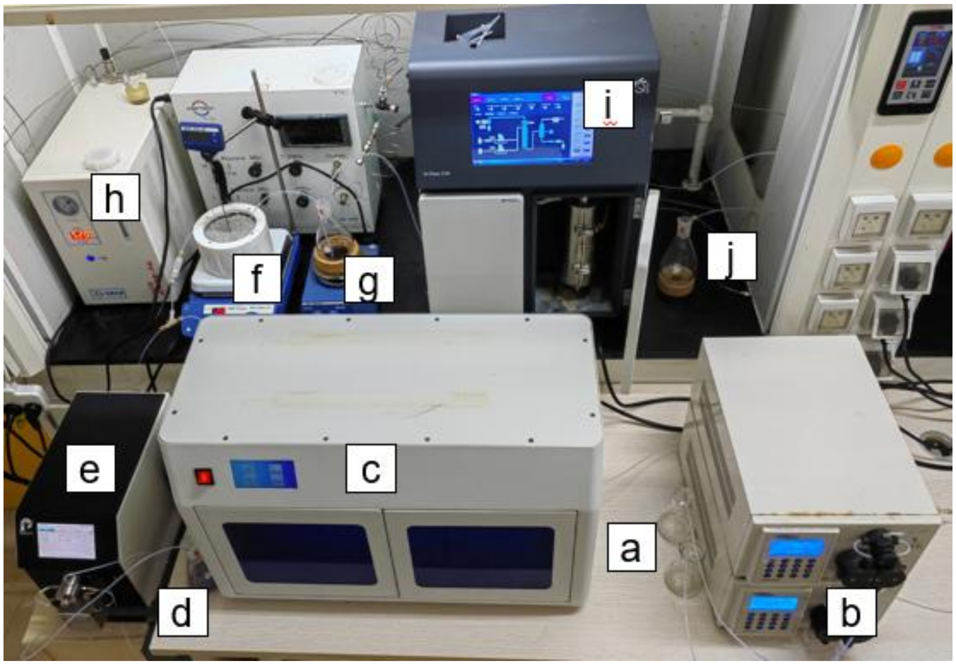

Supplementary figure 1. Schematic diagram of the apparatus for continuous flow synthesis of lenalidomide. (a), (d) Feeding unit; (b), (e) Plunger pump; (c) Continuous flow photochemical system; (f) PTFE coil reactor and heating unit; (g), (j) Collection unit; (h) Hydrogen generator; (i) Continuous flow hydrogenation reaction system.

4. Supplementary Method 2: continuous flow synthesis of pomalidomide

4.1 Solubility test of the pomalidomide precursor 4

Supplementary table 4. 4.1 Solubility test of the pomalidomide precursor **4**.[a]

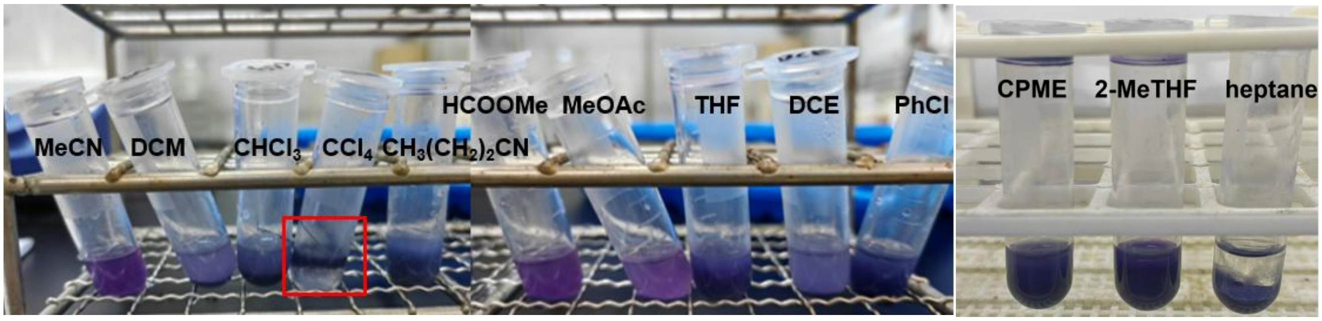

Solvent	0.05 M **4** at r.t.	0.05 M **4** at 50 ℃	0.025 M **4** at r.t.	0.025 M **4** at 50 ℃
MeCN	I	I	I	I
DCM	I	I	I	I
$CHCl_3$	I	I	I	I

CCl_4	I	I	I	I
$CH_3(CH_2)_2CN$	I	I	I	I
HCOOMe	I	I	I	I
MeOAc	I	I	I	I
THF	I	I	I	I
DCE	I	I	I	I
PhCl	I	I	I	I
CPME	I	I	I	I
2-MeTHF	I	I	I	I
heptane	I	I	I	I

[a] I: insoluble S: soluble

Experimental procedures: To a sample of **4** (14.4 mg, 0.050 mmol) was added 1.0 or 2.0 mL of the specified solvent. The resulting mixture was then sonicated at the corresponding temperature for 10 min. Although no solvent was able to properly dissolve **4**, we found that **4** forms a suspension in carbon tetrachloride (CCl_4), as highlighted by the red box in Supplementary table 4.

4.2 Continuous flow photochemical synthesis of pomalidomide precursor 6

Supplementary table 5. Optimization for the synthesis of **6** from **4** in flow. (a) Stirring and feeding unit; (b) Peristaltic pump; (c) Continuous flow photochemical system; (d) Collection unit.

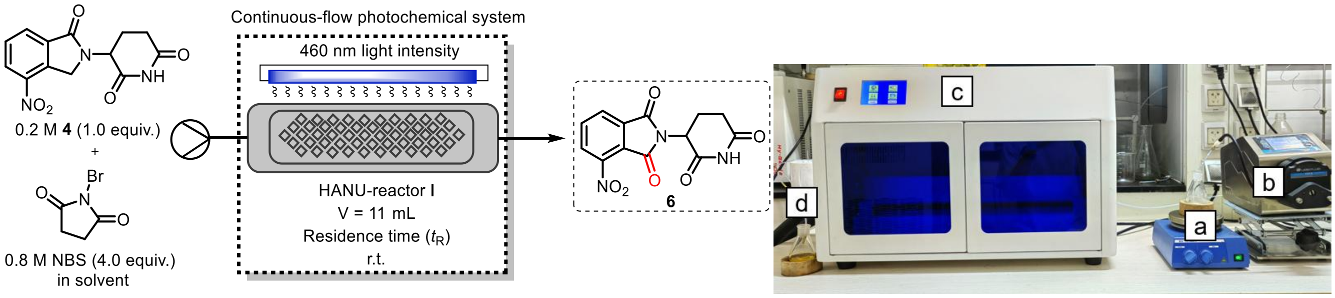

Entry	Solvent	Light intensity (W)	Temp. (°C)	t_R (min)	[a] Yield (%)
1	$MeCN:H_2O$ = 1:0.1	403.2	r.t.	30	48
2	$MeCN:H_2O$ = 1:0.1	576	r.t.	30	60
3	$MeCN:CCl_4:H_2O$ = 1:1:0.1	576	r.t.	30	98
4	$MeCN:CCl_4:H_2O$ = 1:1:0.1	576	r.t.	20	98
5	$MeCN:CCl_4:H_2O$ = 1:1:0.1	576	r.t.	10	98
6	$MeCN:CCl_4:H_2O$ = 1:1:0.1	576	r.t.	5	60

Reaction conditions: all reactions were performed using 289.2 mg of **4** (1 mmol, 0.2 M) and NBS (4.0 equiv., 0.8 M) under the flow setup shown above.

[a] isolated yield

Isolation procedure: The collected reaction mixture was concentrated under vacuum. Upon addition of deionized water, a solid precipitated; the solid was collected by filtration, washed with ethyl acetate (3×15 mL), and dried in a vacuum oven at 60 °C for 8 h to afford the final product.

4.3 Two-step continuous flow synthesis of pomalidomide

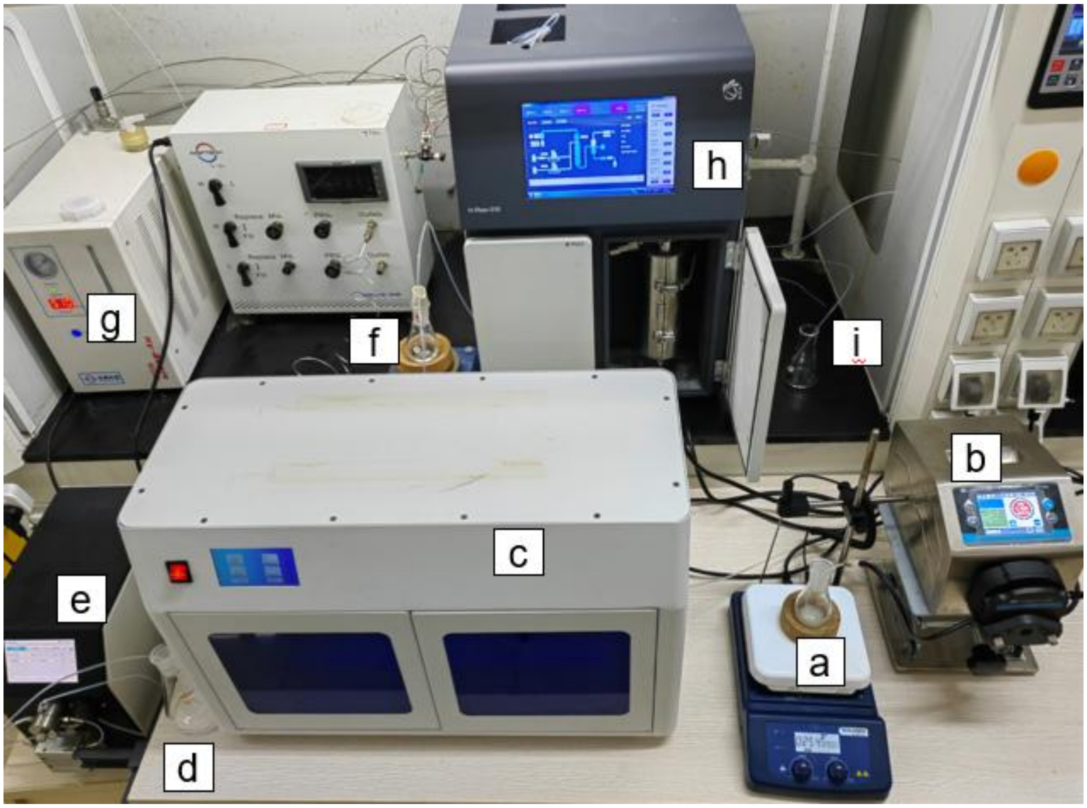

Supplementary figure 2. Schematic diagram of the apparatus for continuous flow synthesis of pomalidomide. (a), (d) Feeding unit; (b) Peristaltic pump; (c) Continuous flow photochemical system; (e) Plunger pump; (f) Collection and Stirring unit; (g) Hydrogen generator; (h) Continuous flow hydrogenation reaction system; (i) Collection unit.

5. Supplementary Method 3: continuous flow synthesis of CRBN ligand-linkers

5.1 Continuous flow synthesis of compound 8

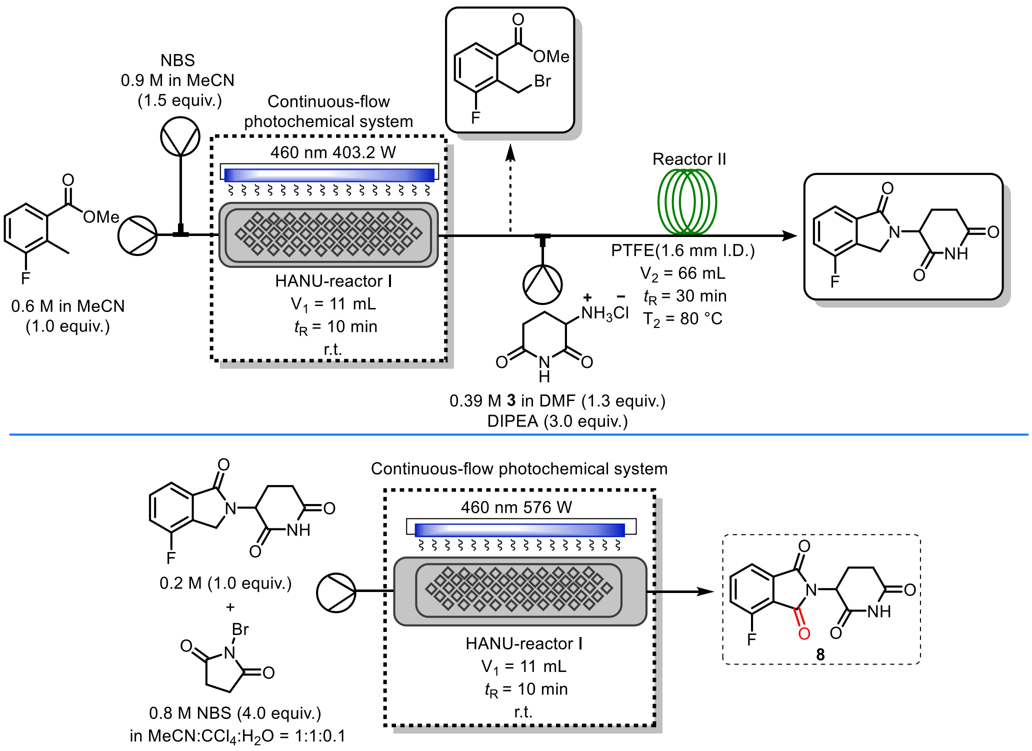

Supplementary figure 3. Schematic diagram for the continuous flow synthesis of compound **8**

Experimental procedures: MeCN solutions of methyl 3-fluoro-2-methylbenzoate (0.6 M, 1.0 equiv.) and NBS (0.9 M, 1.5 equiv.) were combined in a T-mixer. The combined stream was fed into a continuous flow photochemical system to perform photoinitiated bromination in flow. The system comprised HANU Reactor I (11 mL work volume), an LED light source equipped with a recirculating chiller, and associated optics. The stream irradiated at 460 nm at room temperature (lamp power 403.2 W) with a residence time (t_R) of 10 min. Upon exiting HANU Reactor I, the reaction solution was merged via a T-mixer with a DMF solution of **3** (0.39 M, 1.3 equiv.) and DIPEA (0.90 M, 3.0 equiv.) preheated at 50 °C for 10 min to develop a blue–purple coloration, and the combined stream was delivered directly to PTFE Reactor II (1.6 mm i.d., 66 mL work volume) at 80 ℃ with a residence time of 30 min.

Evaporate the collected reaction mixture under reduced pressure to remove the solvent, add water to precipitate the solid, and filter to obtain 3-(4-fluoro-1-oxoisoindolin-2-yl) piperidine-2,6-dione. In accordance with the optimized oxidation protocol, the prepared reaction mixture was introduced into the continuous flow photoreactor. The reactor was irradiated at 460 nm (lamp power 576 W) at room

temperature with a residence time for 10 min, affording 6 in 90% isolated yield.

Isolation procedure: The collected reaction mixture was concentrated under vacuum. Upon addition of deionized water, a solid precipitated; the solid was collected by filtration, washed with ethyl acetate (3×15 mL), and dried in a vacuum oven at 60 °C for 8 h to afford the final product.

5.2 General Method for the continuous flow synthesis of CRBN ligand-linkers

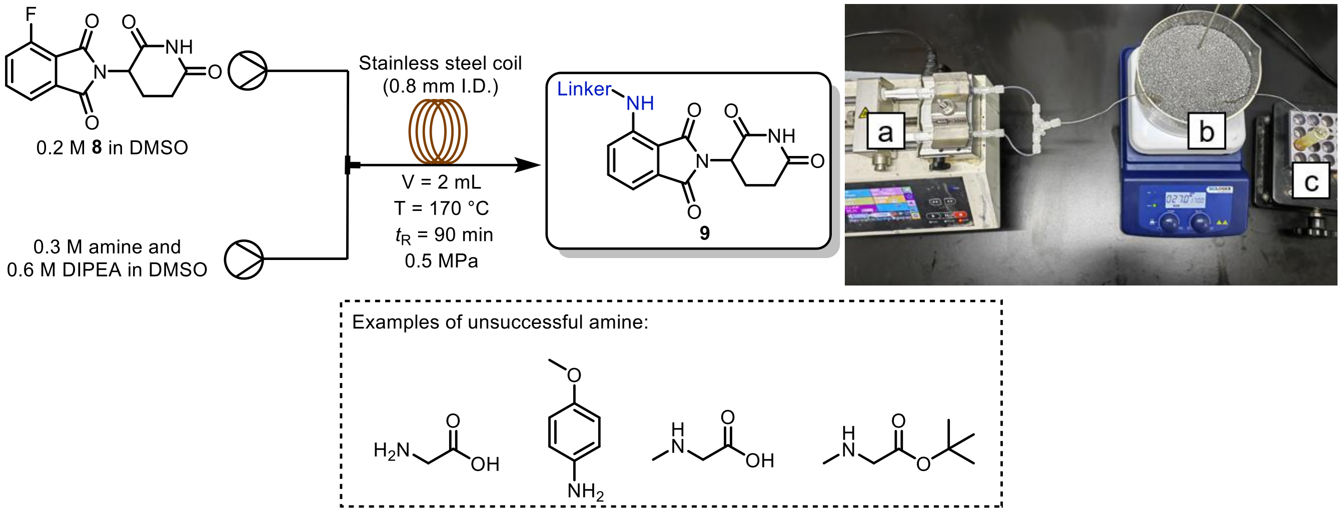

Supplementary figure 4. Synthesis of compound **9** via S_NAr reaction in flow. (a) Injection pump; (b) Stainless steel Coil reactor and heating unit; (c) Collection unit.

Experimental procedures: Solutions of amines (0.30 M, 1.5 equiv.) and DIPEA (0.60 M, 3.0 equiv.) in DMSO were combined via a T-mixer with a DMSO solution of **8** (0.20 M) and fed into a stainless-steel coil reactor. The reaction was operated at 170 °C with a residence time of 90 min under 0.5 MPa back-pressure.

Isolation Procedures: The reactor outflow was collected, concentrated under vacuum, and purified by silica gel column chromatography (*n*-hexane/ethyl acetate) to give CRBN ligand-linkers. The product was dried in a vacuum oven for 8 h to afford the final product.

6. Compound data

methyl 2-(bromomethyl)-3-nitrobenzoate (2)

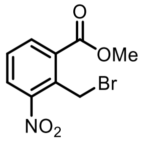

2

The synthesis was performed using the apparatus specified in supplementary table 1. Methyl 2-methyl-3-nitrobenzoate (0.234 g, 1.2 mmol, 0.6 M) and N-bromosuccinimide (NBS, 0.320 g, 1.8 mmol, 0.9 M) were accurately weighed and placed into separate 10 mL centrifuge tubes. To each tube, 2 mL of solvent was added. The mixtures were vortexed until clear solutions formed. Each solution was then withdrawn into a 5 mL polyethylene syringe. The two syringes containing the reaction solutions were mounted onto a syringe pump. The syringe size (5 mL) and flow rates were set in the pump software. The light intensity of the continuous-flow photoreactor was set to 403.2 W. After completing all configurations, the instrument was started, and the effluent was collected as the final product.

After completion of the reaction, all the outflowing reaction mixture was collected and saturated aqueous sodium sulfite was added. The mixture was stirred for 30 min, followed by extraction with ethyl acetate. The organic layer was washed with saturated brine, dried over anhydrous sodium sulfate, filtered, and concentrated. The crude product was purified by column chromatography using a certain ratio of ethyl acetate to *n*-hexane. The obtained product was dried under vacuum at 50 °C for 12 h to yield the final product.

Compound **2** was isolated as a pale-yellow crystals; ^{1}H NMR (400 MHz, $CDCl_3$) δ 8.10 (dd, *J* = 7.9, 1.4 Hz, 1H), 7.95 (dd, *J* = 8.2, 1.4 Hz, 1H), 7.53 (t, *J* = 8.0 Hz, 1H), 5.15 (s, 2H), 3.99 (s, 3H); ^{13}C NMR (101 MHz, $CDCl_3$) δ 165.9, 150.6, 134.8, 132.7, 132.4, 129.2, 127.9, 53.1, 22.8. HRMS (ESI): m/z calculated for $C_9H_9BrNO_4$ $[M+H]^+$ = 273.9709, found 273.9712.

3-(4-nitro-1-oxoisoindolin-2-yl)piperidine-2,6-dione (4)

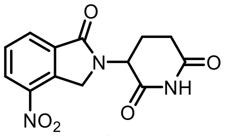

4

The synthesis was conducted using the setup described in supplementary table 2. Compound **2** (0.246 g, 0.9 mmol, 0.3 M) and compound **3** (0.163 g, 1.8 mmol, 0.9 M) were accurately weighed and placed into separate 10 mL centrifuge tubes. To each tube, 3 mL of N,N-dimethylformamide and the organic base DIPEA were added. Dissolution was assisted by ultrasonication or mechanical agitation. A solution containing **3** and DIPEA was pre-warmed at 50 °C for 10 minutes before being drawn into a 5 mL polyethylene syringe. Both syringes containing the reaction solutions were mounted onto a syringe pump. The syringe specifications and flow rates were set accordingly. The reactor was heated to 80 °C, and the solutions were delivered via the syringe pump and mixed in a T-mixer. The reaction proceeded in a polytetrafluoroethylene (PTFE) coil reactor II. The effluent was collected as the crude product.

The collected reaction mixture was concentrated under vacuum. Upon addition of deionized water, a solid

precipitated; the solid was collected by filtration, washed with ethyl acetate (3×15 mL), and dried in a vacuum oven at 60 °C for 8 h to afford the final product.

Compound **4** was isolated as a gray-white solid; ^{1}H NMR (400 MHz, DMSO-d_6) δ 11.05 (s, 1H), 8.47 (d, *J* = 8.1 Hz, 1H), 8.19 (d, *J* = 7.4 Hz, 1H), 7.84 (t, *J* = 7.8 Hz, 1H), 5.18 (dd, *J* = 13.2, 5.1 Hz, 1H), 4.98 – 4.77 (m, 2H), 2.92 (ddd, *J* = 18.0, 13.4, 5.4 Hz, 1H)., 2.65 – 2.53 (m, 2H), 2.03 – 2.01 (m, 1H); ^{13}C NMR (101 MHz, DMSO-d_6) δ 172.8, 170.7, 165.9, 143.3, 137.4, 134.7, 130.2, 129.6, 127.1, 51.8, 48.4, 31.2, 22.3; HRMS (ESI): m/z calculated for $C_{13}H_{11}N_3NaO_5$ $[M+Na]^+$ = 312.0591, found 312.0595.

3-(4-amino-1-oxoisoindolin-2-yl)piperidine-2,6-dione (5a)

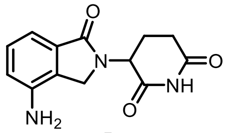

5a

The uninterrupted continuous flow synthesis of lenalidomide derivatives **5a**–**5e**was conducted using the setup shown in supplementary figure 1. The detailed synthetic procedure is provided in the main text.

Compound **5a** was isolated as a slightly yellow solid; ^{1}H NMR (400 MHz, DMSO-d_6) δ 11.02 (s, 1H), 7.19 (t, *J* = 7.7 Hz, 1H), 6.91 (d, *J* = 7.3 Hz, 1H), 6.79 (d, *J* = 7.8 Hz, 1H), 5.43 (s, 2H), 5.11 (dd, *J* = 13.3, 5.1 Hz, 1H), 4.27 – 4.05 (m, 2H), 2.92 (ddd, *J* = 18.2, 13.6, 5.4 Hz, 1H), 2.61 (d, *J* = 17.8 Hz, 1H), 2.30 (qd, *J* = 13.2, 4.4 Hz, 1H), 2.04 – 1.99 (m, 1H); ^{13}C NMR (101 MHz, DMSO-d_6) δ 173.0, 171.3, 169.0, 143.7, 132.3, 128.9, 125.7, 116.5, 110.5, 51.6, 45.6, 31.3, 22.8; HRMS (ESI): m/z calculated for $C_{13}H_{13}N_3NaO_3$ $[M+Na]^+$ = 282.0849, found 282.0853.

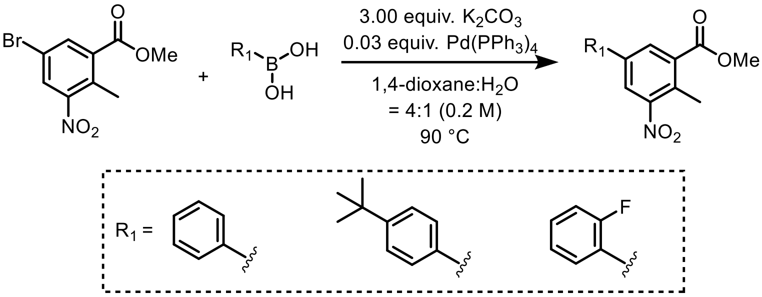

*Precursors for the synthesis of **5b**, **5c**, and **5e** were all prepared using the following procedure. Methyl 5-fluoro-2-methyl-3-nitrobenzoate, which was used for the synthesis of compound **5d**, is directly available from commercial supplier.*

Methyl 5-bromo-2-methyl-3-nitrobenzoate (10 mmol, 1.00 equiv.), the corresponding phenylboronic acid derivative (15 mmol, 1.50 equiv.), tetrakis(triphenylphosphine)palladium(0) (0.3 mmol, 0.03 equiv.), and potassium carbonate (45 mmol, 3.00 equiv.) were added into a reaction tube containing a stir bar. The tube was purged with argon three times. A solvent mixture (50 mL, 1,4-dioxane:water = 4:1) was added at room temperature, and the reaction was stirred and heated under reflux (90 °C) for 8 h. After completion, the reaction mixture was allowed to cool to room temperature. Insoluble solids were removed by filtration. The filtrate was diluted with water and extracted with ethyl acetate three times. The combined organic layers were washed sequentially with 1 M NaOH aqueous solution and brine. The organic phase was dried over anhydrous sodium sulfate, filtered, and concentrated under reduced pressure to afford the crude

product. Purification by column chromatography yielded the desired substrate for subsequent synthesis.

methyl 4-methyl-5-nitro-[1,1'-biphenyl]-3-carboxylate

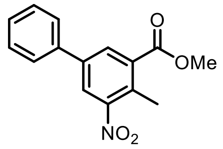

This substrate was used for the synthesis of compound ***5b***. Compound was isolated as a slightly yellow solid; ^{1}H NMR (400 MHz, $CDCl_3$) δ 8.21 (s, 1H), 8.05 (s, 1H), 7.59 (d, J = 8.4 Hz, 2H), 7.48 (t, J = 7.5 Hz, 2H), 7.42 (td, J = 7.5, 1.5 Hz, 1H), 3.97 (s, 3H), 2.64 (s, 3H); ^{13}C NMR (101 MHz, $CDCl_3$) δ 166.9, 152.5, 139.9, 137.6, 133.8, 132.0, 131.6, 129.3, 128.8, 127.0, 124.9, 52.7, 16.1; HRMS (ESI): m/z calculated for $C_{15}H_{14}NO_4$ $[M+H]^+$ = 272.0917, found 272.0926.

methyl 4'-(tert-butyl)-4-methyl-5-nitro-[1,1'-biphenyl]-3-carboxylate

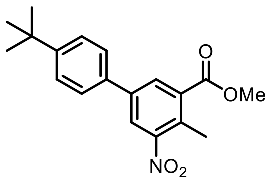

This substrate was used for the synthesis of compound ***5c***. Compound was isolated as a slightly yellow solid; ^{1}H NMR (400 MHz, $CDCl_3$) δ 8.21 (d, J = 2.0 Hz, 1H), 8.06 (d, J = 2.0 Hz, 1H), 7.52 (q, J = 8.5 Hz, 4H), 3.96 (s, 3H), 2.64 (s, 3H), 1.37 (s, 9H); ^{13}C NMR (101 MHz, $CDCl_3$) δ 167.1, 152.5, 152.1, 139.8, 134.8, 133.8, 131.9, 131.2, 126.7, 126.3, 124.8, 52.7, 34.8, 31.4, 16.1; HRMS (ESI): m/z calculated for $C_{19}H_{21}NO_4$ $[M+H]^+$ = 328.1543, found 328.1558.

methyl 2'-fluoro-4-methyl-5-nitro-[1,1'-biphenyl]-3-carboxylate

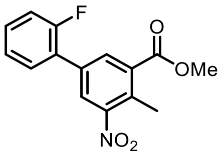

This substrate was used for the synthesis of compound ***5e***. Compound was isolated as a slightly yellow solid; ^{1}H NMR (400 MHz, $CDCl_3$) δ 8.21 (s, 1H), 8.07 (s, 1H), 7.47 (t, J = 7.7 Hz, 1H), 7.44 – 7.38 (m, 1H), 7.29 (d, J = 7.5 Hz, 1H), 7.24 – 7.17 (m, 1H), 3.99 (s, 3H), 2.68 (s, 3H); ^{13}C NMR (101 MHz, $CDCl_3$) δ 166.7, 159.70 (d, J = 249.2 Hz), 152.1, 134.5 , 133.9 (d, J = 3.3 Hz), 133.5 , 132.2 , 130.6 (d, J = 8.3 Hz), 130.3 (d, J = 2.9 Hz), 126.9 (d, J = 3.7 Hz), 125.6 (d, J = 12.8 Hz), 124.9 (d, J = 3.8 Hz), 116.5 (d, J = 22.2 Hz), 52.7, 16.1; ^{19}F NMR (376 MHz, DMSO-d_6) δ -118.31 – -118.41 (m). HRMS (ESI): m/z calculated for $C_{15}H_{12}FNO_4$ $[M+H]^+$ = 290.0823, found 290.0829.

3-(4-amino-1-oxo-6-phenylisoindolin-2-yl)piperidine-2,6-dione (5b)

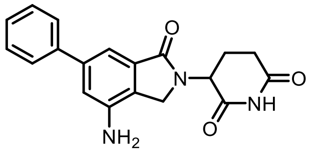

5b

Compound was isolated as a slightly yellow solid; ^{1}H NMR (400 MHz, DMSO-d_6) δ 11.03 (s, 1H), 7.62 (d, J = 7.6 Hz, 2H), 7.46 (t, J = 7.5 Hz, 2H), 7.36 (t, J = 7.4 Hz, 1H), 7.14 (s, 1H), 7.09 (s, 1H), 5.59 (s, 2H), 5.12 (d, J = 5.0 Hz, 1H), 4.45 – 4.06 (m, 2H), 3.00 – 2.86 (m, 1H), 2.62 (d, J = 17.6 Hz, 1H), 2.41 – 2.26 (m, 1H), 2.09 – 2.01 (m, 1H); ^{13}C NMR (101 MHz, DMSO-d_6) δ 172.9, 171.2, 168.8, 144.0, 141.4, 140.4, 133.1, 128.9, 127.5, 126.7, 125.0, 114.9, 108.7, 51.6, 45.5, 31.2, 22.7; HRMS (ESI): m/z calculated for $C_{19}H_{17}N_3NaO_3$ [M+Na]$^+$ 358.1162, found 358.1169 (small amount of DMF was presented in the NMR).

3-(4-amino-6-(4-(tert-butyl)phenyl)-1-oxoisoindolin-2-yl)piperidine-2,6-dione (5c)

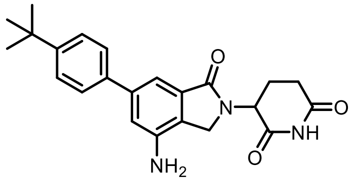

5c

Compound was isolated as a slightly yellow solid; ^{1}H NMR (400 MHz, DMSO-d_6) δ 11.01 (s, 1H), 7.59 – 7.44 (m, 4H), 7.14 (s, 1H), 7.08 (s, 1H), 5.54 (s, 2H), 5.13 (dd, J = 13.3, 5.1 Hz, 1H), 4.30 – 4.11 (m, 2H), 2.93 (ddd, J = 18.1, 13.4, 5.3 Hz, 1H), 2.62 (d, J = 17.3 Hz, 1H), 2.32 (tt, J = 13.3, 6.6 Hz, 1H), 2.08 – 2.01 (m, 1H), 1.32 (s, 9H); ^{13}C NMR (101 MHz, DMSO-d_6) δ 172.9, 171.2, 168.8, 162.3, 149.9, 144.0, 141.2, 137.4, 133.0, 126.3, 125.7, 124.8, 114.7, 108.4, 51.6, 45.5, 35.8, 31.1, 22.7; HRMS (ESI): m/z calculated for $C_{23}H_{25}N_3NaO_3$ [M+Na]$^+$ 414.1788, found 414.1782.

3-(4-amino-6-fluoro-1-oxoisoindolin-2-yl)piperidine-2,6-dione (5d)

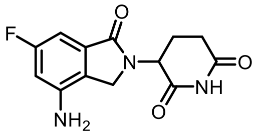

5d

Compound was isolated as a slightly yellow solid; ^{1}H NMR (400 MHz, DMSO-d_6) δ 11.01 (s, 1H), 6.61 (dd, J = 7.7, 2.2 Hz, 1H), 6.56 (dd, J = 11.9, 2.3 Hz, 1H), 5.80 (s, 2H), 5.09 (dd, J = 13.3, 5.1 Hz, 1H), 4.23 – 4.03 (m, 2H), 2.91 (ddd, J = 18.3, 13.6, 5.4 Hz, 1H), 2.61 (d, J = 15.8 Hz, 1H), 2.29 (qd, J = 13.2, 4.5 Hz, 1H), 2.07 – 2.00 (m, 1H); ^{13}C NMR (101 MHz, DMSO-d6) δ 172.8, 171.1, 167.9 (d, J = 4.0 Hz), 163.3 (d, J = 240.5 Hz), 145.4 (d, J = 11.9 Hz), 133.7 (d, J = 11.0 Hz), 121.8, 102.5 (d, J = 26.1 Hz), 96.2 (d, J = 24.1 Hz), 51.7, 45.4, 31.2, 22.7; ^{19}F NMR (376 MHz, DMSO-d_6) δ -113.70 (t, J = 10.2 Hz); HRMS (ESI): m/z calculated for $C_{13}H_{12}FN_3NaO_3$ [M+Na]$^+$ = 300.0755, found 300.0758.

3-(4-amino-6-(2-fluorophenyl)-1-oxoisoindolin-2-yl)piperidine-2,6-dione (5e)

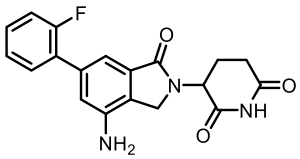

5e

Compound was isolated as a slightly yellow solid; ^{1}H NMR (400 MHz, DMSO-d_6) δ 11.04 (s, 1H), 7.51 (t, J = 7.9 Hz, 1H), 7.45 – 7.39 (m, 1H), 7.34 – 7.26 (m, 2H), 7.03 (s, 1H), 6.98 (s, 1H), 5.61 (s, 2H), 5.14 (dd, J = 13.4, 5.1 Hz, 1H), 4.33 – 4.10 (m, 2H), 3.06 – 2.86 (m, 1H), 2.62 (d, J = 17.2 Hz, 1H), 2.33 (dd, J = 13.2, 4.4 Hz, 1H), 2.11 – 1.97 (m, 1H); ^{13}C NMR (101 MHz, DMSO-d_6) δ 172.9, 171.2, 168.6, 159.05 (d, J = 245.6 Hz), 143.7, 135.9, 132.7, 130.7 (d, J = 2.8 Hz), 129.5 (d, J = 7.9 Hz), 128.4 (d, J = 13.2 Hz), 125.3, 124.9 (d, J = 3.0 Hz), 116.8, 116.14 (d, J = 22.8 Hz), 110.7, 51.6, 45.6, 31.3, 22.7; ^{19}F NMR (376 MHz, DMSO-d_6) δ -118.04; HRMS (ESI): m/z calculated for $C_{19}H_{16}FN_3NaO_3$ $[M+Na]^+$ = 376.1068, found 376.1074.

2-(2,6-dioxopiperidin-3-yl)-4-nitroisoindoline-1,3-dione (6)

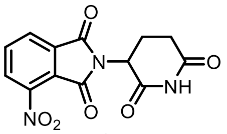

6

The synthesis was performed using the apparatus described in Supplementary Table 5. A mixture of compound **4** (289.2 mg, 1 mmol, 0.2 M) and N-bromosuccinimide (4.0 equiv., 0.8 M) was placed in a vial. The solvent system MeCN:CCl_4:H_2O = 1:1:0.1 was added. The photoreactor was set to a light intensity of 576 W, with a flow rate of 1.1 mL/min and a residence time of 10 minutes. The collected reaction mixture was concentrated under reduced pressure. Deionized water was added to the residue, resulting in the precipitation of a solid. The solid was collected by filtration, washed with ethyl acetate (3 × 15 mL), and dried in a vacuum oven at 60 °C for 8 h.

Compound **6** was isolated as a white solid; ^{1}H NMR (400 MHz, DMSO-d_6) δ 11.17 (s, 1H), 8.35 (d, J = 8.1 Hz, 1H), 8.24 (d, J = 7.5 Hz, 1H), 8.12 (t, J = 7.8 Hz, 1H), 5.20 (dd, J = 12.9, 5.4 Hz, 1H), 2.96 – 2.82 (m, 1H), 2.63 – 2.56 (m, 2H), 2.10 – 2.04 (m, 1H); ^{13}C NMR (101 MHz, DMSO-d_6) δ 172.7, 169.5, 165.2, 162.5, 144.4, 136.8, 133.0, 128.9, 127.3, 122.6, 49.4, 30.9, 21.7. HRMS (ESI): m/z calculated for $C_{13}H_9N_3NaO_6$ $[M+Na]^+$ = 326.0384, found 326.0388.

4-amino-2-(2,6-dioxopiperidin-3-yl)isoindoline-1,3-dione (7)

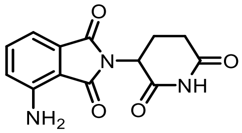

7

The uninterrupted continuous flow synthesis of pomalidomide **7** was carried out using the apparatus depicted in Supplementary figure 2. The corresponding synthetic method is described in the main text.

Compound **7** was isolated as a yellow solid; ^{1}H NMR (400 MHz, DMSO-d_6) δ 11.09 (s, 1H), 7.51 – 7.43 (m, 1H), 7.01 (t, J = 7.0 Hz, 2H), 6.52 (s, 2H), 5.05 (dd, J = 13.0, 5.4 Hz, 1H), 2.93 – 2.83 (m, 1H), 2.61

– 2.53 (m, 2H), 2.04 – 1.99 (m, 1H); ^{13}C NMR (101 MHz, DMSO-d_6) δ 172.8, 170.1, 168.6, 167.4, 146.7, 135.5, 132.0, 121.7, 111.0, 108.5, 48.5, 31.0, 22.1; HRMS (ESI): m/z calculated for $C_{13}H_{11}N_3NaO_4$ $[M+Na]^+$ = 296.0642, found 296.0648.

2-(2,6-dioxopiperidin-3-yl)-4-fluoroisoindoline-1,3-dione (8)

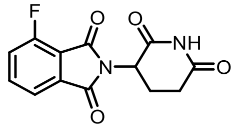

The synthesis of compound 8 was performed following the procedure illustrated in Supplementary figure 3. Compound **8** as a grayish-white solid, ^{1}H NMR (400 MHz, DMSO-d_6) δ 11.16 (s, 1H), 7.99 – 7.88 (m, 1H),, 7.78 (d, J = 7.3 Hz, 1H), 7.72 (t, J = 8.9 Hz, 1H), 5.16 (dd, J = 13.0, 5.4 Hz, 1H), 2.89 (td, J = 13.8, 13.3, 6.8 Hz, 1H), 2.67 – 2.52 (m, 2H), 2.13 – 2.00 (m, 1H); ^{13}C NMR (101 MHz, DMSO-d_6) δ 172.98, 169.89, 166.30 (d, J = 3.0 Hz), 164.15, 156.96 (d, J = 262.3 Hz), 138.24 (d, J = 7.9 Hz), 133.56, 123.16 (d, J = 19.6 Hz), 120.21 (d, J = 3.2 Hz), 117.14 (d, J = 12.5 Hz), 49.22, 31.02, 21.97; ^{19}F NMR (376 MHz, DMSO-d_6) δ -114.64 (d, J = 9.3 Hz); HRMS (ESI): m/z calculated for $C_{13}H_{11}FN_2NaO_3$ $[M+Na]^+$ = 285.0646, found 285.0649.

2-(2,6-dioxopiperidin-3-yl)-4-(prop-2-yn-1-ylamino)isoindoline-1,3-dione (9a)

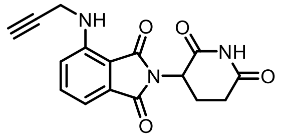

9a

Compounds **9a**–**9s** were prepared following the method illustrated in Supplementary figure 4. Compound **9a** was isolated as a yellow solid, (0.175 g, 94%); ^{1}H NMR (400 MHz, DMSO-d_6) δ 11.08 (s, 1H), 7.60 (t, J = 7.8 Hz, 1H), 7.09 (dd, J = 15.6, 7.8 Hz, 2H), 6.89 (t, J = 6.3 Hz, 1H), 5.03 (dd, J = 12.9, 5.3 Hz, 1H), 4.13 (dd, J = 6.3, 2.4 Hz, 2H), 3.12 (s, 1H), 2.85 (ddd, J = 19.4, 14.0, 5.3 Hz, 1H), 2.59 – 2.52 (m, 2H), 2.03 – 1.98 (m, 1H); ^{13}C NMR (101 MHz, DMSO-d_6) δ 172.9, 170.1, 168.6, 167.3, 145.2, 136.1, 132.2, 118.0, 111.4, 110.3, 80.9, 73.8, 48.6, 31.5, 31.0, 22.2; HRMS (ESI) m/z calculated for $C_{16}H_{13}N_3NaO_4$ $[M+Na]^+$ = 334.0798, found 334.0789.

tert-butyl-4-(((2-(2,6-dioxopiperidin-3-yl)-1,3-dioxoisoindolin-4-yl)amino)methyl)piperidine-1-carboxylate (9b)

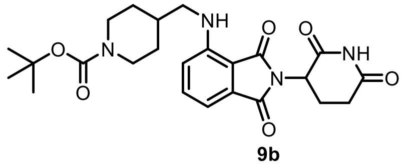

9b

Compound **9b** was isolated as a yellow solid, (0.265 g, 94%); ^{1}H NMR (400 MHz, DMSO-d_6) δ 11.07 (s, 1H), 7.52 (dd, J = 8.6, 7.1 Hz, 1H), 7.09 (d, J = 8.6 Hz, 1H), 6.97 (d, J = 7.0 Hz, 1H), 6.58 (t, J = 6.3 Hz, 1H), 5.01 (dd, J = 12.8, 5.4 Hz, 1H), 3.90 (d, J = 10.7 Hz, 2H), 3.17 (t, J = 6.5 Hz, 2H), 2.91 – 2.77 (m, 1H), 2.59 – 2.51 (m, 2H), 2.03 – 1.94 (m, 1H), 1.78 – 1.66 (m, 1H), 1.66 – 1.58 (m, 2H), 1.34 (s, 9H)1.21 – 1.09 (m, 2H), 1.08 – 0.96 (m, 2H); ^{13}C NMR (101 MHz, DMSO-d_6) δ 172.9, 170.2, 169.0, 167.3, 153.9,

146.6, 136.3, 132.2, 117.4, 110.5, 109.0, 78.6, 48.6, 47.1, 40.4, 35.5, 31.0, 29.4, 28.1, 22.2; HRMS (ESI) m/z calculated for $C_{24}H_{30}N_4NaO_6$ $[M+Na]^+$ = 493.2058, found 493.2056.

tert-butyl (2-((2-(2,6-dioxopiperidin-3-yl)-1,3-dioxoisoindolin-4-yl)amino)ethyl)carbamate (9c)

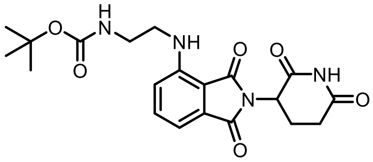

9c

Compound **9c** was isolated as a yellow solid, (0.238 g, 92%); ^{1}H NMR (400 MHz, DMSO-d_6) δ 11.10 (s, 1H), 7.56 (dd, J = 8.6, 7.1 Hz, 1H), 7.13 (d, J = 8.6 Hz, 1H), 7.02 (d, J = 7.2 Hz, 2H), 6.70 (t, J = 6.2 Hz, 1H), 5.05 (dd, J = 12.8, 5.4 Hz, 1H), 3.36 (q, J = 6.3 Hz, 2H), 3.12 (q, J = 6.1 Hz, 2H), 2.94 – 2.81 (m, 1H), 2.63 – 2.50 (m, 2H), 2.61 – 1.95(m, 1H), 1.35 (s, 9H); ^{13}C NMR (101 MHz, DMSO-d_6) δ 172.9, 170.2, 168.8, 167.4, 156.0, 146.5, 136.2, 132.2, 117.1, 110.6, 109.2, 77.9, 48.6, 41.6, 31.0, 28.2, 22.2; HRMS (ESI) m/z calculated for $C_{20}H_{24}N_4NaO_6$ $[M+Na]^+$ = 493.1588, found 493.1592.

4-(benzylamino)-2-(2,6-dioxopiperidin-3-yl)isoindoline-1,3-dione (9d)

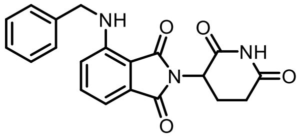

9d

Compound **9d** was isolated as a yellow solid, (0.203 g, 93%); ^{1}H NMR (400 MHz, DMSO-d_6) δ 11.08 (s, 1H), 7.49 – 7.40 (m, 1H), 7.33 – 7.27 (m, 4H), 7.23 – 7.11 (m, 2H), 6.97 (d, J = 7.1 Hz, 1H), 6.90 (d, J = 8.6 Hz, 1H), 5.01 (dd, J = 12.7, 5.4 Hz, 1H), 4.49 (d, J = 6.2 Hz, 2H), 2.88 – 2.75 (m, 1H), 2.61 – 2.51 (m, 2H), 2.04 – 1.96 (m, 1H); ^{13}C NMR (101 MHz, DMSO-d_6) δ 173.2, 170.4, 169.0, 167.6, 146.3, 139.1, 136.4, 132.4, 128.8, 127.3, 127.2, 117.9, 111.1, 109.7, 48.8, 45.6, 31.2, 22.4; HRMS (ESI) m/z calculated for $C_{20}H_{17}N_3NaO_4$ $[M+Na]^+$ = 386.1111, found 386.1116.

tert-butyl 2-((2-(2,6-dioxopiperidin-3-yl)-1,3-dioxoisoindolin-4-yl)amino)acetate (9e)

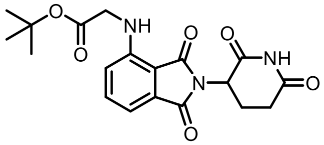

9e

Compound **9e** was isolated as a yellow solid, (0.209 g, 90%); ^{1}H NMR (400 MHz, DMSO-d_6) δ 11.10 (s, 1H), 7.58 (dd, J = 8.5, 7.1 Hz, 1H), 7.08 (d, J = 7.0 Hz, 1H), 6.97 (d, J = 8.6 Hz, 1H), 6.84 (t, J = 6.1 Hz, 1H), 5.07 (dd, J = 12.9, 5.4 Hz, 1H), 4.09 (d, J = 6.0 Hz, 2H), 2.89 (ddd, J = 17.5, 13.9, 5.4 Hz, 1H), 2.64 – 2.52 (m, 2H), 2.08 – 2.00 (m, 1H), 1.43 (s, 9H); ^{13}C NMR (101 MHz, DMSO-d_6) δ 172.8, 170.0, 169.2, 168.7, 167.3, 145.9, 136.1, 132.0, 117.7, 111.2, 109.7, 81.3, 48.6, 44.4, 31.0, 27.7, 22.1; HRMS (ESI) m/z calculated for $C_{19}H_{21}N_3NaO_6$ $[M+Na]^+$ = 410.1323, found 410.1318.

2-(2,6-dioxopiperidin-3-yl)-4-((2-hydroxyethyl)amino)isoindoline-1,3-dione (9f)

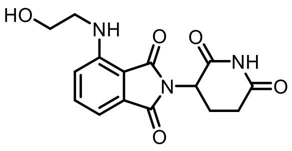

9f

Compound **9f** was isolated as a yellow solid (0.173 g, 91%); ^{1}H NMR (400 MHz, DMSO-d_6) δ 11.04 (s, 1H), 7.57 – 7.49 (m, 1H), 7.08 (d, *J* = 8.6 Hz, 1H), 6.99 (d, *J* = 7.0 Hz, 1H), 6.59 (t, *J* = 5.8 Hz, 1H), 5.01 (dd, *J* = 12.9, 5.4 Hz, 1H), 4.89 (t, *J* = 5.2 Hz, 1H), 3.55 (q, *J* = 5.3 Hz, 2H), 3.31 (q, *J* = 5.8 Hz, 2H), 2.84 (ddd, *J* = 17.5, 14.1, 5.4 Hz, 1H), 2.59 – 2.51 (m, 2H), 2.02 – 1.94 (m, 1H); ^{13}C NMR (101 MHz, DMSO-d_6) δ 172.9, 170.1, 169.0, 167.3, 146.6, 136.2, 132.1, 117.5, 110.5, 109.2, 59.4, 48.5, 44.3, 31.0, 22.1; HRMS (ESI) m/z calculated for $C_{15}H_{15}N_3NaO_5$ $[M+Na]^+$ = 340.0904, found 340.0907.

2-(2,6-dioxopiperidin-3-yl)-4-((2-(2-(2-(2-hydroxyethoxy)ethoxy)ethoxy)ethyl)amino)isoindoline-1,3-dione (9g)

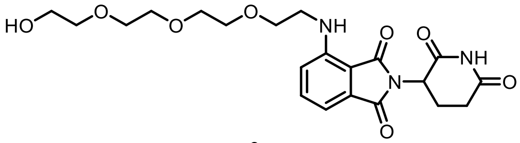

9g

Compound **9g** was isolated as a orange solid, (0.256 g, 95%); ^{1}H NMR (400 MHz, DMSO-d_6) δ 11.09 (s, 1H), 7.58 (dd, *J* = 8.5, 7.1 Hz, 1H), 7.14 (d, *J* = 8.6 Hz, 1H), 7.04 (d, *J* = 7.0 Hz, 1H), 6.60 (t, *J* = 5.8 Hz, 1H), 5.05 (dd, *J* = 12.9, 5.4 Hz, 1H), 4.56 (t, *J* = 5.5 Hz, 1H), 3.62 (t, *J* = 5.4 Hz, 2H), 3.58 – 3.51 (m, 4H), 3.53 – 3.48 (m, 4H), 3.48 – 3.43 (m, 4H), 3.39 (t, *J* = 5.4 Hz, 2H), 2.88 (ddd, *J* = 17.5, 14.1, 5.4 Hz, 1H), 2.64 – 2.50 (m, 2H), 2.07 – 1.97 (m, 1H); ^{13}C NMR (101 MHz, DMSO-d_6) δ 172.8, 170.1, 168.9, 167.3, 146.4, 136.2, 132.1, 117.4, 110.6, 109.2, 72.3, 69.8, 69.7, 68.8, 60.2, 48.5, 41.7, 30.9, 22.1; HRMS (ESI) m/z calculated for $C_{21}H_{27}N_3NaO_8$ $[M+Na]^+$ = 472.1690, found 427.1695.

tert-butyl(2-(2-(2-((2-(2,6-dioxopiperidin-3-yl)-1,3-dioxoisoindolin-4-yl)amino)ethoxy)ethoxy)ethyl) carbamate (9h)

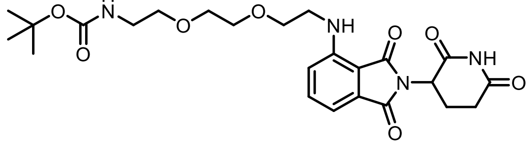

9h

Compound **9h** was isolated as a yellow solid, (0.300 g, 99%); ^{1}H NMR (400 MHz, DMSO-d_6) δ 11.09 (s, 1H), 7.56 (dd, *J* = 8.6, 7.1 Hz, 1H), 7.14 (d, *J* = 8.6 Hz, 1H), 7.04 (d, *J* = 7.0 Hz, 1H), 6.77 – 6.67 (m, 1H), 6.60 (t, *J* = 5.9 Hz, 1H), 5.05 (dd, *J* = 12.9, 5.4 Hz, 1H), 3.62 (t, *J* = 5.5 Hz, 2H), 3.57 – 3.49 (m, 4H), 3.47 (q, *J* = 5.7 Hz, 2H), 3.38 (t, *J* = 6.1 Hz, 2H), 3.05 (q, *J* = 6.0 Hz, 2H), 2.88 (ddd, *J* = 17.4, 14.0, 5.5 Hz, 1H), 2.64 – 2.49 (m, 2H), 2.07 – 1.97 (m, 1H), 1.36 (s, 9H); ^{13}C NMR (101 MHz, DMSO-d_6) δ 172.8, 170.0, 168.9, 167.3, 155.5, 146.4, 136.2, 132.1, 117.4, 110.6, 109.2, 77.5, 69.7, 69.5, 69.2, 68.9, 48.5, 41.7, 31.0, 28.2, 22.1; HRMS (ESI) m/z calculated for $C_{24}H_{32}N_4NaO_8$ $[M+Na]^+$ = 527.2112, found

527.2116.

tert-butyl (3-((2-(2,6-dioxopiperidin-3-yl)-1,3-dioxoisoindolin-4-yl)amino)propyl)carbamate (9i)

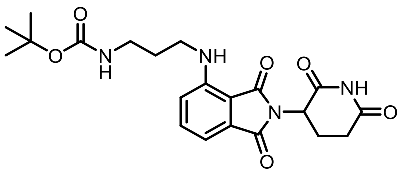

9i

Compound **9i** was isolated as a yellow solid, (0.248 g, 96%);^{1}H NMR (400 MHz, DMSO-d_6) δ 11.10 (s, 1H), 7.57 (dd, J = 8.6, 7.0 Hz, 1H), 7.07 (d, J = 8.6 Hz, 1H), 7.01 (d, J = 7.0 Hz, 1H), 6.91 (t, J = 5.9 Hz, 1H), 6.65 (t, J = 6.1 Hz, 1H), 5.04 (dd, J = 12.7, 5.4 Hz, 1H), 3.30 (q, J = 6.6 Hz, 2H), 2.99 (q, J = 6.4 Hz, 2H), 2.87 (ddd, J = 16.8, 13.7, 5.3 Hz, 1H), 2.62 – 2.52 (m, 2H),2.09 – 1.95 (m, 1H), 1.65 (t, J = 6.7 Hz, 2H), 1.36 (s, 9H); ^{13}C NMR (101 MHz, DMSO-d_6) δ 173.0, 170.2, 168.9, 167.5, 155.9, 146.4, 136.4, 132.3, 117.2, 110.5, 109.2, 77.7, 48.6, 37.4, 31.1, 29.0, 28.3, 22.3. HRMS (ESI): m/z calculated for $C_{21}H_{26}N_4NaO_6$ $[M+Na]^+$ = 453.1745, found 453.1752.

tert-butyl (6-((2-(2,6-dioxopiperidin-3-yl)-1,3-dioxoisoindolin-4-yl)amino)hexyl)carbamate (9j)

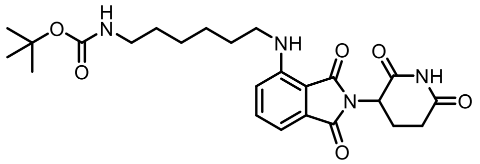

9j

Compound **9j** was isolated as a yellow solid, (0.263 g, 93%); ^{1}H NMR (400 MHz, DMSO-d6) δ 11.01 (s, 1H), 7.57 (t, J = 7.8 Hz, 1H), 7.07 (d, J = 8.6 Hz, 1H), 7.01 (d, J = 7.1 Hz, 1H), 6.75 (t, J = 5.8 Hz, 1H), 6.51 (t, J = 5.9 Hz, 1H), 5.04 (dd, J = 12.8, 5.4 Hz, 1H), 3.27 (q, J = 6.8 Hz, 2H), 2.93 – 2.86 (m, 2H), 2.86 – 2.81 (m, 1H), 2.64 – 2.51 (m, 2H), 2.06 – 1.98 (m, 1H), 1.55 (t, J = 7.2 Hz, 2H), 1.35 (s, 9H), 1.32 – 1.20 (m, 6H); ^{13}C NMR (101 MHz, DMSO-d_6) δ 172.9, 170.2, 169.0, 167.4, 155.7, 146.5, 136.4, 132.2, 117.2, 110.4, 109.0, 77.4, 48.6, 41.8, 31.0, 29.5, 28.7, 28.3, 26.0. HRMS (ESI): m/z calculated for $C_{24}H_{32}N_4NaO_6$ $[M+Na]^+$ = 495.2214, found 495.2217.

tert-butyl (2-((2-(2,6-dioxopiperidin-3-yl)-1,3-dioxoisoindolin-4-yl)(methyl)amino)ethyl)carbamate (9k)

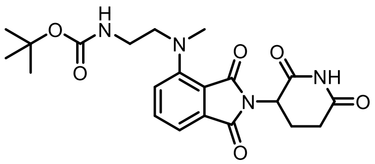

9k

Compound **9k** was isolated as a yellow solid, (0.242 g, 94%); ^{1}H NMR (400 MHz, DMSO-d_6) δ 11.06 (s, 1H), 7.60 (dd, J = 8.6, 7.0 Hz, 1H), 7.22 (dd, J = 14.2, 7.8 Hz, 2H), 6.71 (t, J = 5.9 Hz, 1H), 5.08 (dd, J = 12.8, 5.4 Hz, 1H), 3.58 (s, 2H), 3.14 (q, J = 6.3 Hz, 2H), 3.02 (s, 3H), 2.89 (ddd, J = 17.3, 13.9, 5.4 Hz, 1H), 2.64 – 2.53 (m, 2H), 2.02 – 1.94 (m, 1H), 1.28 (s, 9H); ^{13}C NMR (101 MHz, DMSO-d6) δ = 172.8,

170.0, 167.1, 166.3, 155.4, 149.4, 135.0, 133.9, 123.1, 113.2, 112.8, 77.6, 54.1, 48.7,39.9, 37.6, 30.9, 28.1, 22.1; HRMS (ESI) m/z calculated for $C_{21}H_{26}N_4NaO_6$ $[M+Na]^+$ = 453.1745, found 453.1756.

4-(benzyl(methyl)amino)-2-(2,6-dioxopiperidin-3-yl)isoindoline-1,3-dione (9l)

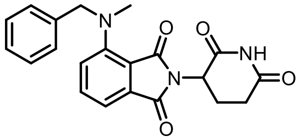

9l

Compound **9l** was isolated as a yellow solid, (0.224 g, 99 %); ^{1}H NMR (400 MHz, DMSO-*d*$_6$) δ 11.08 (s, 1H), 7.63 (dd, *J* = 8.3, 7.3 Hz, 1H), 7.35 – 7.30 (m, 2H), 7.29 – 7.21 (m, 5H), 5.10 (dd, *J* = 12.9, 5.4 Hz, 1H), 4.70 (s, 2H), 2.94 (s, 3H), 2.91 – 2.80 (m, 1H), 2.63 – 2.53 (m, 2H), 2.08 – 1.98 (m, 1H); ^{13}C NMR (101 MHz, DMSO-*d*$_6$) δ 172.8, 170.0, 167.1, 166.5, 149.4, 137.8, 135.4, 133.9, 128.4, 127.6, 127.1, 123.6, 114.1, 113.6, 58.4, 48.8, 30.9, 22.1; HRMS (ESI) m/z calculated for $C_{21}H_{19}N_3NaO_4$ $[M+Na]^+$ = 400.1268, found 400.1271.

2-(2,6-dioxopiperidin-3-yl)-4-((2-hydroxyethyl)(methyl)amino)isoindoline-1,3-dione (9m)

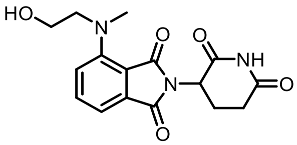

9m

Compound **9m** was isolated as a yellow solid, (0.185 g, 93%); ^{1}H NMR (400 MHz, DMSO-*d*$_6$) δ 11.07 (s, 1H), 7.60 (dd, *J* = 8.6, 6.9 Hz, 1H), 7.29 (d, *J* = 8.6 Hz, 1H), 7.20 (d, *J* = 7.0 Hz, 1H), 5.07 (dd, *J* = 12.8, 5.4 Hz, 1H), 4.64 (t, *J* = 5.1 Hz, 1H), 3.63 (t, *J* = 5.2 Hz, 2H), 3.58 (q, *J* = 5.6 Hz, 2H), 3.06 (s, 3H), 2.88 (ddd, *J* = 17.4, 14.1, 5.5 Hz, 1H), 2.63 – 2.51 (m, 2H), 2.05 – 1.96 (m, 1H); ^{13}C NMR (101 MHz, DMSO-*d*$_6$) δ 173.3, 170.5, 167.6, 166.9, 150.1, 135.5, 134.4, 123.8, 113.4, 113.2, 59.2, 57.7, 49.2, 31.4, 22.6. HRMS (ESI) m/z calculated for $C_{16}H_{17}N_3NaO_5$ $[M+Na]^+$ = 354.1060, found 354.1062.

2-(2,6-dioxopiperidin-3-yl)-4-(methyl(prop-2-yn-1-yl)amino)isoindoline-1,3-dione (9n)

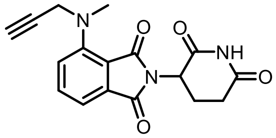

9n

Compound **9n** was isolated as a yellow solid, (0.181 g, 93%); ^{1}H NMR (400 MHz, DMSO-*d*$_6$) δ 11.09 (s, 1H), 7.71 (t, *J* = 7.8 Hz, 1H), 7.38 (s, 1H), 7.36 (s, 1H), 5.10 (dd, *J* = 12.9, 5.4 Hz, 1H), 4.32 (d, *J* = 2.4 Hz, 2H), 3.24 – 3.18 (m, 1H), 3.01 (s, 3H), 2.95 – 2.87 (m, 1H), 2.64 – 2.53 (m, 2H), 2.08 – 2.01 (m, 1H); ^{13}C NMR (101 MHz, DMSO-*d*$_6$) δ 172.8, 170.0, 167.0, 166.4, 148.5, 135.6, 133.7, 124.3, 115.9, 114.8, 79.4, 75.8, 48.9, 44.2,40.1, 30.9, 22.1; HRMS (ESI) m/z calculated for $C_{17}H_{15}N_3NaO_4$ $[M+Na]^+$ = 348.0955, found 348.0952.

tert-butyl ((1-(2-(2,6-dioxopiperidin-3-yl)-1,3-dioxoisoindolin-4-yl)piperidin-4-

yl)methyl)carbamate (9o)

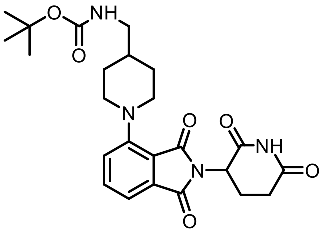

9o

Compound **9o** was isolated as a yellow solid, (0.254 g, 90%); ^{1}H NMR (400 MHz, DMSO-d_6) δ 11.08 (s, 1H), 7.67 (t, J = 7.8 Hz, 1H), 7.29 (d, J = 8.6 Hz, 1H), 7.20 (d, J = 7.0 Hz, 1H), 6.92 – 6.88 (m, 1H), 5.08 (dd, J = 12.9, 5.4 Hz, 1H), 3.67 (d, J = 11.8 Hz, 2H), 2.98 – 2.75 (m, 5H), 2.63 – 2.51 (m, 2H), 2.08 – 1.96 (m, 1H), 1.72 (d, J = 12.5 Hz, 2H), 1.54 (s, 1H), 1.38 (s, 9H), 1.34 – 1.26 (m, 2H); ^{13}C NMR (101 MHz, DMSO-d_6) δ 172.8, 170.1, 167.1, 166.3, 155.8, 150.2, 135.7, 133.7, 124.0, 116.4, 114.4, 77.4, 50.9, 48.8, 45.4, 35.8, 31.0, 29.6, 28.3, 22.1; HRMS (ESI) m/z calculated for $C_{24}H_{30}N_4NaO_6$ $[M+Na]^+$ = 493.2058, found 493.2066.

tert-butyl 4-(2-(2,6-dioxopiperidin-3-yl)-1,3-dioxoisoindolin-4-yl)piperazine-1-carboxylate (9p)

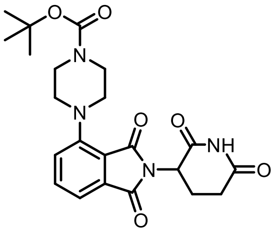

9p

Compound **9p** was isolated as a yellow solid, (0.249 g, 94%); ^{1}H NMR (400 MHz, DMSO-d_6) δ 11.09 (s, 1H), 7.72 (dd, J = 8.4, 7.1 Hz, 1H), 7.39 (d, J = 7.1 Hz, 1H), 7.35 (d, J = 8.4 Hz, 1H), 5.10 (dd, J = 12.9, 5.4 Hz, 1H), 3.54 – 3.46 (m, 4H), 3.28 – 3.21 (m, 4H), 2.95 – 2.81 (m, 1H), 2.63 – 2.53 (m, 2H), 2.07 – 1.98 (m, 1H), 1.42 (s, 9H); ^{13}C NMR (101 MHz, DMSO-d_6) δ 172.8, 169.9, 167.0, 166.3, 153.8, 149.5, 135.9, 133.6, 123.9, 117.0, 115.2, 79.0, 50.4, 48.8, 30.9, 28.0, 22.0; HRMS (ESI) m/z calculated for $C_{22}H_{26}N_4NaO_6$ $[M+Na]^+$ = 465.1745, found 465.1752.

tert-butyl 1-(2-(2,6-dioxopiperidin-3-yl)-1,3-dioxoisoindolin-4-yl)piperidine-4-carboxylate (9q)

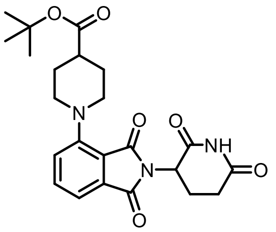

9q

Compound **9q** was isolated as a yellow solid, (0.246 g, 93%); ^{1}H NMR (400 MHz, DMSO-d_6) δ 11.08 (s, 1H), 7.68 (t, J = 7.8 Hz, 1H), 7.34 (s, 1H), 7.32 (d, J = 1.6 Hz, 1H), 5.09 (dd, J = 12.9, 5.4 Hz, 1H), 3.66 – 3.58 (m, 2H), 2.95 (t, J = 10.8 Hz, 2H), 2.90 – 2.81 (m, 1H), 2.63 – 2.52 (m, 2H), 2.46 – 2.38 (m, 1H), 2.06 – 1.98 (m, 1H), 1.95 – 1.87 (m, 2H), 1.73 (q, J = 11.8 Hz, 2H), 1.42 (s, 9H); ^{13}C NMR (101 MHz,

DMSO-d_6) δ 173.5, 172.8, 170.0, 167.0, 166.3, 149.9, 135.8, 133.6, 123.9, 116.6, 114.6, 79.6, 50.2, 48.7, 40.6, 30.9, 28.0, 27.7, 22.0; HRMS (ESI) m/z calculated for $C_{24}H_{31}N_3NaO_6$ $[M+Na]^+$ = 480.2105, found 480.2108.

tert-butyl 9-(2-(2,6-dioxopiperidin-3-yl)-1,3-dioxoisoindolin-4-yl)-3,9-diazaspiro[5.5]undecane-3-carboxylate (9r)

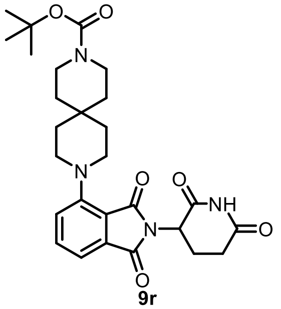

9r

Compound **9r** was isolated as a yellow solid, (0.288 g, 94%); ^{1}H NMR (400 MHz, DMSO-d_6) δ 11.08 (s, 1H), 7.67 (dd, J = 8.5, 7.1 Hz, 1H), 7.32 (t, J = 7.6 Hz, 2H), 5.08 (dd, J = 12.9, 5.4 Hz, 1H), 3.33 – 3.29 (m, 4H), 3.28 – 3.24 (m, 4H), 2.87 (ddd, J = 17.4, 14.1, 5.5 Hz, 1H), 2.63 – 2.51 (m, 2H), 2.07 – 1.96 (m, 1H), 1.68 – 1.57 (m, 4H), 1.44 – 1.40 (m, 4H), 1.39 (s, 9H). ^{13}C NMR (101 MHz, DMSO-d_6) δ 172.8, 170.0, 167.1, 166.3, 153.9, 150.0, 135.7, 133.7, 123.8, 116.1, 114.3, 78.4, 48.7, 46.4, 34.9, 30.9, 29.2, 28.1, 22.1; HRMS (ESI): m/z calcd for $C_{24}H_{34}N_4NaO_6$ $[M+Na]^+$ = 533.2371; found 533.2378.

tert-butyl (1-(2-(2,6-dioxopiperidin-3-yl)-1,3-dioxoisoindolin-4-yl)azetidin-3-yl)(methyl)carbamate (9s)

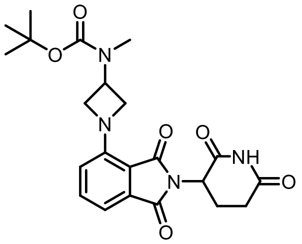

9s

Compound **9s** was isolated as a orange solid, (0.239 g, 90%); ^{1}H NMR (400 MHz, DMSO-d_6) δ 11.07 (s, 1H), 7.58 (t, J = 7.8 Hz, 1H), 7.14 (d, J = 7.0 Hz, 1H), 6.82 (d, J = 8.5 Hz, 1H), 5.06 (dd, J = 12.9, 5.4 Hz, 1H), 4.78 (m, 1H), 4.90 – 4.64 (m, 2H), 4.41 (t, J = 8.7 Hz, 2H), 4.17 (dd, J = 9.4, 5.9 Hz, 3H), 2.92 – 2.82 (m, 1H), 2.62 – 2.54 (m, 2H), 2.04 – 1.96 (m, 1H), 1.40 (s, 9H); ^{13}C NMR (101 MHz, DMSO-d_6) δ 172.8, 170.0, 167.2, 166.5, 154.6, 147.4, 134.9, 133.2, 120.2, 112.0, 110.6, 79.3, 48.6, 30.9, 28.0, 22.1; HRMS (ESI): m/z calcd for $C_{22}H_{26}N_4NaO_6$ $[M+Na]^+$ = 465.1745; found 465.1756

2-((S)-4-(4-chlorophenyl)-2,3,9-trimethyl-6H-thieno[3,2-f][1,2,4]triazolo[4,3-a][1,4]diazepin-6-yl)-N-(2-(2-(2-((2-(2,6-dioxopiperidin-3-yl)-1,3-dioxoisoindolin-4-yl)amino)ethoxy)ethoxy)ethyl)acetamide (10a)

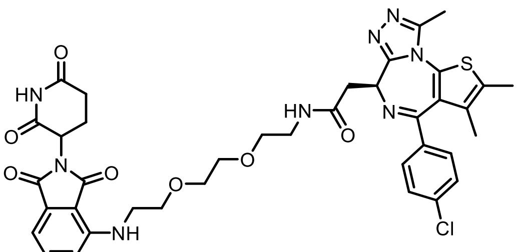

10a

This procedure was used for the synthesis of compounds **10a–10d**. The corresponding Boc-protected pomalidomide derivative was dissolved in dichloromethane (DCM, 0.15 M). Trifluoroacetic acid (TFA, 3 mL per mmol of substrate) was added. The reaction mixture was stirred at room temperature for 2 h. After completion, the mixture was concentrated directly under reduced pressure to remove TFA and volatile byproducts, affording the deprotected amine as a solid or oil, which was used directly in the next step without further purification. To a solution of the deprotected amine (0.2 mmol, 1.0 equiv.) and the carboxylic-acid-functionalized POI ligand (1.10 equiv.) in anhydrous DMF (1.0 mL, 0.2 M) were added TCFH (1.50 equiv.) and N-methylimidazole (NMI, 3.00 equiv.). The reaction mixture was stirred at room temperature for 8 h. After concentration under reduced pressure, the crude product was purified by silica gel column chromatography (CH_2Cl_2/MeOH) and then dried in a vacuum oven for 8 h to afford the final PROTAC product.

Compound **10a** was isolated as a yellow solid, (0.068 g, 43%); ^{1}H NMR (400 MHz, DMSO-*d*6) δ 8.32 (t, *J* = 5.7 Hz, 1H), 7.55 (t, *J* = 7.8 Hz, 1H), 7.48 – 7.37 (m, 4H), 7.11 (d, *J* = 8.6 Hz, 1H), 7.00 (d, *J* = 7.0 Hz, 1H), 6.59 (t, *J* = 5.8 Hz, 1H), 5.04 (dd, *J* = 12.9, 5.4 Hz, 1H), 4.54 – 4.46 (m, 1H), 3.61 (t, *J* = 5.4 Hz, 2H), 3.58 – 3.53 (m, 4H), 3.47 – 3.42 (m, 4H), 3.32 – 3.18 (m, 4H), 2.91 – 2.82 (m, 1H), 2.57 (s, 3H), 2.56 – 2.50 (m, 2H), 2.37 (s, 3H), 2.08 – 1.96 (m, 1H), 1.59 (s, 3H); ^{13}C NMR (101 MHz, DMSO-d_6) δ 173.0, 170.2, 169.9, 169.0, 167.4, 163.2, 155.2, 150.0, 146.5, 136.8, 136.3, 135.3, 132.3, 132.2, 130.9, 130.2, 129.9, 129.7, 128.5, 117.5, 110.8, 109.3, 69.8, 69.7, 69.3, 69.0, 55.0, 53.9, 48.6, 41.8, 37.6, 31.1, 22.2, 14.1, 12.7, 11.4; HRMS (ESI): m/z calculated for $C_{38}H_{39}ClN_8NaO_7S$ $[M+Na]^+$ = 809.2243, found 809.2247.

2-((R)-4-(4-chlorophenyl)-2,3,9-trimethyl-6H-thieno[3,2-f][1,2,4]triazolo[4,3-a][1,4]diazepin-6-yl)-N-((1-(2-(2,6-dioxopiperidin-3-yl)-1,3-dioxoisoindolin-4-yl)piperidin-4-yl)methyl)acetamide (10b)

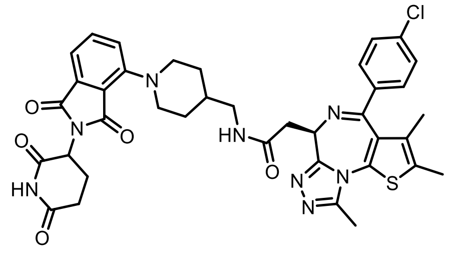

10b

Compound **10b** was isolated as a yellow solid, (0.054 g, 38%); ^{1}H NMR (400 MHz, DMSO-d_6) δ 11.09 (s, 1H), 8.31 (t, *J* = 5.8 Hz, 1H), 7.67 (t, *J* = 8.0 Hz, 1H), 7.43 (q, *J* = 8.5 Hz, 4H), 7.32 (m, 2H), 5.07 (ddd, *J* = 13.1, 5.6, 2.8 Hz, 1H), 4.52 (t, *J* = 7.1 Hz, 1H), 3.69 (d, *J* = 11.1 Hz, 2H), 3.36 – 3.25 (m, 2H), 3.24 – 3.04 (m, 3H), 2.93 – 2.80 (m, 3H), 2.59 (s, 3H), 2.56 – 2.53 (m, 1H), 2.39 (s, 3H), 2.03 – 1.98 (m,

1H), 1.84 – 1.76 (m, 2H), 1.66 (s, 1H), 1.60 (s, 3H), 1.37 (t, *J* = 12.0 Hz, 2H); ^{13}C NMR (101 MHz, DMSO-d_6) δ 172.9, 170.1, 169.8, 167.2, 166.4, 163.2, 155.2, 150.2, 150.0, 136.8, 135.9, 135.3, 133.7, 132.3, 130.9, 130.2, 129.9, 129.7, 128.5, 124.0, 116.4, 114.5, 54.9, 54.1, 48.9, 44.1, 37.8, 35.6, 32.9, 31.0, 29.8, 22.2, 14.1, 12.7, 11.4; HRMS (ESI): m/z calculated for $C_{38}H_{37}ClN_8NaO_5S$ $[M+Na]^+$ = 755.2188, found 755.2190.

((1S,4a'S,6R,7'S,9a'R)-5,5-dimethyl-8'-methylene-1',2,9'-trioxo-4a',5',6',7',8',9'-hexahydro-1'H,3'H-spiro[cyclohexane-1,4'-[7,9a]methanocyclohepta[c]pyran]-3-en-6-yl)methyl 4-(1-(4-((3-((2-(2,6-dioxopiperidin-3-yl)-1,3-dioxoisoindolin-4-yl)amino)propyl)amino)-4-oxobutanoyl)piperidin-4-yl)benzoate (10c)

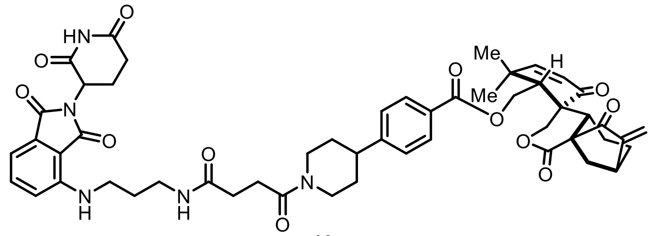

10c

Compound **10c** was isolated as a yellow solid, (0.054 g, 29%); ^{1}H NMR (400 MHz, DMSO-d_6) δ 11.10 (s, 1H), 7.93 (t, *J* = 5.8 Hz, 1H), 7.80 (d, *J* = 8.1 Hz, 2H), 7.57 (dd, *J* = 8.6, 7.1 Hz, 1H), 7.40 (d, *J* = 8.2 Hz, 2H), 7.11 (d, *J* = 8.6 Hz, 1H), 7.01 (d, *J* = 7.0 Hz, 1H), 6.84 (d, *J* = 10.2 Hz, 1H), 6.69 (t, *J* = 6.4 Hz, 1H), 5.91 – 5.83 (m, 2H), 5.58 (s, 1H), 5.05 (dd, *J* = 12.9, 5.3 Hz, 1H), 4.68 (d, *J* = 11.5 Hz, 1H), 4.64 – 4.49 (m, 4H), 4.04 – 3.96 (m, 1H), 3.17 (d, *J* = 5.0 Hz, 1H), 3.12 (q, *J* = 5.9 Hz, 3H), 3.05 – 3.00 (m, 1H), 2.88 (ddd, *J* = 16.8, 14.0, 5.2 Hz, 2H), 2.64 – 2.53 (m, 5H), 2.39 – 2.28 (m, 3H), 2.19 (dd, *J* = 14.0, 6.5 Hz, 1H), 2.04 – 1.99 (m, 2H), 1.79 (t, *J* = 14.1 Hz, 2H), 1.71 – 1.61 (m, 4H), 1.55 – 1.43 (m, 4H), 1.28 (s, 4H), 1.20 (s, 4H); ^{13}C NMR (101 MHz, DMSO-d_6) δ 202.2, 199.5, 172.8, 171.7, 170.1, 169.7, 169.0, 168.8, 167.3, 165.2, 159.5, 151.8, 150.4, 146.3, 136.2, 132.2, 129.4, 127.3, 127.1, 123.6, 119.3, 117.2, 110.3, 109.1, 69.3, 61.0, 58.0, 54.9, 50.9, 48.5, 45.2, 43.5, 41.7, 41.6, 36.2, 35.8, 34.2, 33.6, 32.8, 32.2, 31.3, 30.8, 30.5, 29.8, 29.5, 27.9, 24.5, 23.1, 22.1, 17.5, 13.9; HRMS (ESI): m/z calculated for $C_{52}H_{57}N_5NaO_{12}$ $[M+Na]^+$ = 966.3896, found 966.3892.

((1S,4a'S,6R,7'S,9a'R)-5,5-dimethyl-8'-methylene-1',2,9'-trioxo-4a',5',6',7',8',9'-hexahydro-1'H,3'H-spiro[cyclohexane-1,4'-[7,9a]methanocyclohepta[c]pyran]-3-en-6-yl)methyl 4-(1-(4-((6-((2-(2,6-dioxopiperidin-3-yl)-1,3-dioxoisoindolin-4-yl)amino)hexyl)amino)-4-oxobutanoyl)piperidin-4-yl)benzoate (10d)

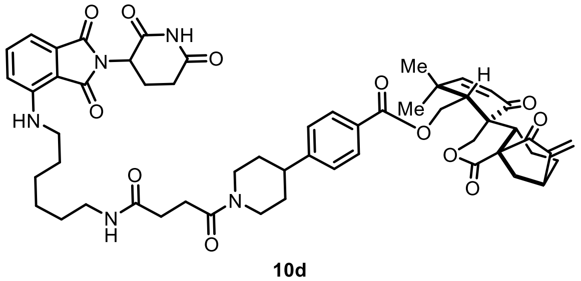

10d

Compound **10d** was isolated as a yellow solid, (0.041 g, 21%); ^{1}H NMR (400 MHz, DMSO-d_6) δ 11.10 (s, 1H), 7.81 (d, J = 8.2 Hz, 3H), 7.57 (ddd, J = 8.8, 7.1, 2.4 Hz, 1H), 7.40 (d, J = 8.3 Hz, 2H), 7.09 (dd, J = 8.6, 2.8 Hz, 1H), 7.01 (dd, J = 7.0, 2.2 Hz, 1H), 6.84 (d, J = 10.2 Hz, 1H), 6.54 (t, J = 5.9 Hz, 1H), 5.91 – 5.83 (m, 2H), 5.58 (s, 1H), 5.05 (dd, J = 12.9, 5.3 Hz, 1H), 4.68 (d, J = 11.5 Hz, 1H), 4.64 – 4.49 (m, 4H), 4.04 – 3.96 (m, 1H), 3.28 (q, J = 6.8 Hz, 3H), 3.17 (s, 2H), 3.02 (q, J = 6.5 Hz, 4H), 2.95 – 2.82 (m, 2H), 2.62 – 2.52 (m, 5H), 2.42 – 2.28 (m, 3H), 2.19 (dt, J = 14.0, 6.5 Hz, 1H), 2.13 – 2.00 (m, 2H), 1.79 (t, J = 14.1 Hz, 2H), 1.65 – 1.50 (m, 5H), 1.44 – 1.37 (m, 3H), 1.37 – 1.33 (m, 4H), 1.28 (s, 4H), 1.20 (s, 4H); ^{13}C NMR (101 MHz, DMSO-d_6) δ 202.2, 199.5, 172.8, 171.2, 170.1, 169.7, 169.0, 168.9, 167.3, 165.2, 159.5, 151.8, 150.4, 146.4, 136.3, 132.2, 129.4, 127.3, 127.1, 123.6, 119.3, 117.2, 110.3, 109.0, 69.3, 61.0, 58.0, 50.9, 48.5, 45.2, 43.5, 41.7, 41.6, 38.4, 36.2, 34.2, 32.8, 31.3, 31.0, 30.8, 30.5, 29.5, 29.1, 29.0, 28.6, 27.9, 26.1, 26.0, 23.1, 22.1, 22.1, 17.5, 13.9; HRMS (ESI): m/z calculated for $C_{55}H_{63}N_5NaO_{12}$ $[M+Na]^+$ = 1008.4365, found 1008.4369.

7. NMR spectra of compounds

^{1}H NMR (400 MHz, $CDCl_3$) of compound 2

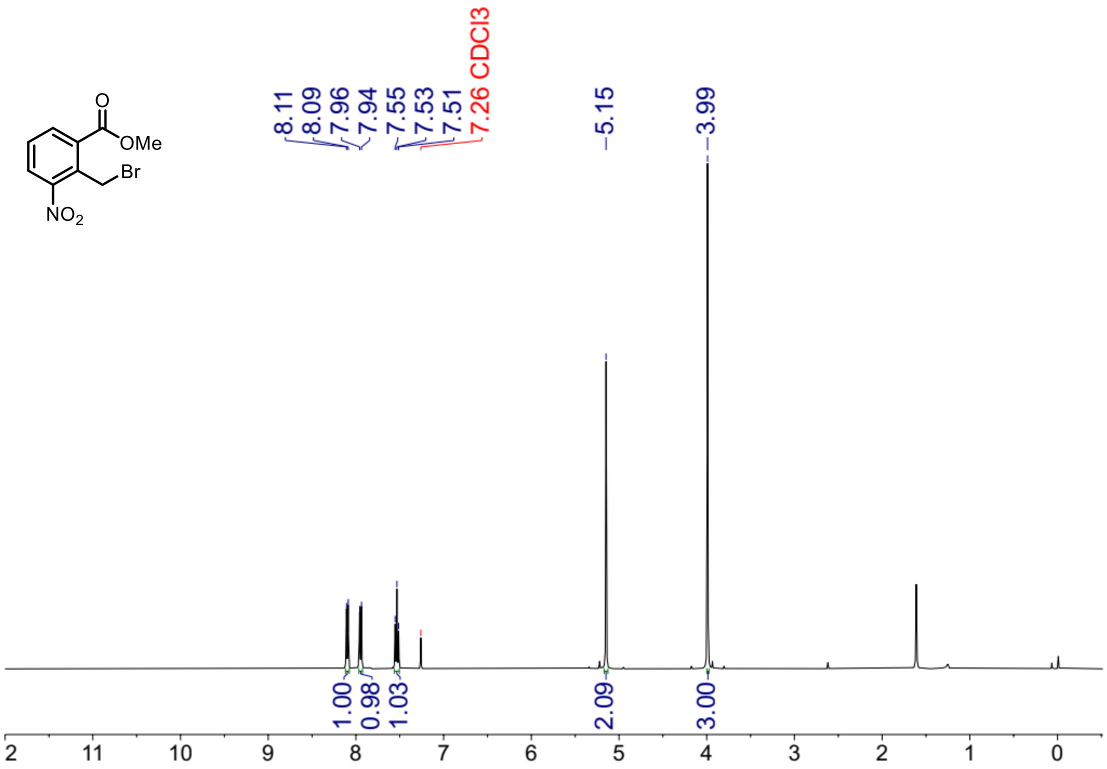

^{13}C NMR (101 MHz, $CDCl_3$) of compound 2

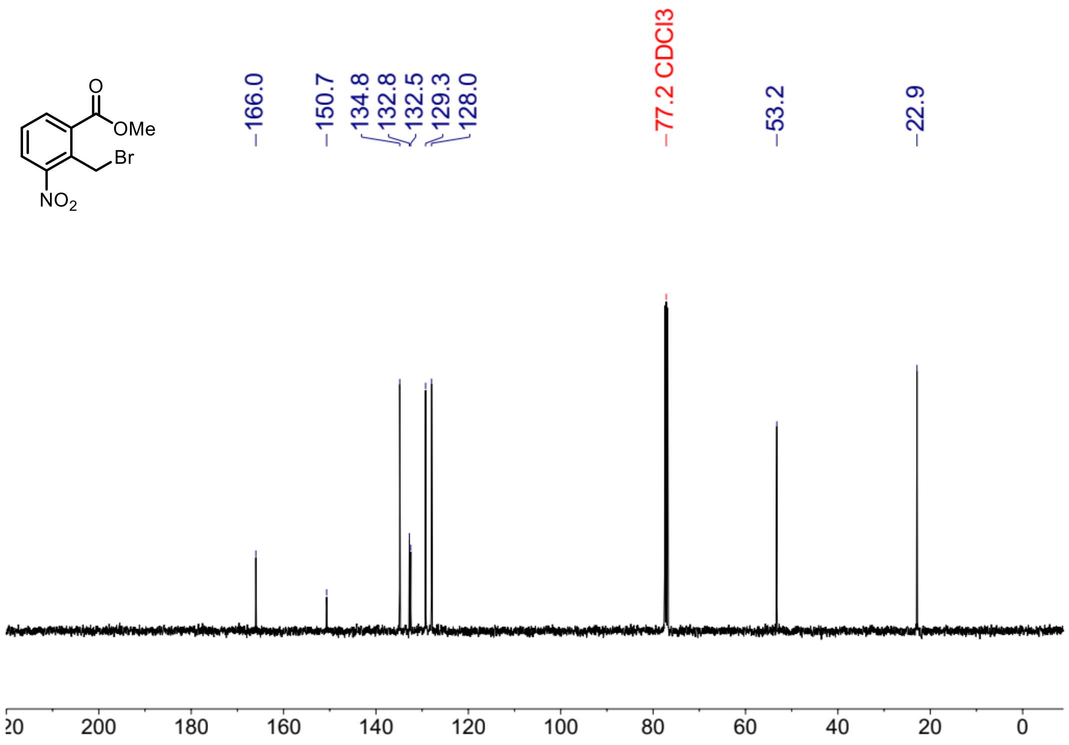

^{1}H NMR (400 MHz, DMSO-d6) of compound 4

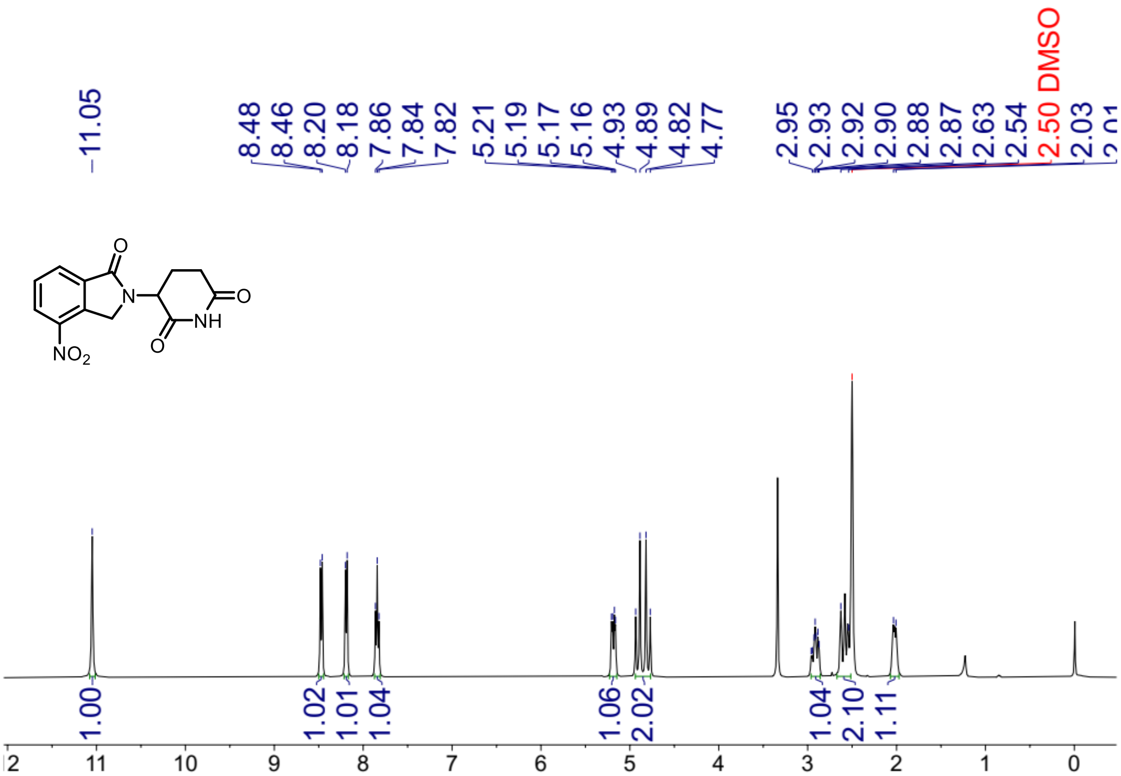

^{13}C NMR (101 MHz, DMSO-d6) of compound 4

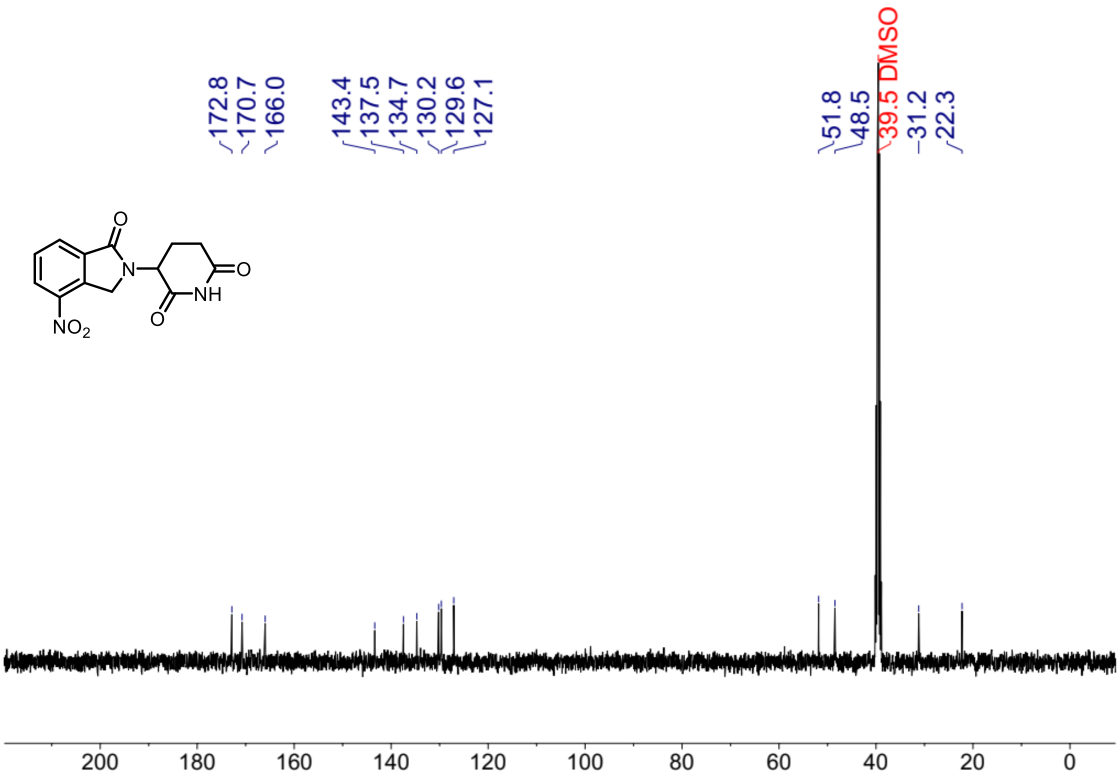

^{1}H NMR (400 MHz, DMSO-d_6) of compound 5a

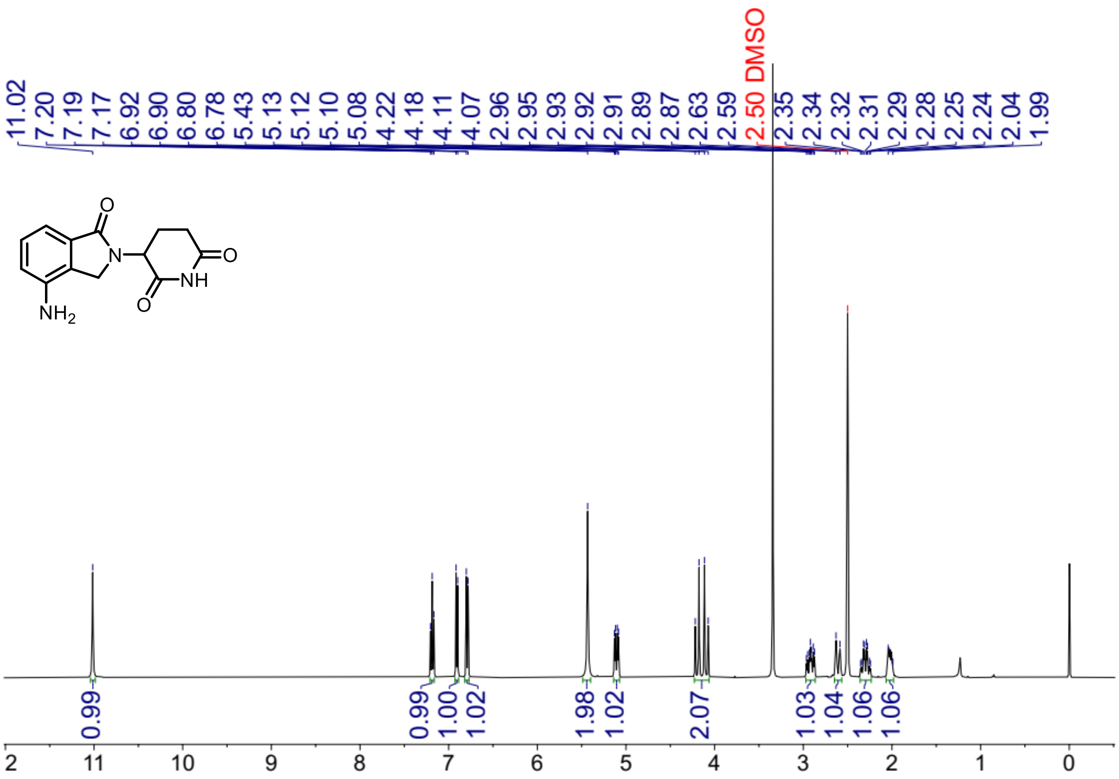

^{13}C NMR (101 MHz, DMSO-d_6) of compound 5a

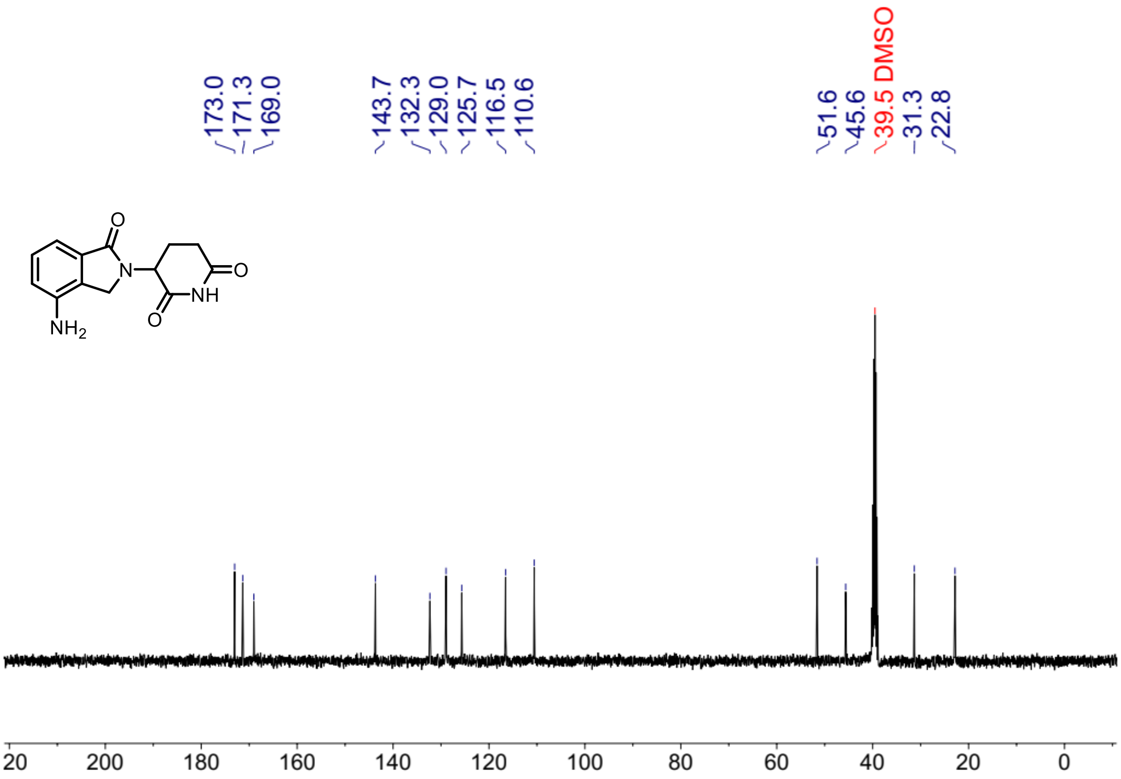

^{1}H NMR (400 MHz, $CDCl_3$) of methyl 4-methyl-5-nitro-[1,1'-biphenyl]-3-carboxylate

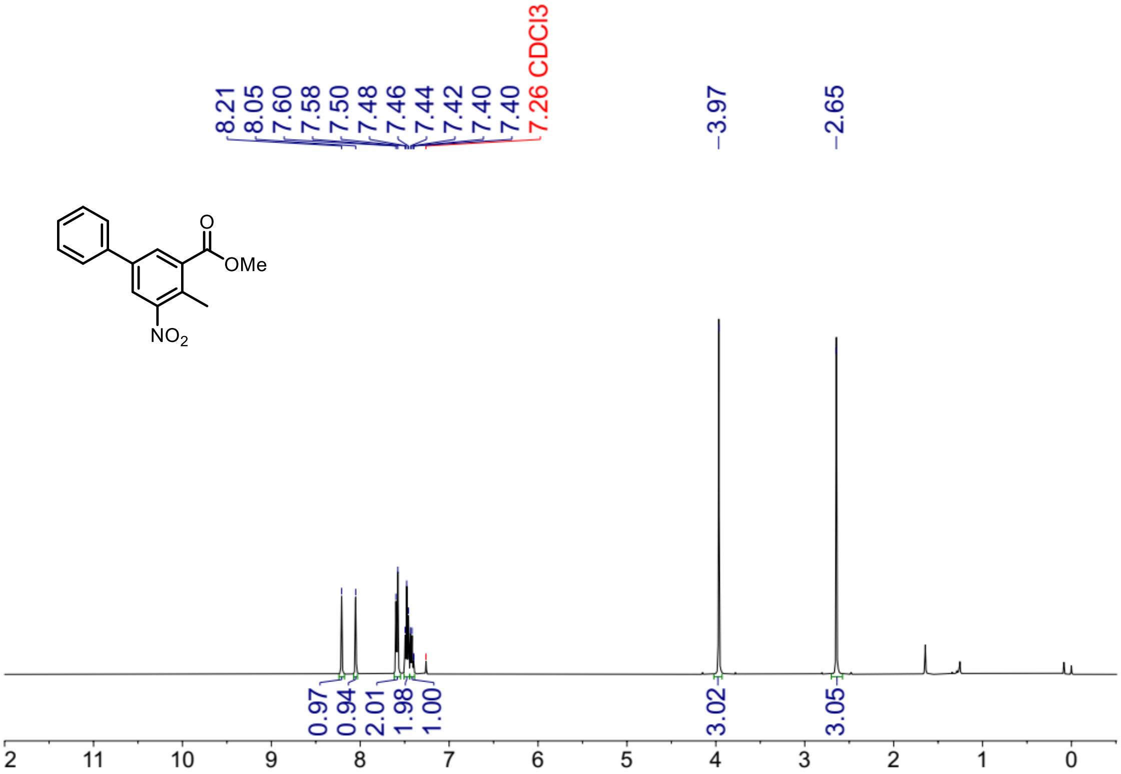

^{13}C NMR (101 MHz, $CDCl_3$) of methyl 4-methyl-5-nitro-[1,1'-biphenyl]-3-carboxylate

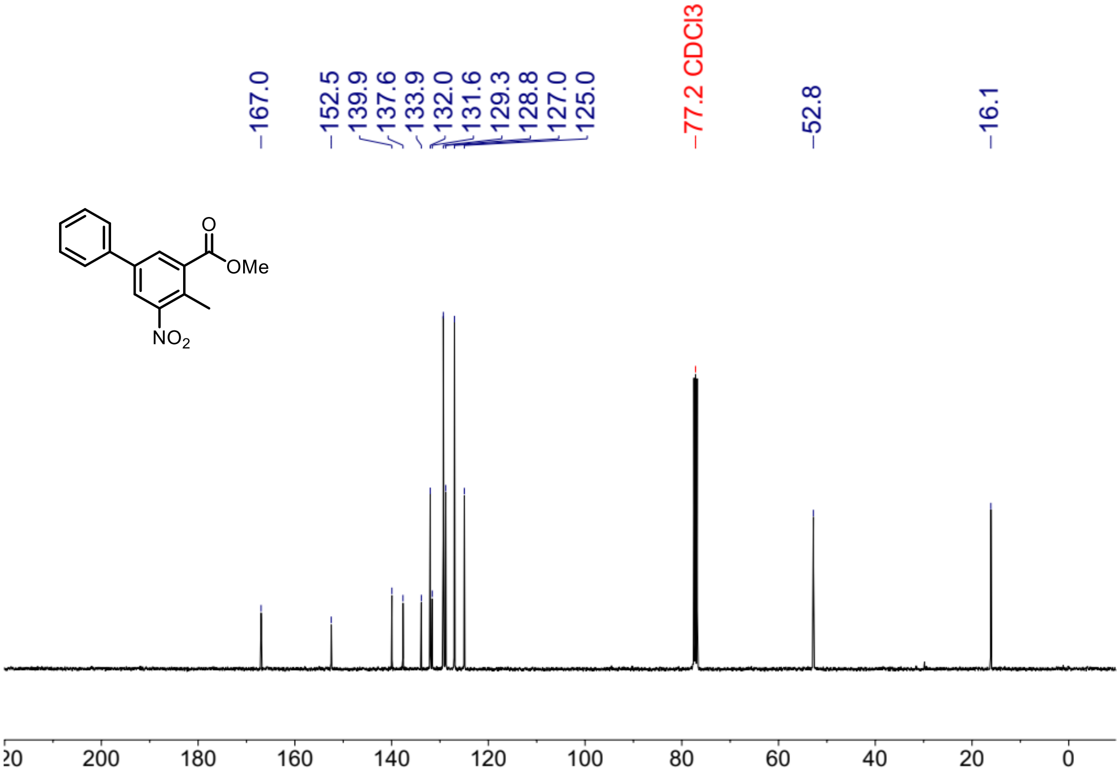

^{1}H NMR (400 MHz, $CDCl_3$) of methyl 4'-(tert-butyl)-4-methyl-5-nitro-[1,1'-biphenyl]-3-carboxylate

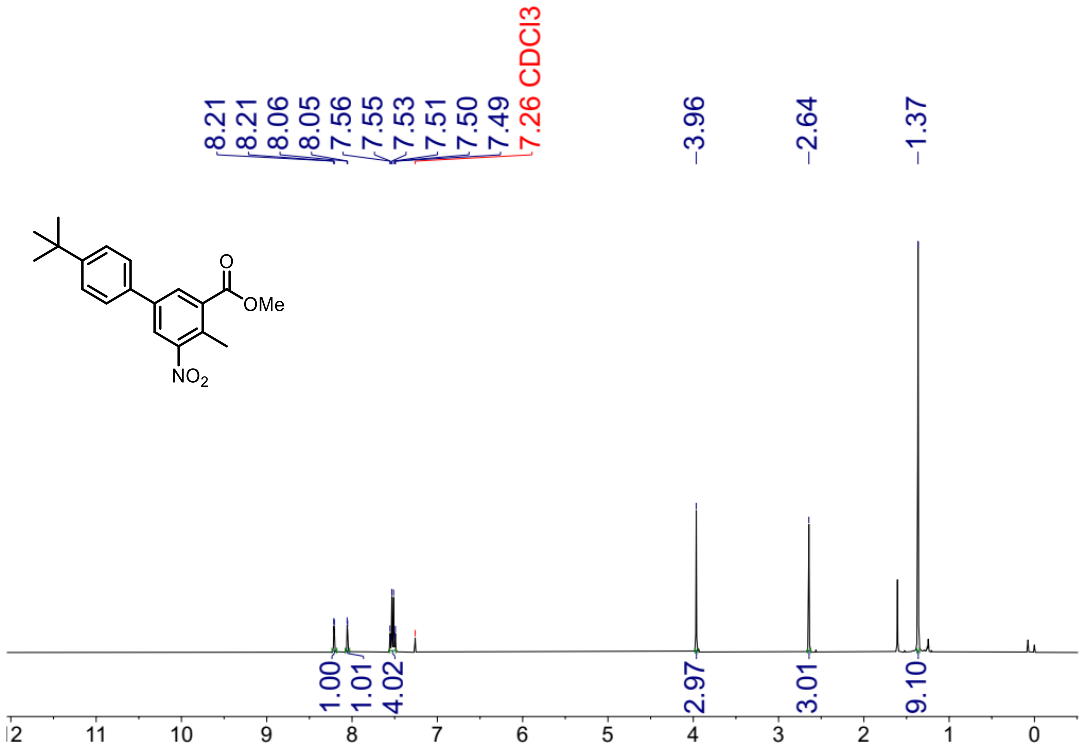

^{13}C NMR (101 MHz, $CDCl_3$) of methyl 4'-(tert-butyl)-4-methyl-5-nitro-[1,1'-biphenyl]-3-carboxylate

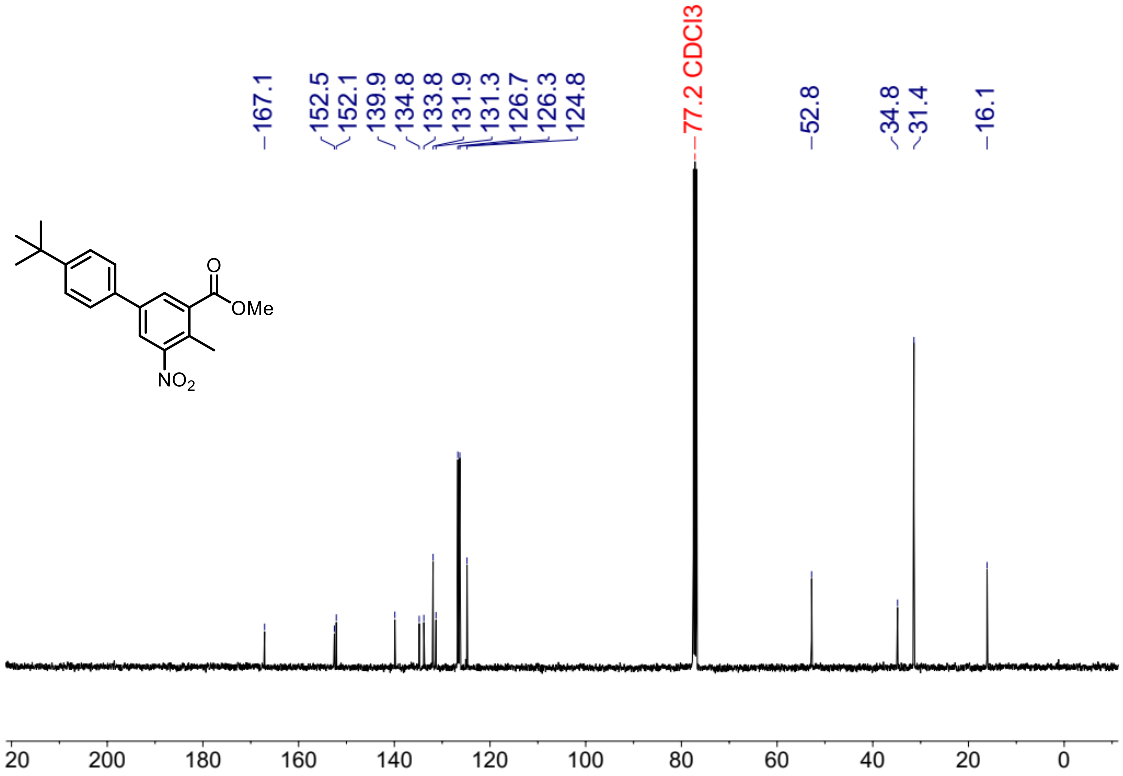

^{1}H NMR (400 MHz, $CDCl_3$) of methyl 2'-fluoro-4-methyl-5-nitro-[1,1'-biphenyl]-3-carboxylate

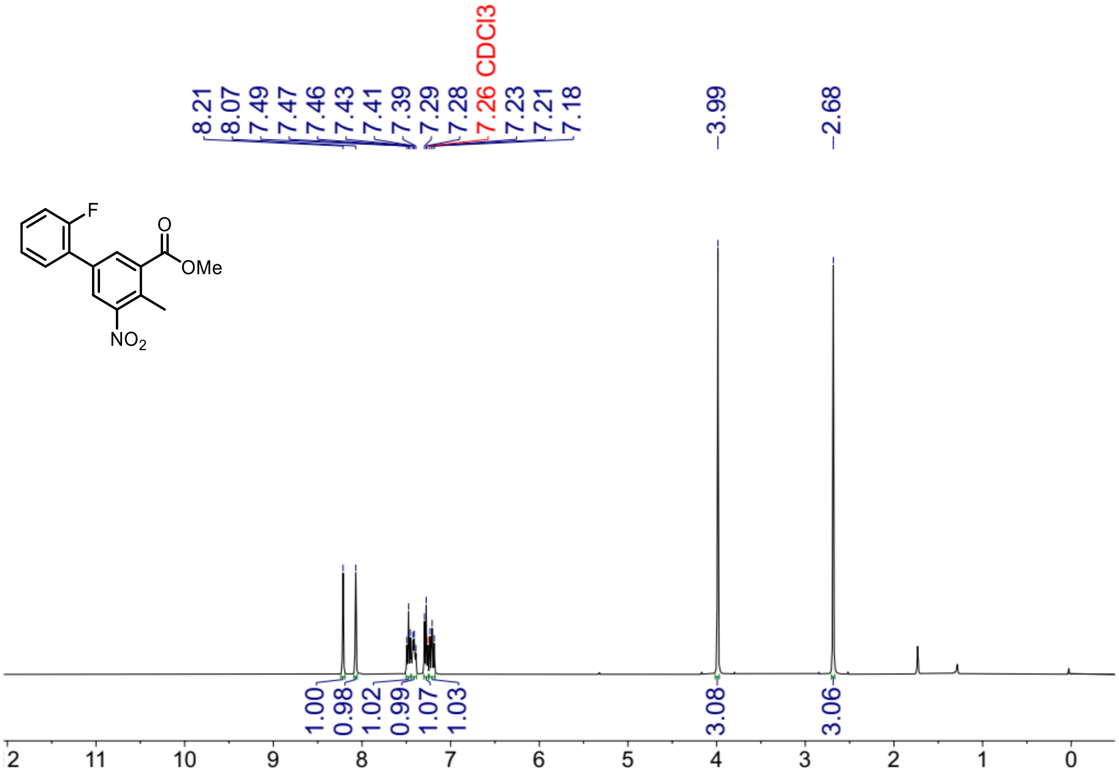

^{13}C NMR (101 MHz, $CDCl_3$) of methyl 2'-fluoro-4-methyl-5-nitro-[1,1'-biphenyl]-3-carboxylate

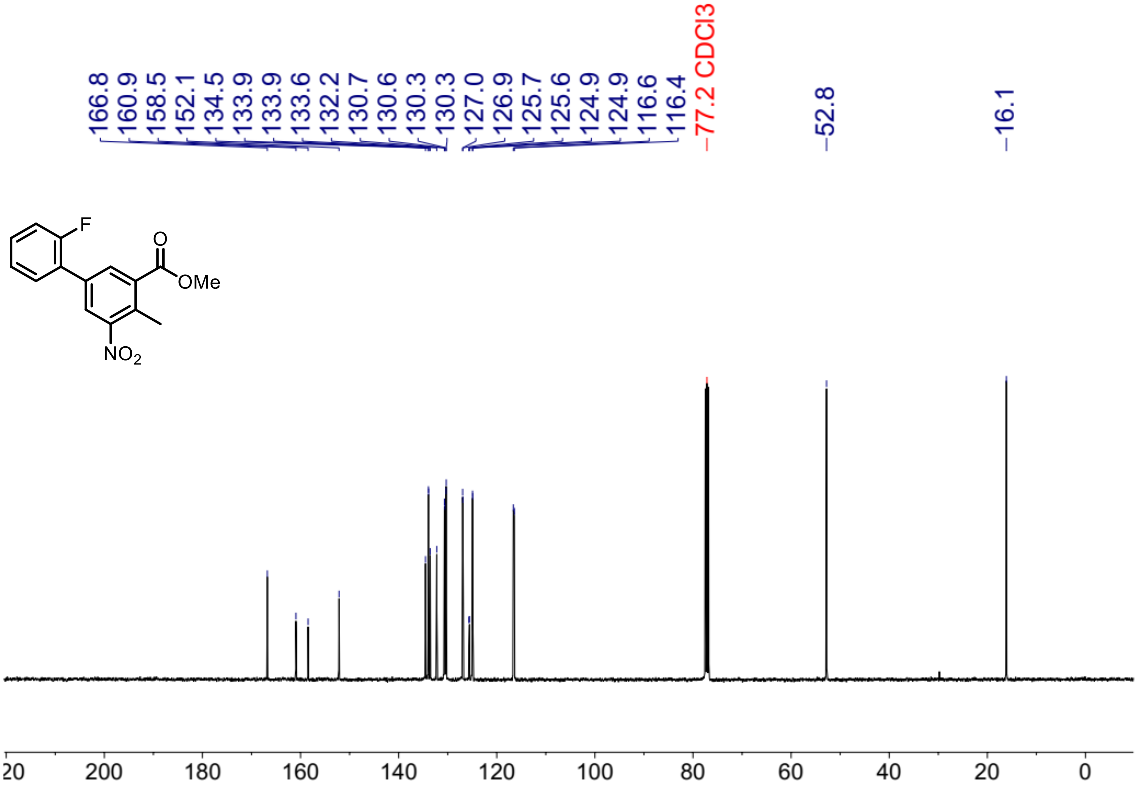

^{19}F NMR (376 MHz, DMSO-d_6) of methyl 2'-fluoro-4-methyl-5-nitro-[1,1'-biphenyl]-3-carboxylate

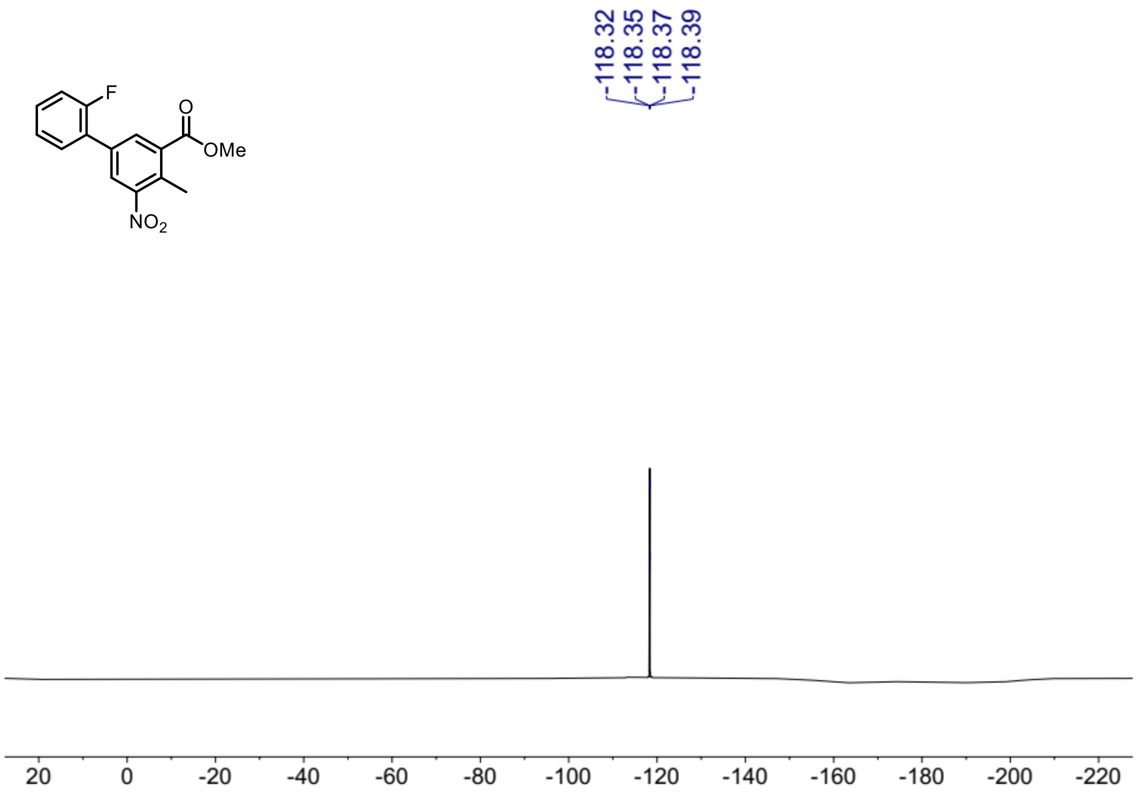

^{1}H NMR (400 MHz, DMSO-d_6) of compound 5b

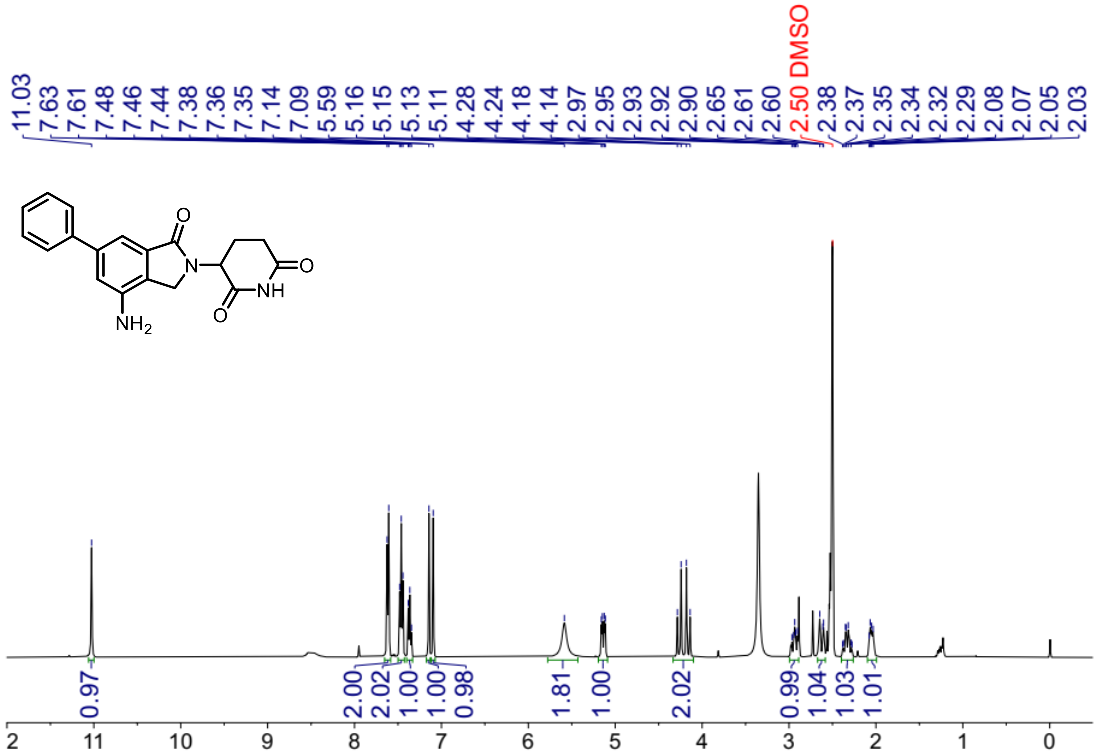

^{13}C NMR (101 MHz, DMSO-d_6) of compound 5b

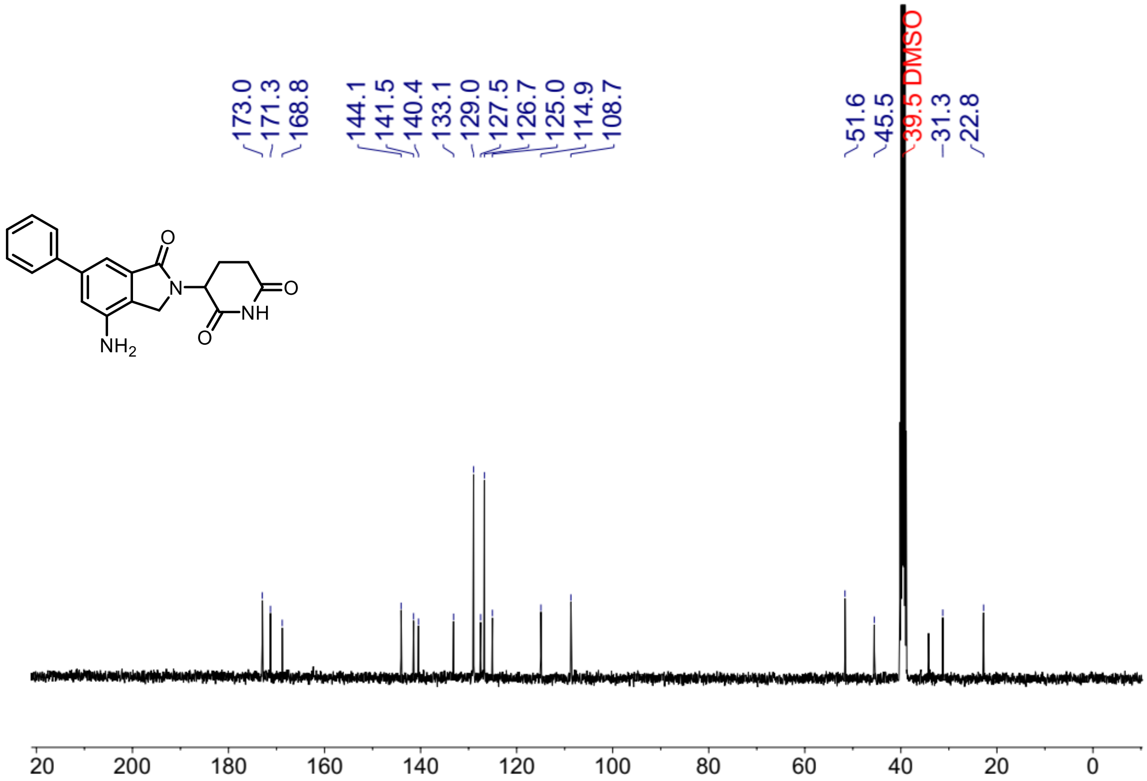

^{1}H NMR (400 MHz, DMSO-d_6) of compound 5c

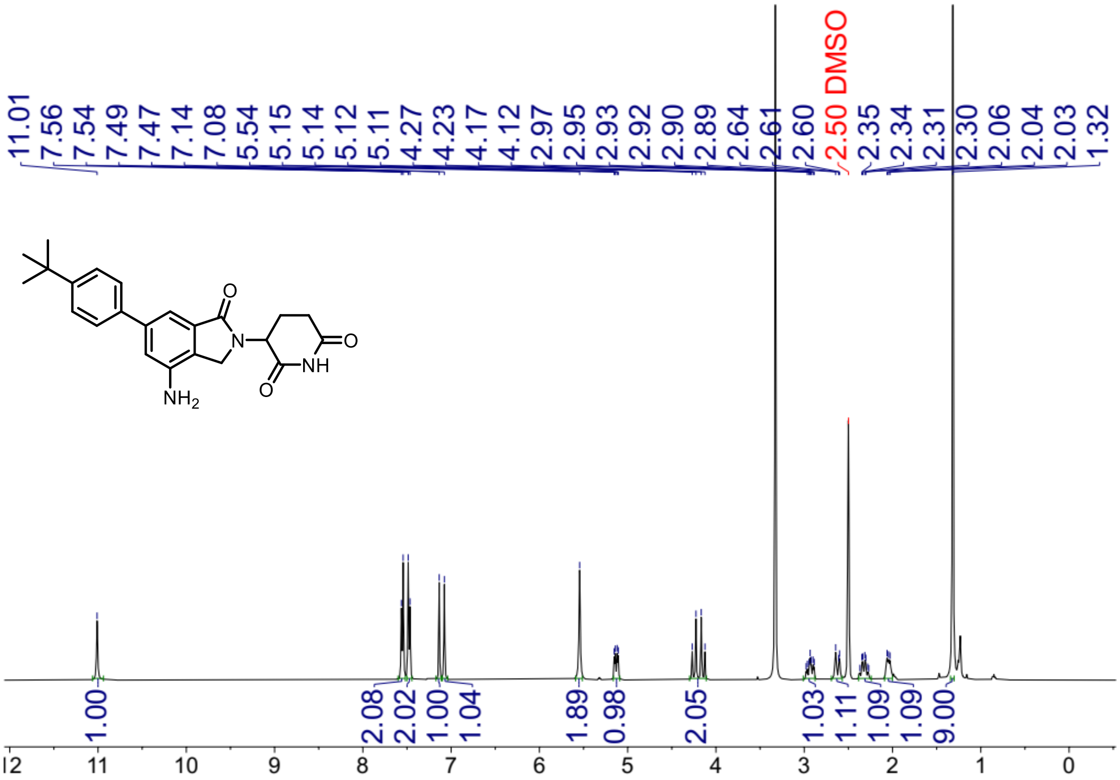

^{13}C NMR (101 MHz, DMSO-d_6) of compound 5c

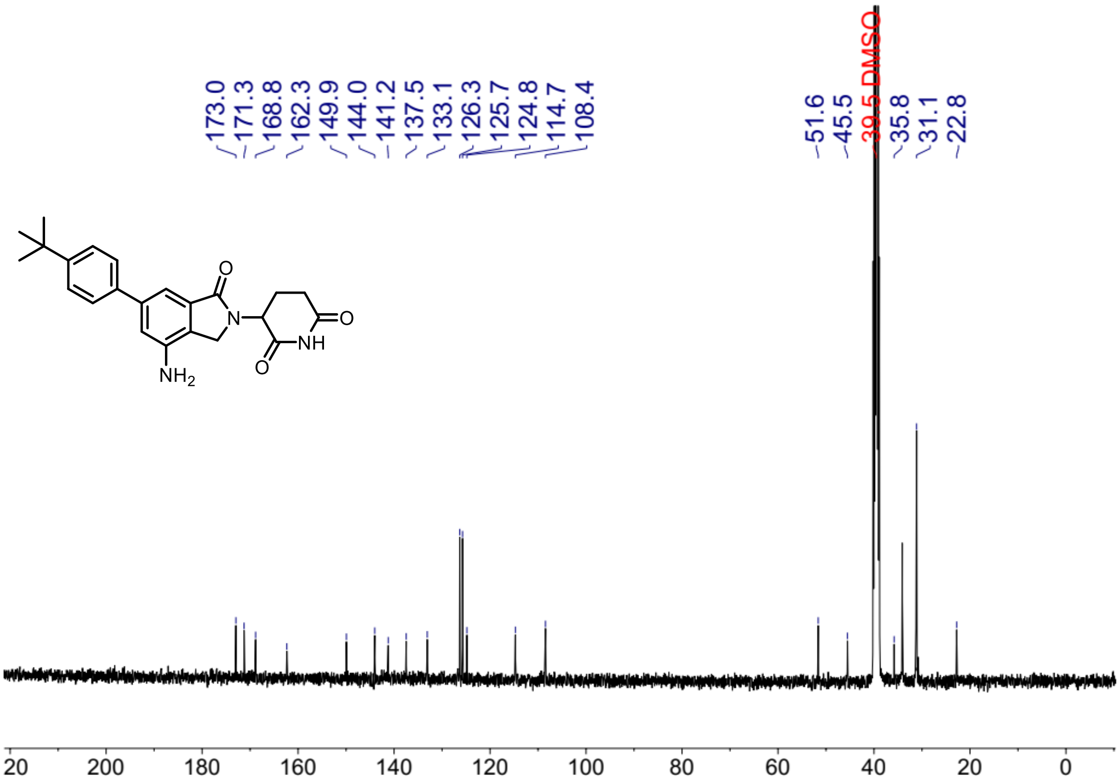

^{1}H NMR (400 MHz, DMSO-d_6) of compound 5d

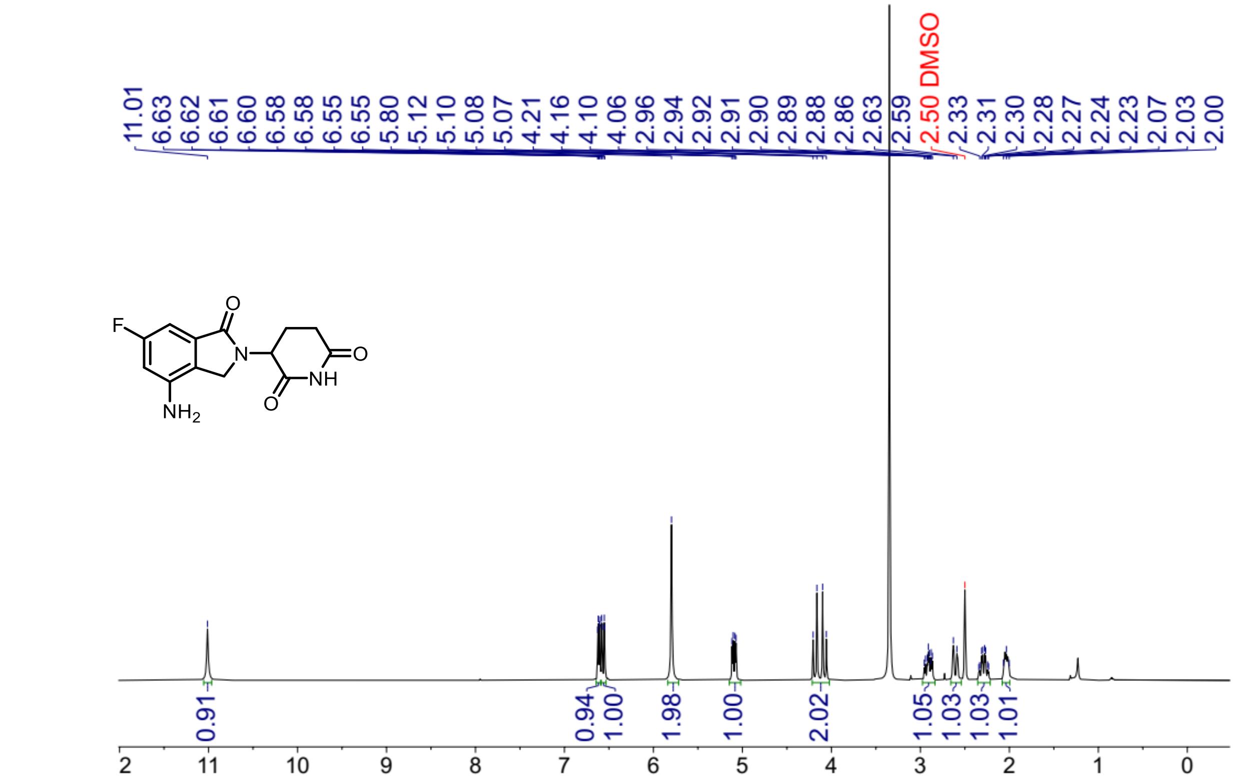

^{13}C NMR (101 MHz, DMSO-d_6) of compound 5d

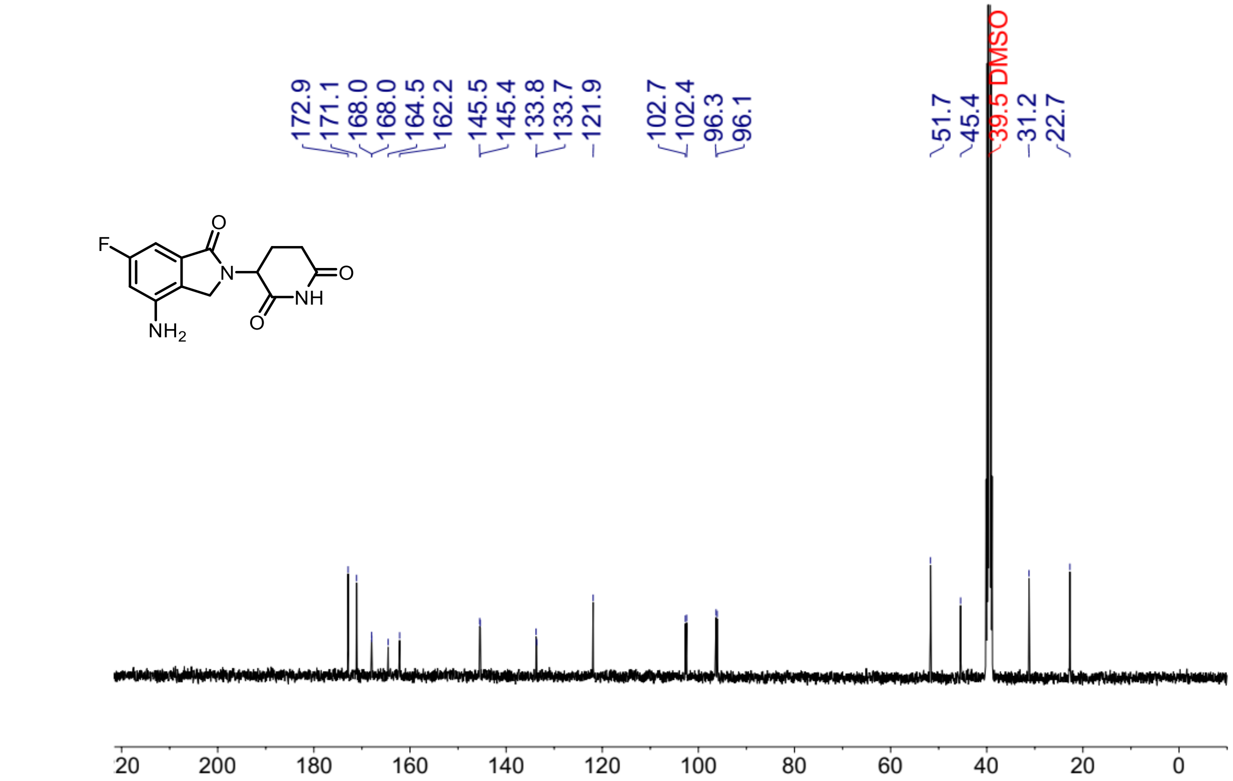

^{19}F NMR (376 MHz, DMSO-d_6) of compound 5d

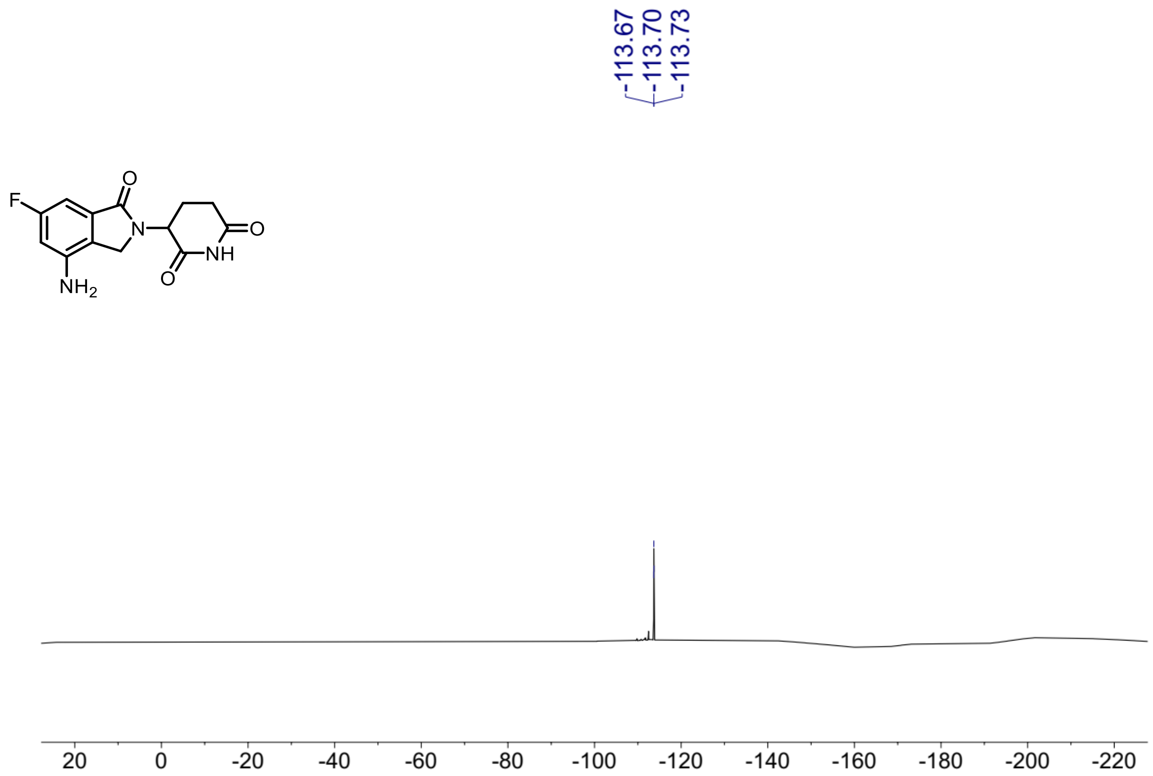

^{1}H NMR (400 MHz, DMSO-d_6) of compound 5e

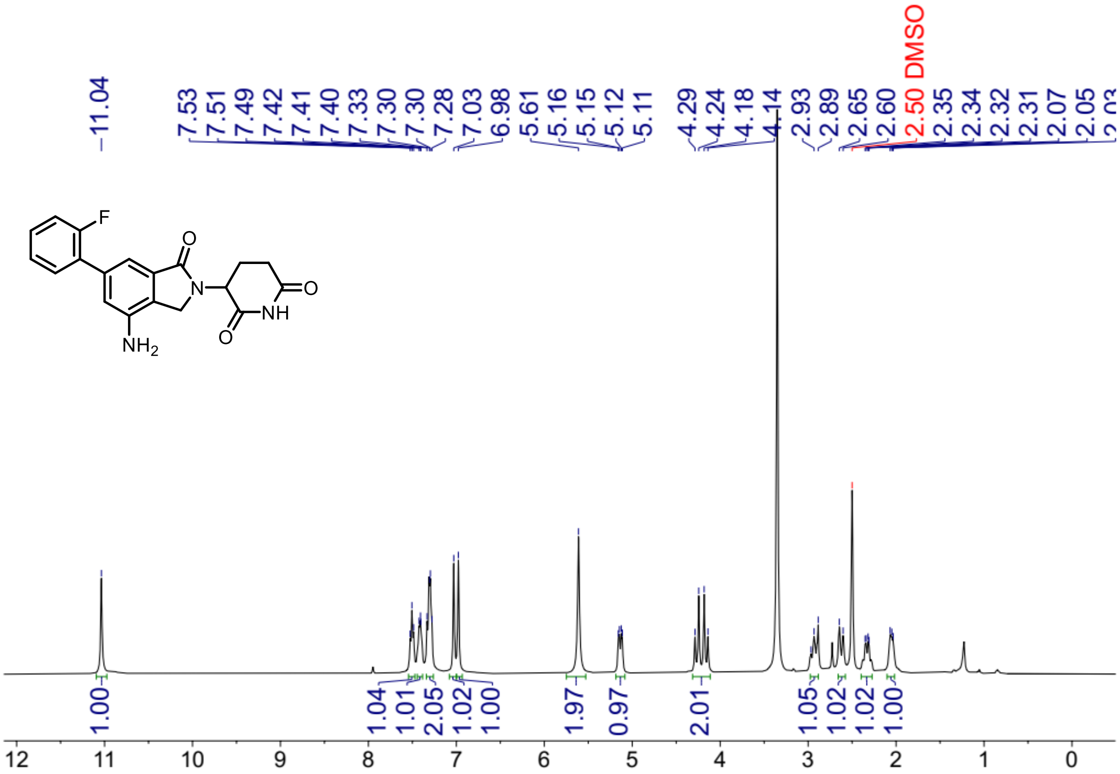

^{13}C NMR (101 MHz, DMSO-d_6) of compound 5e

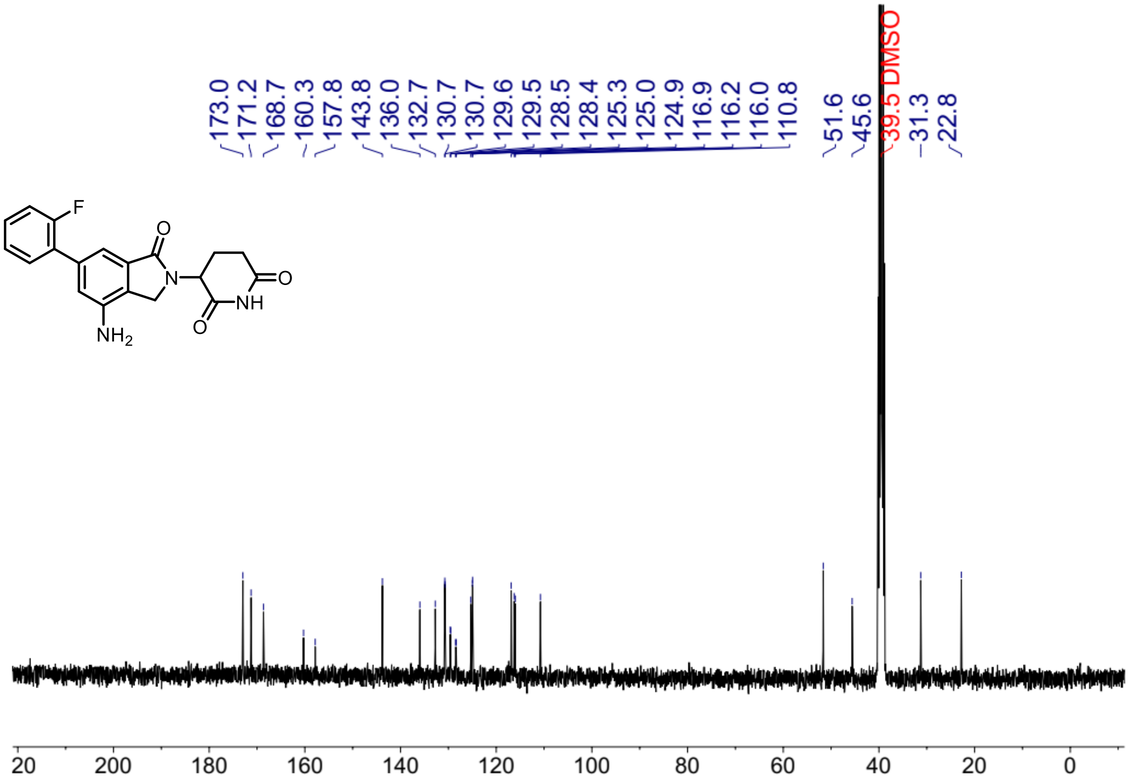

^{19}F NMR (376 MHz, DMSO-d_6) of compound 5e

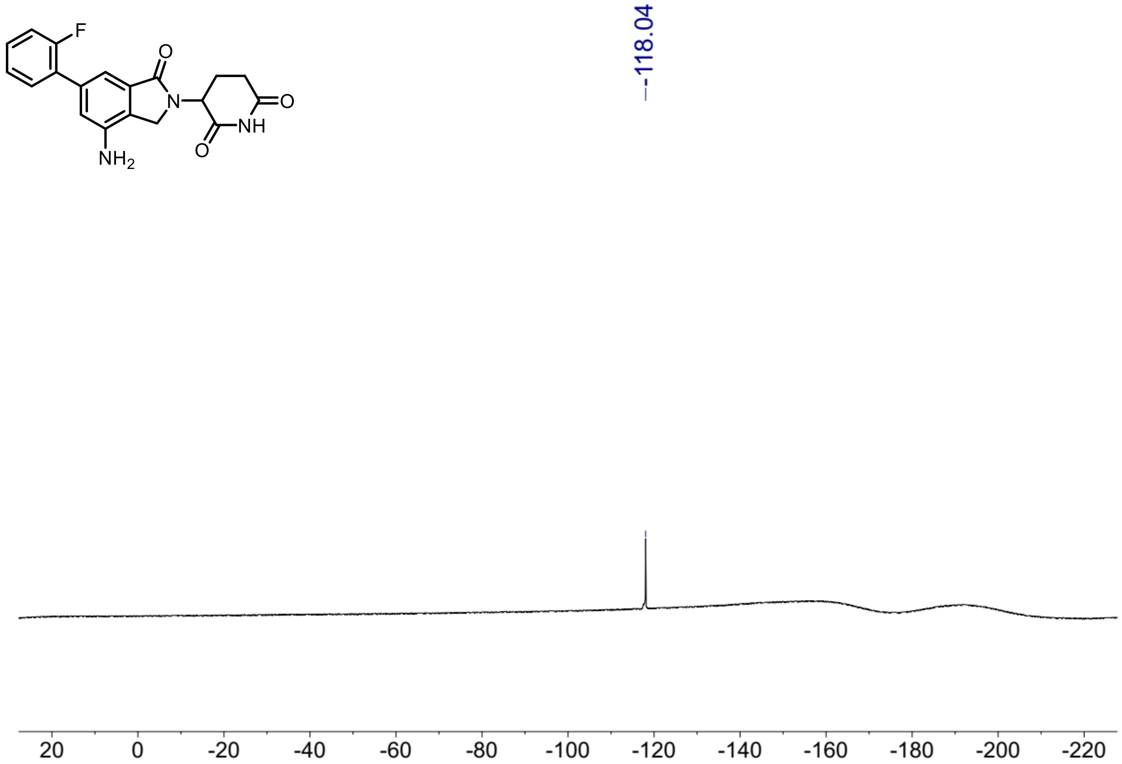

^{1}H NMR (400 MHz, DMSO-*d*$_6$) of compound 6

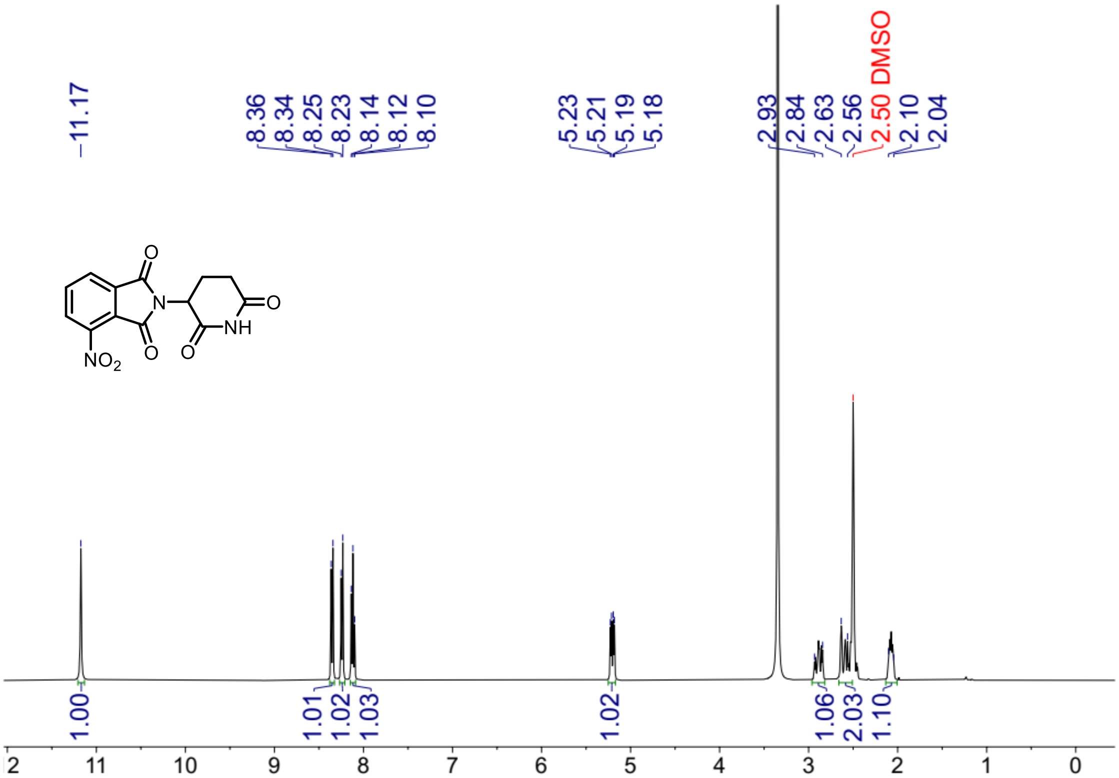

^{13}C NMR (101 MHz, DMSO-*d*$_6$) of compound 6

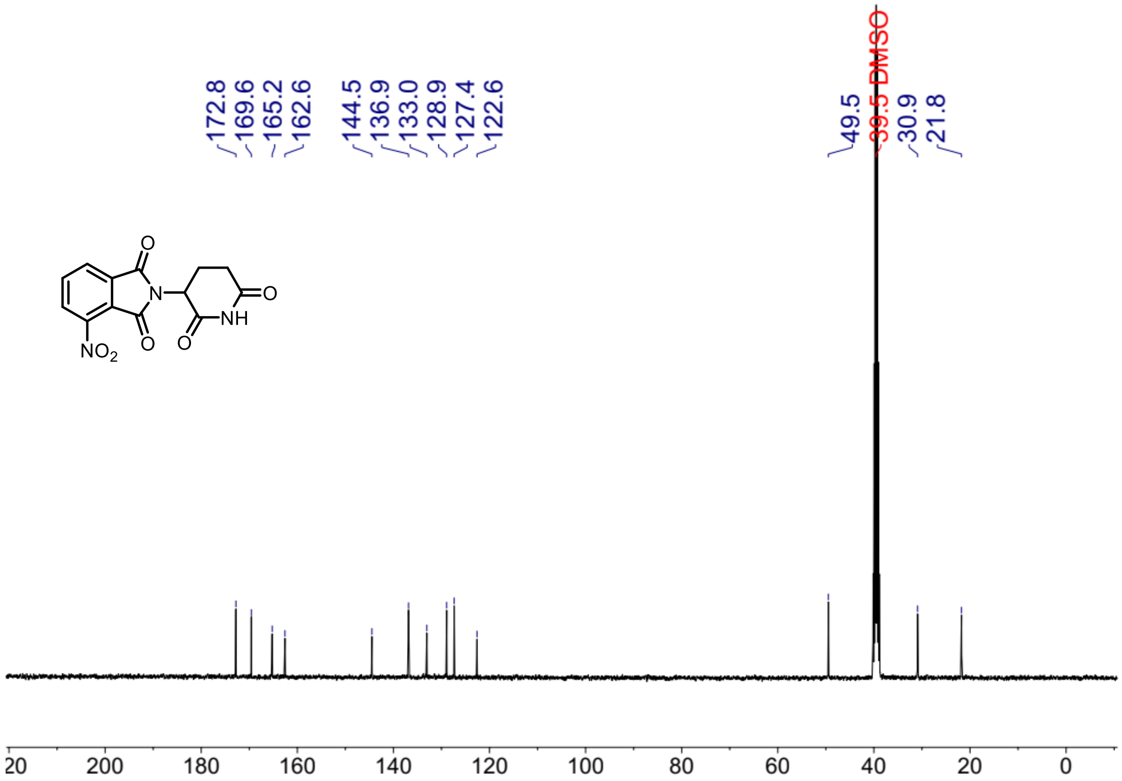

^{1}H NMR (400 MHz, DMSO-d_6) of compound 7

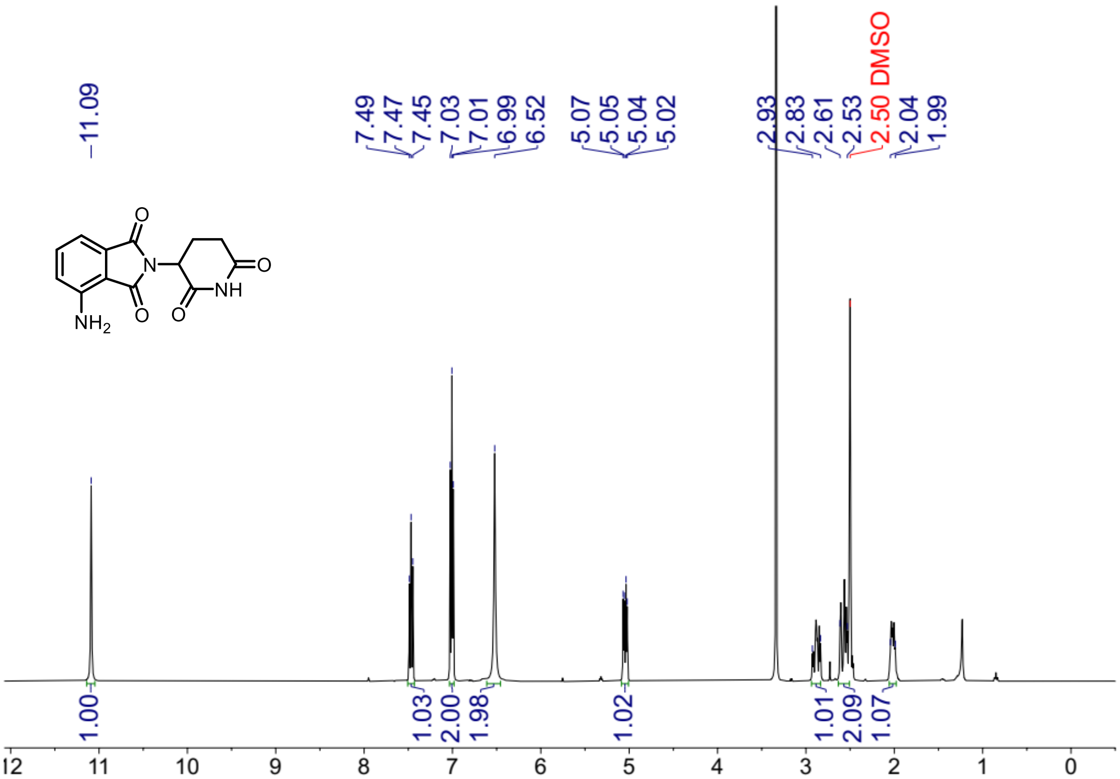

^{13}C NMR (101 MHz, DMSO-d_6) of compound 7

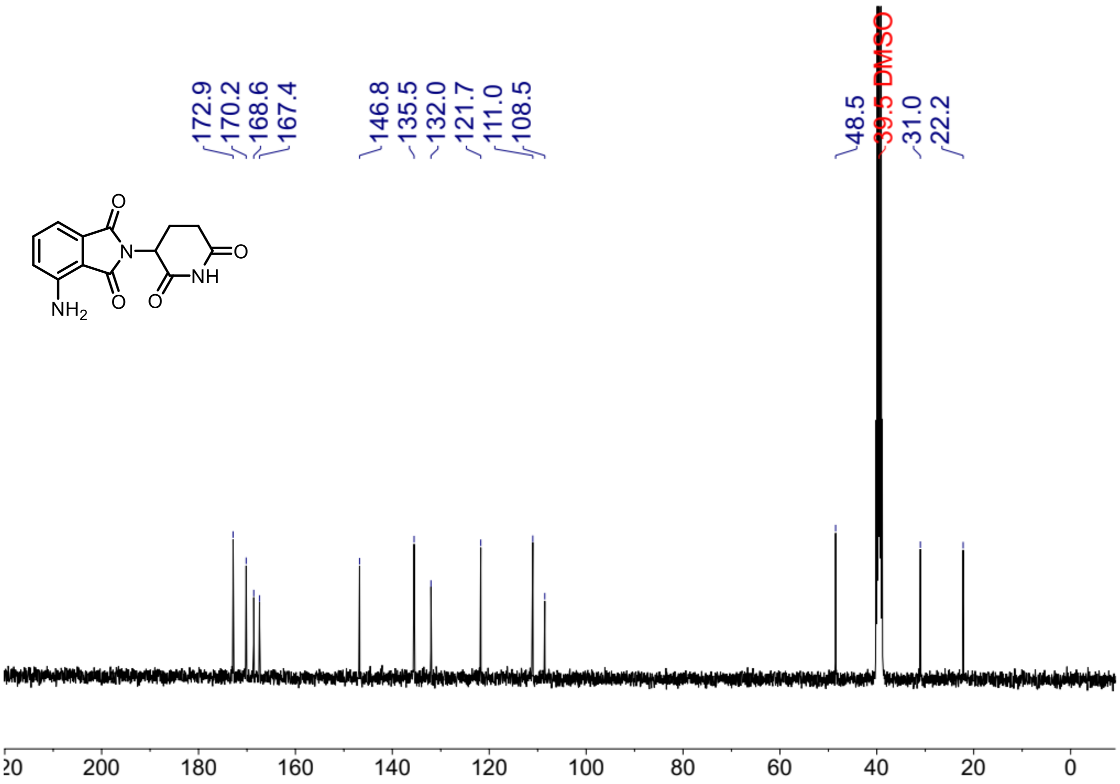

^{1}H NMR (400 MHz, DMSO-d_6) of compound 8

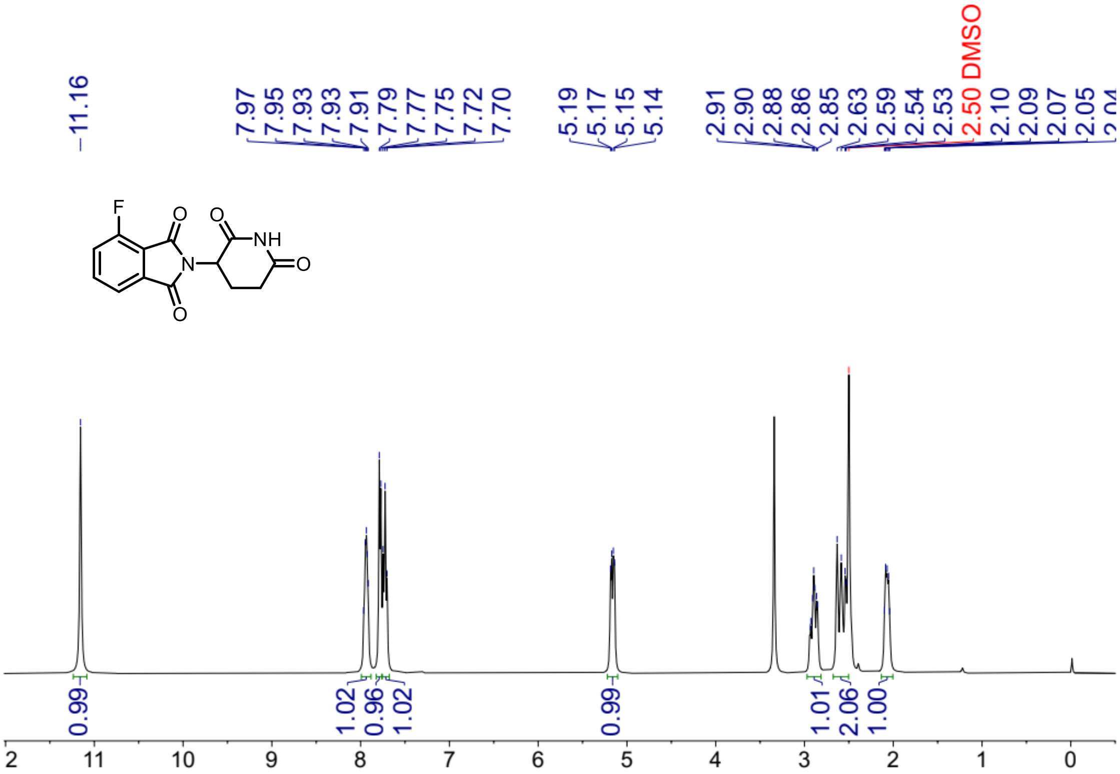

^{13}C NMR (101 MHz, DMSO-d_6) of compound 8

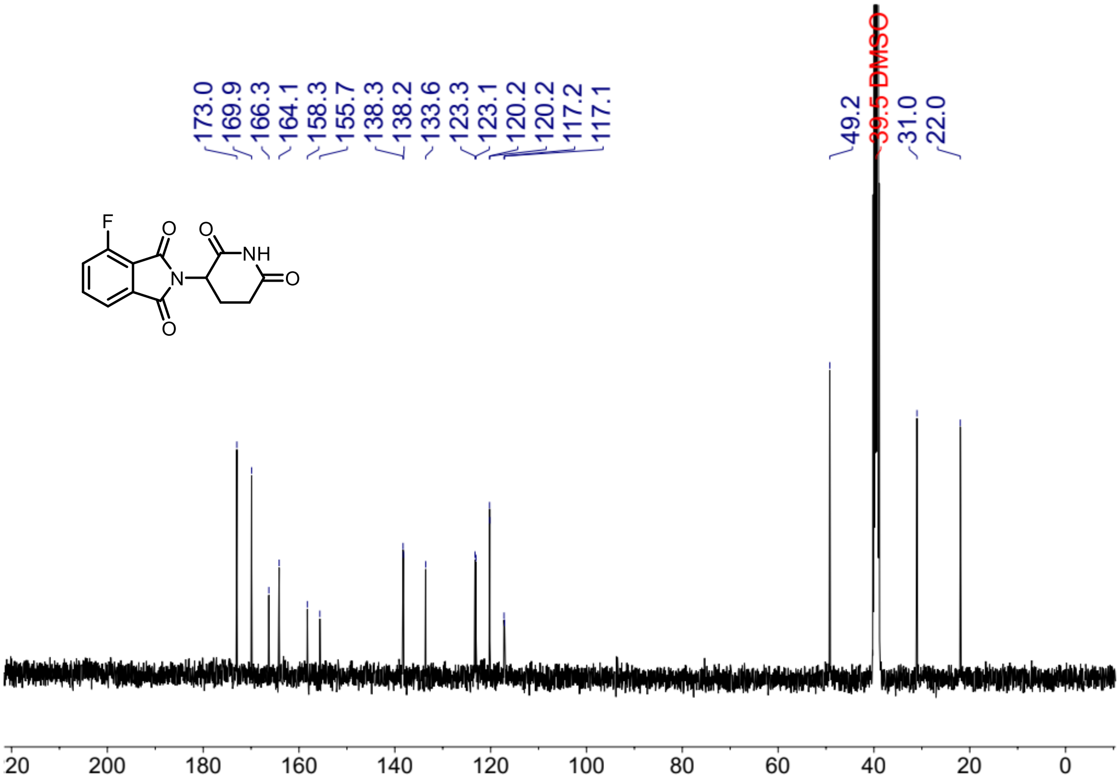

^{19}F NMR (376 MHz, DMSO-d_6) of compound 8

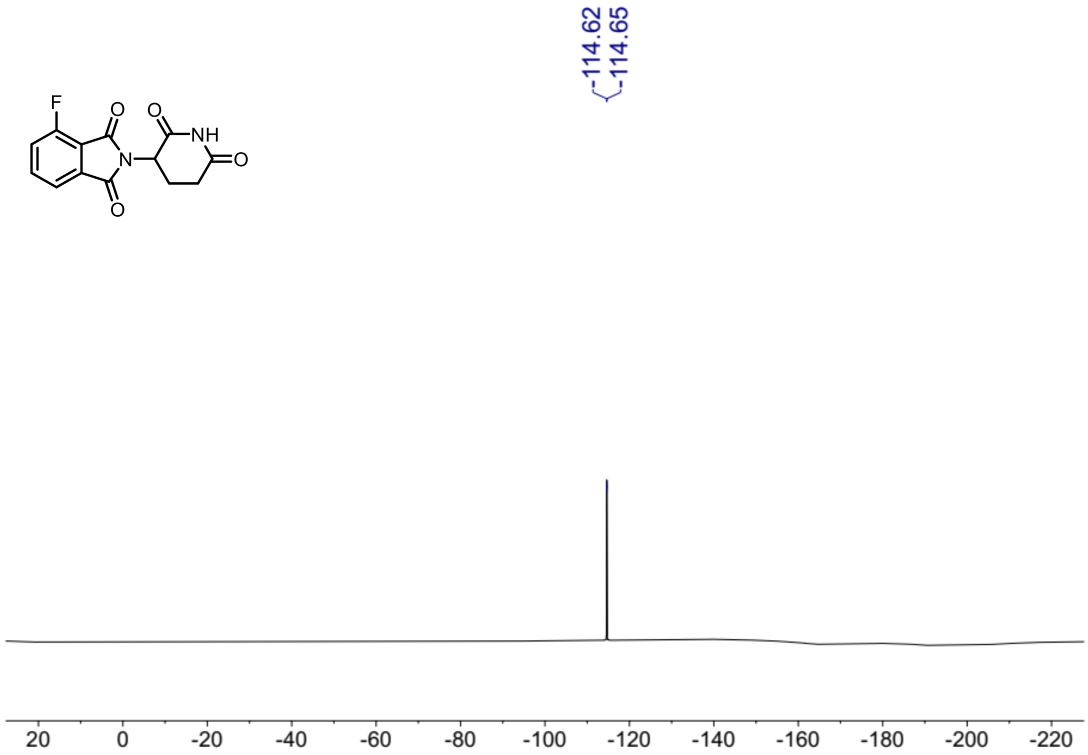

^{1}H NMR (400 MHz, DMSO-d_6) of compound 9a

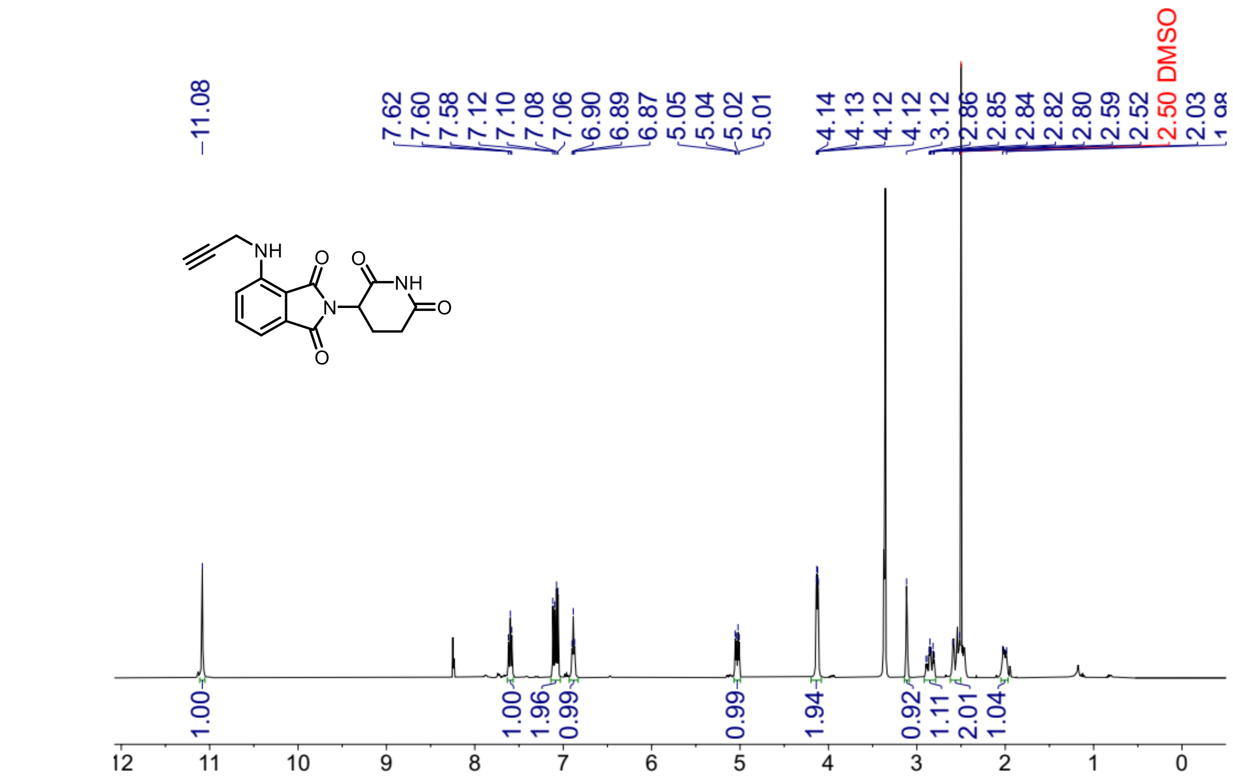

^{13}C NMR (101 MHz, DMSO-d_6) of compound 9a

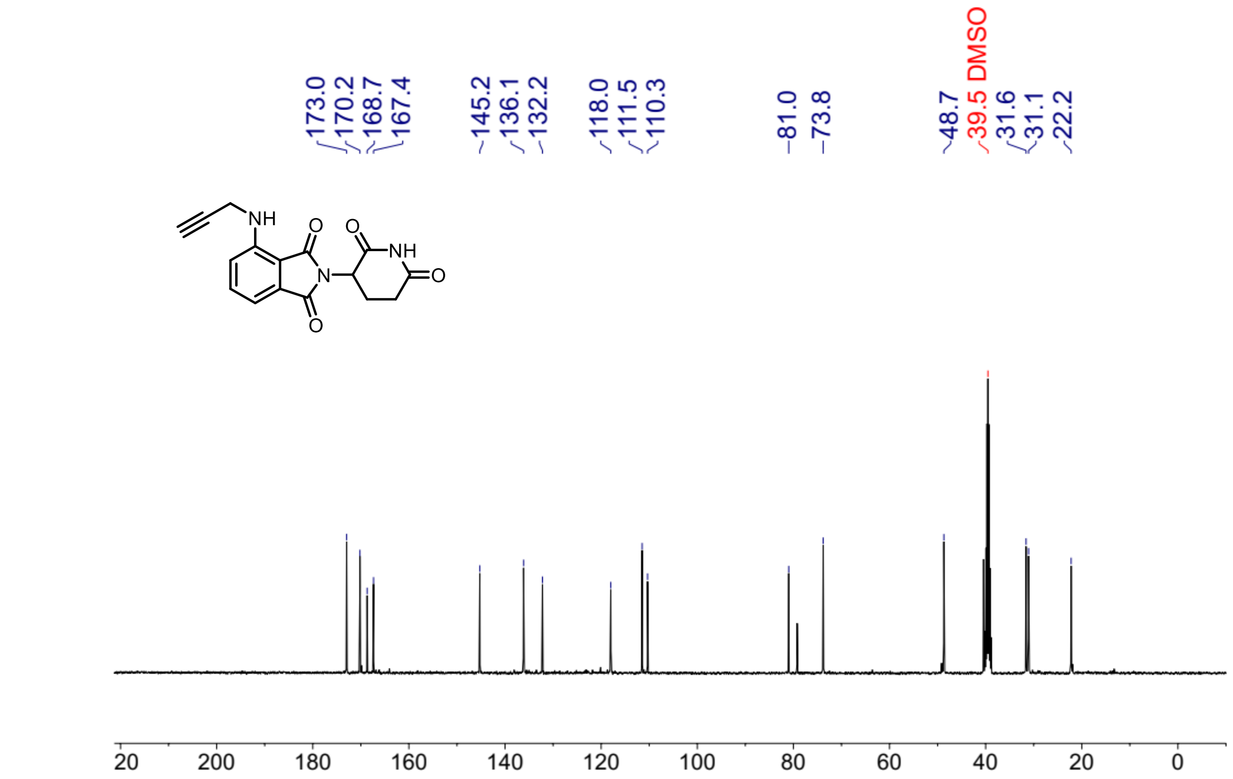

^{1}H NMR (400 MHz, DMSO-d_6) of compound 9b

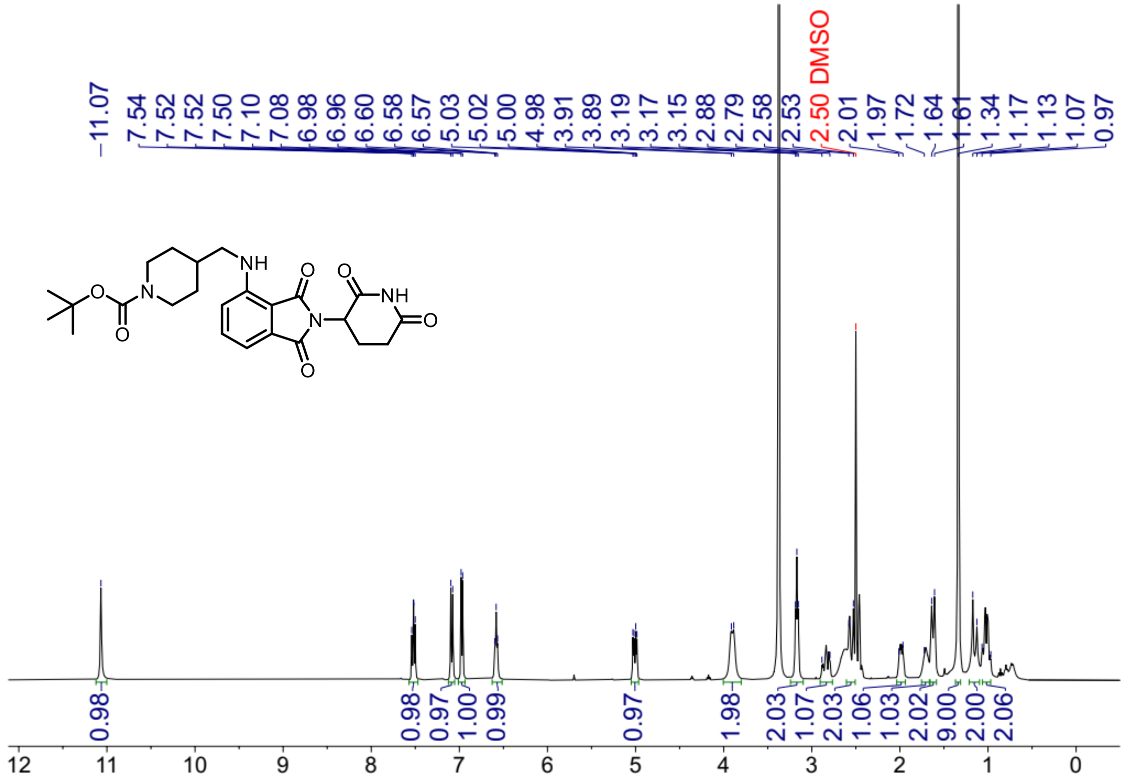

^{13}C NMR (101 MHz, DMSO-d_6) of compound 9b

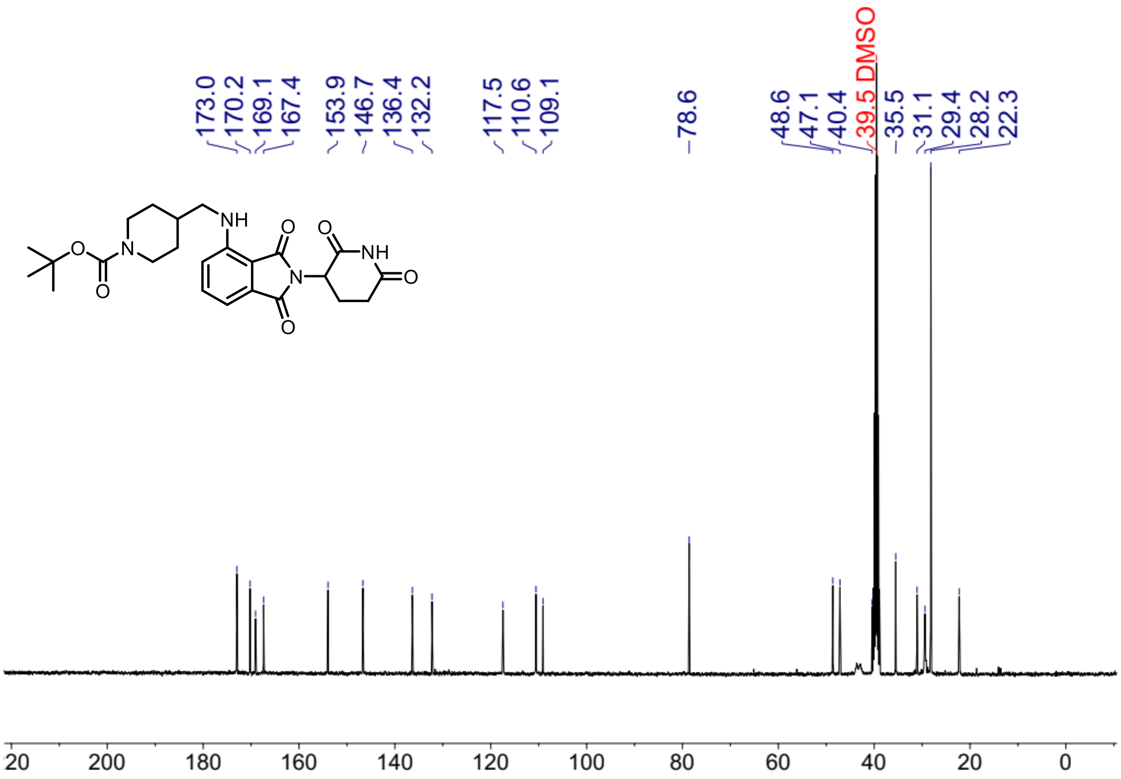

^{1}H NMR (400 MHz, DMSO-d_6) of compound 9c

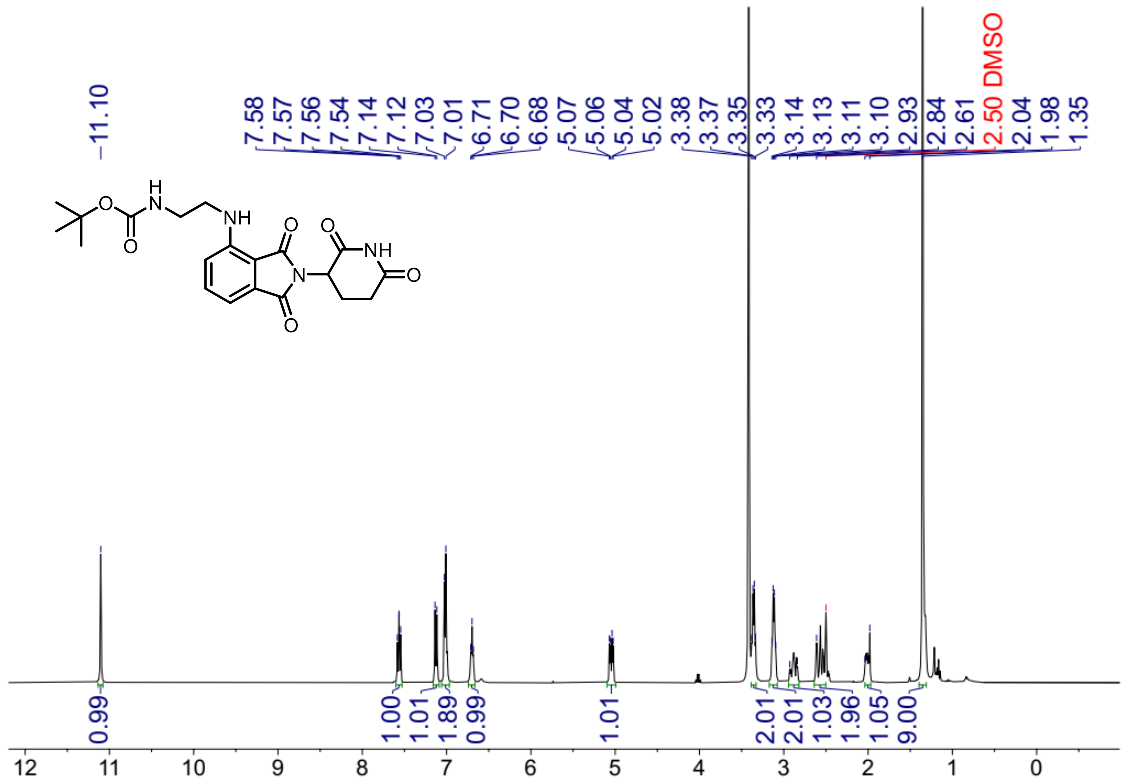

^{13}C NMR (101 MHz, DMSO-d_6) of compound 9c

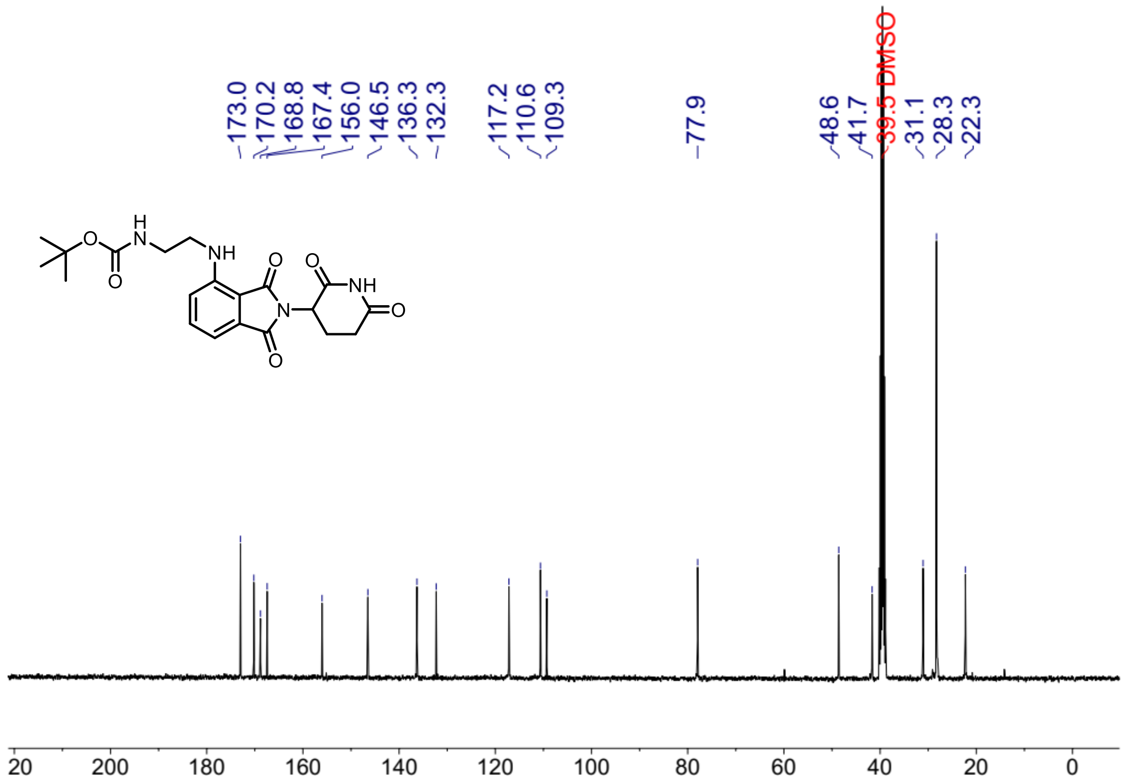

^{1}H NMR (400 MHz, DMSO-d_6) of compound 9d

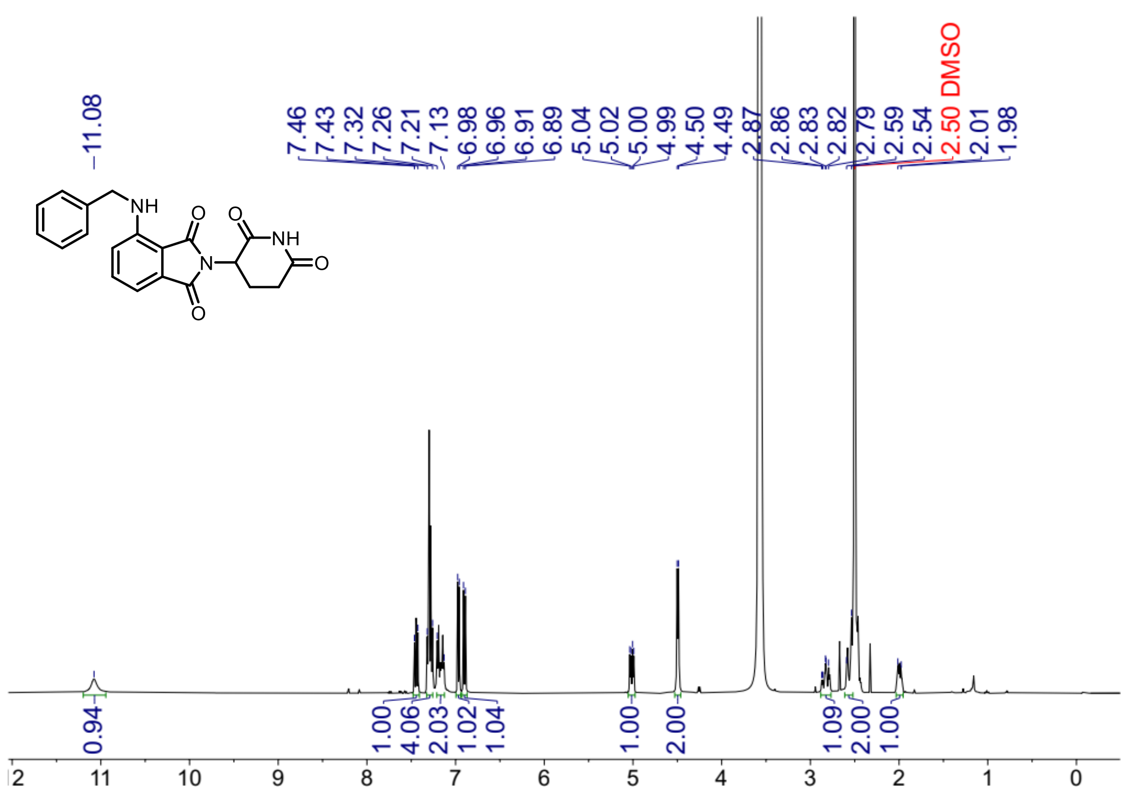

^{13}C NMR (101 MHz, DMSO-d_6) of compound 9d

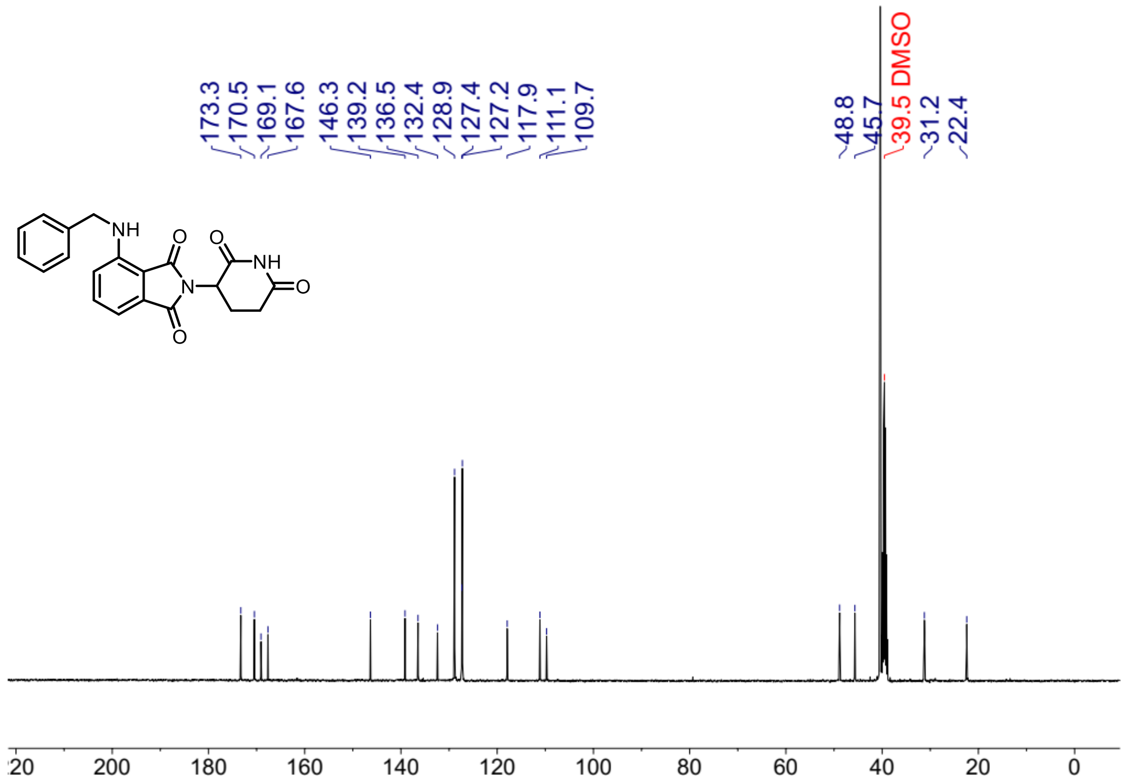

^{1}H NMR (400 MHz, DMSO-d_6) of compound 9e

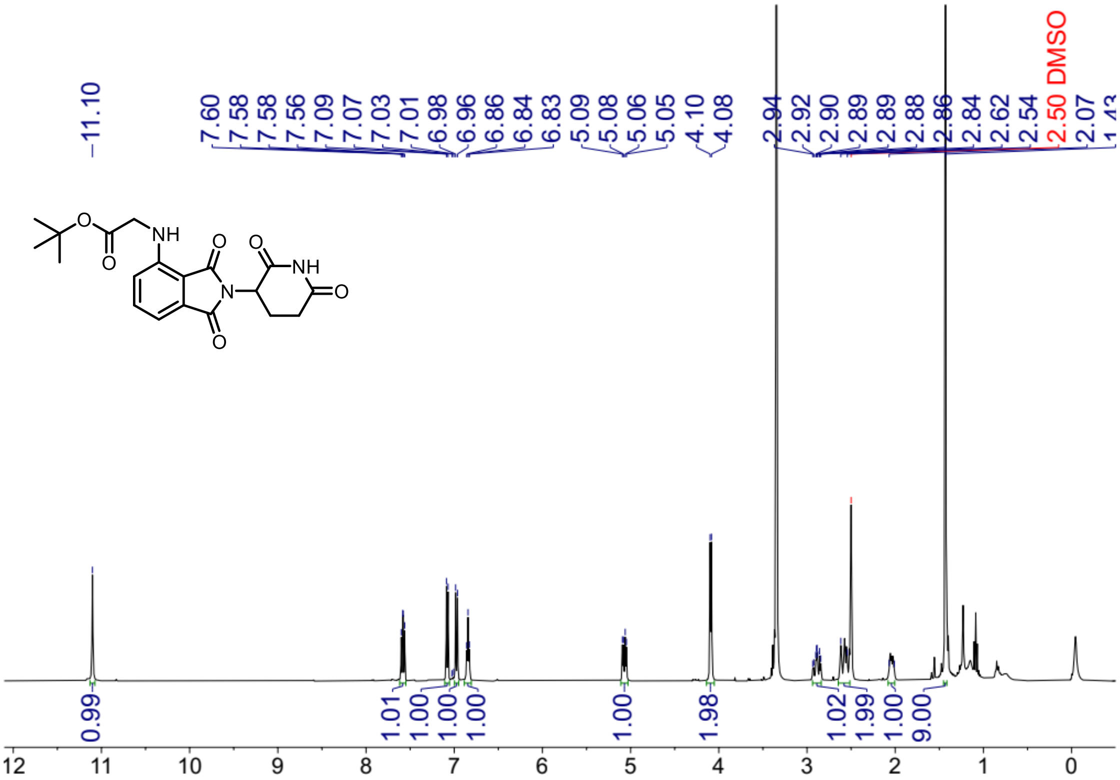

^{13}C NMR (101 MHz, DMSO-d_6) of compound 9e

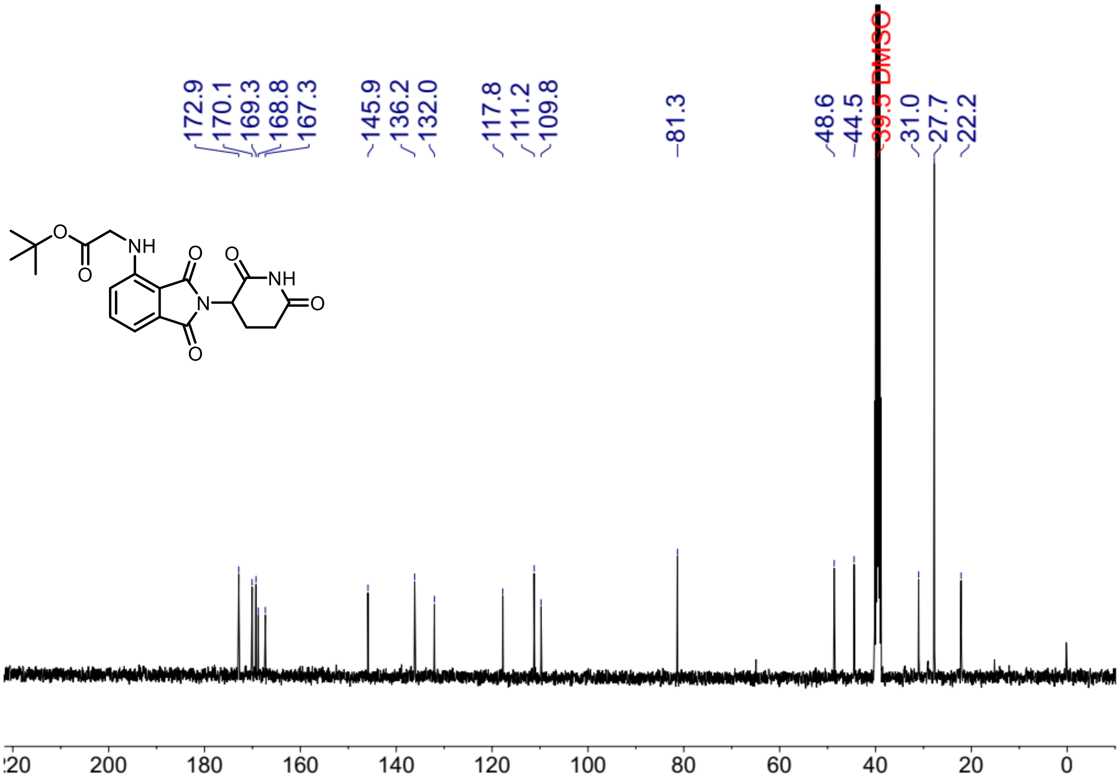

^{1}H NMR (400 MHz, DMSO-d_6) of compound 9f

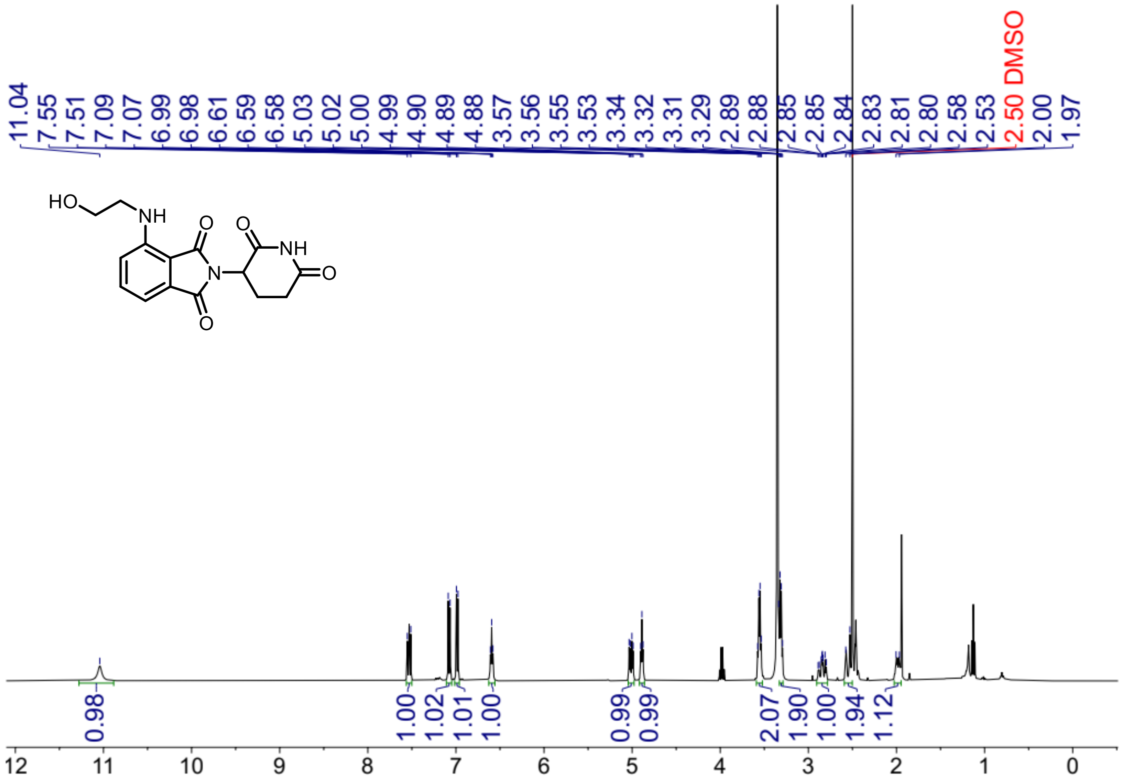

^{13}C NMR (101 MHz, DMSO-d_6) of compound 9f

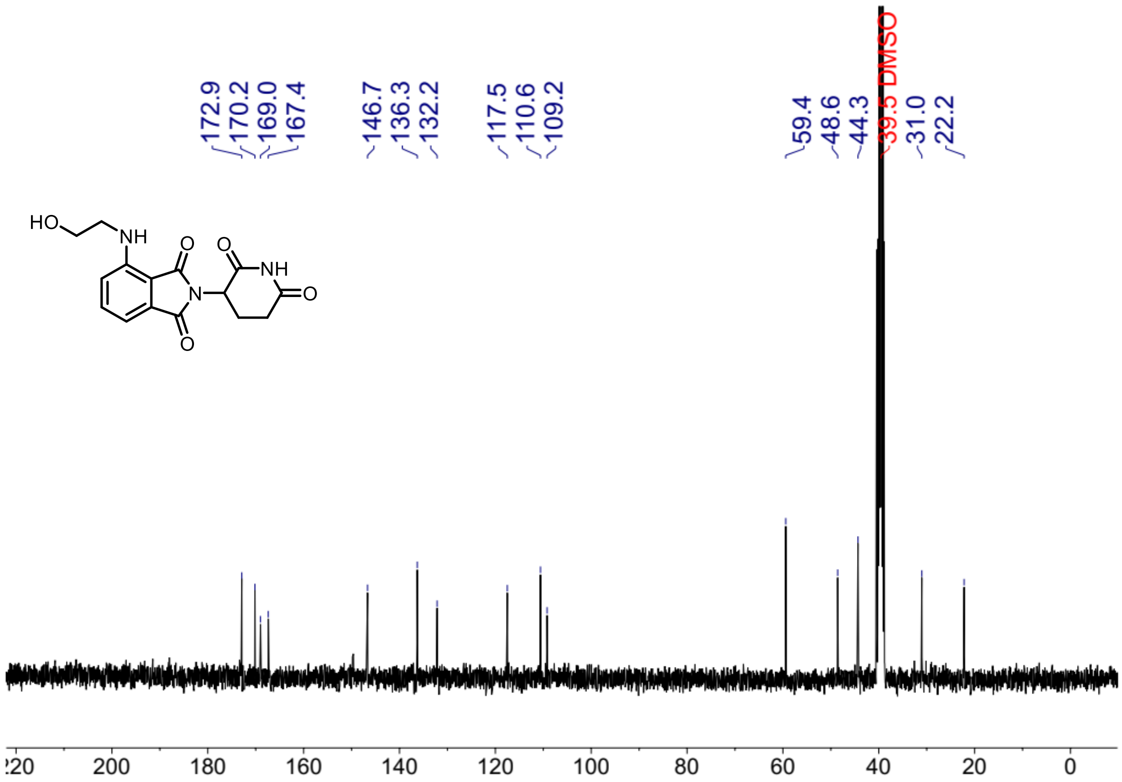

^{1}H NMR (400 MHz, DMSO-d_6) of compound 9g

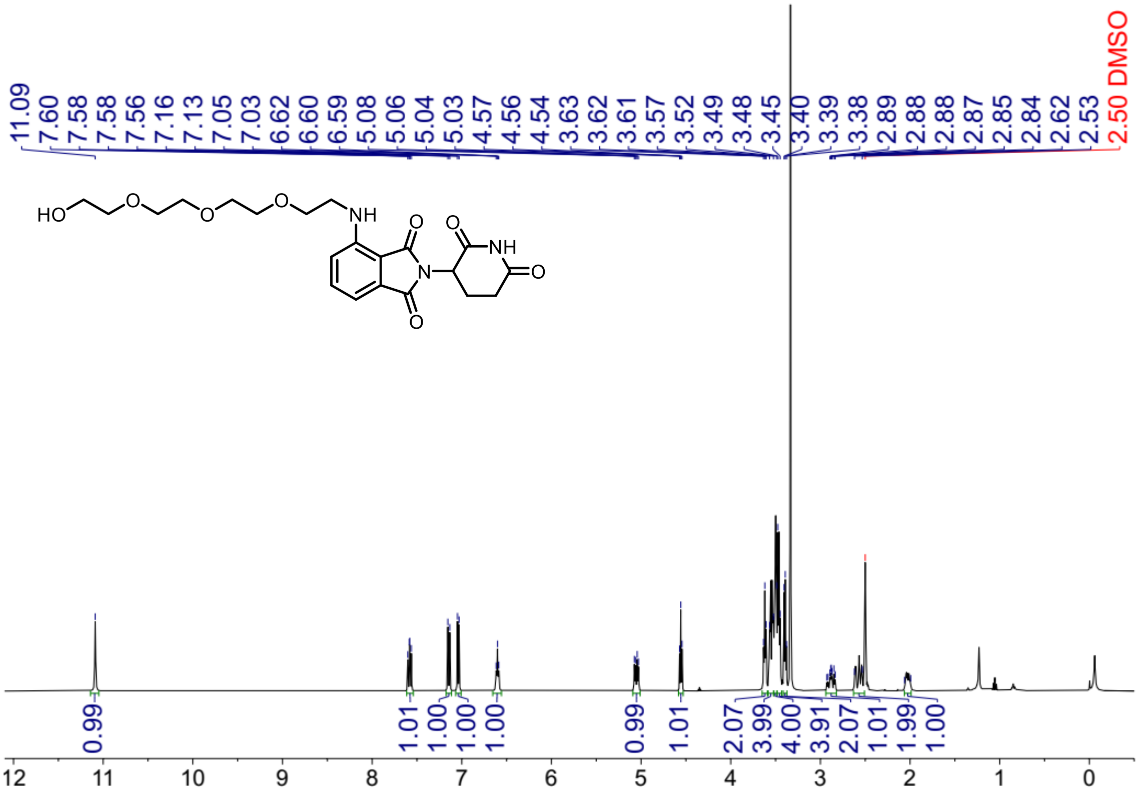

^{13}C NMR (101 MHz, DMSO-d_6) of compound 9g

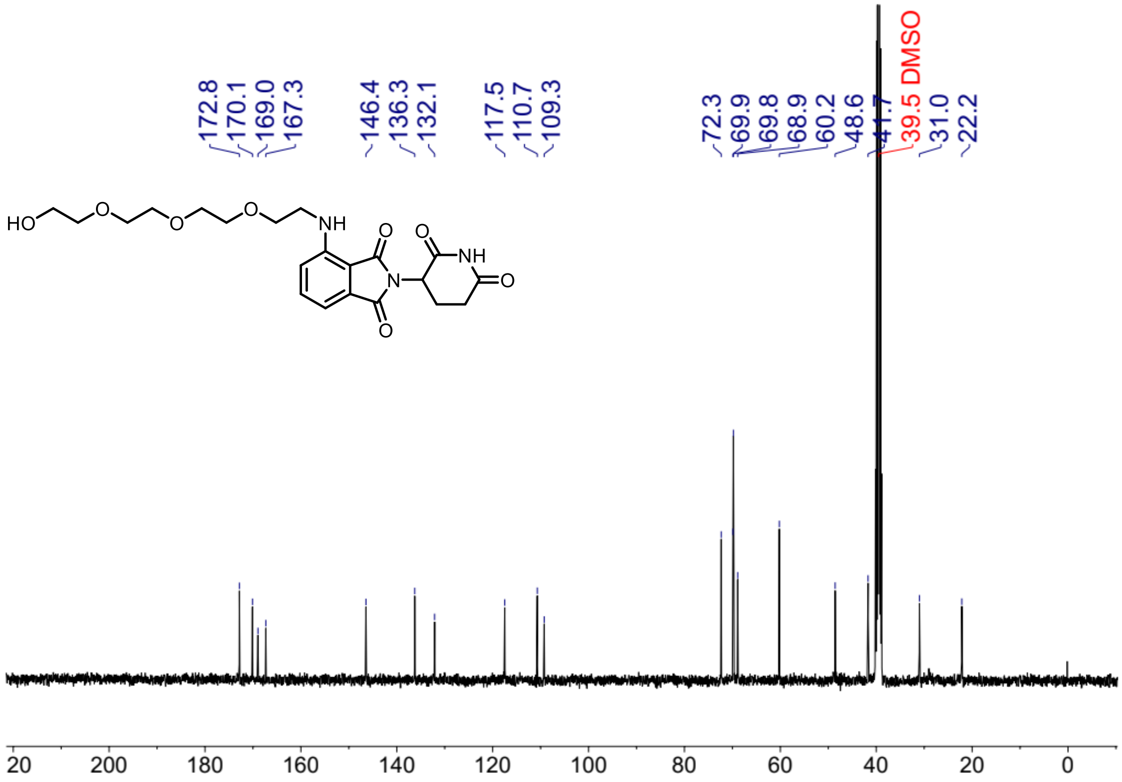

^{1}H NMR (400 MHz, DMSO-d_6) of compound 9h

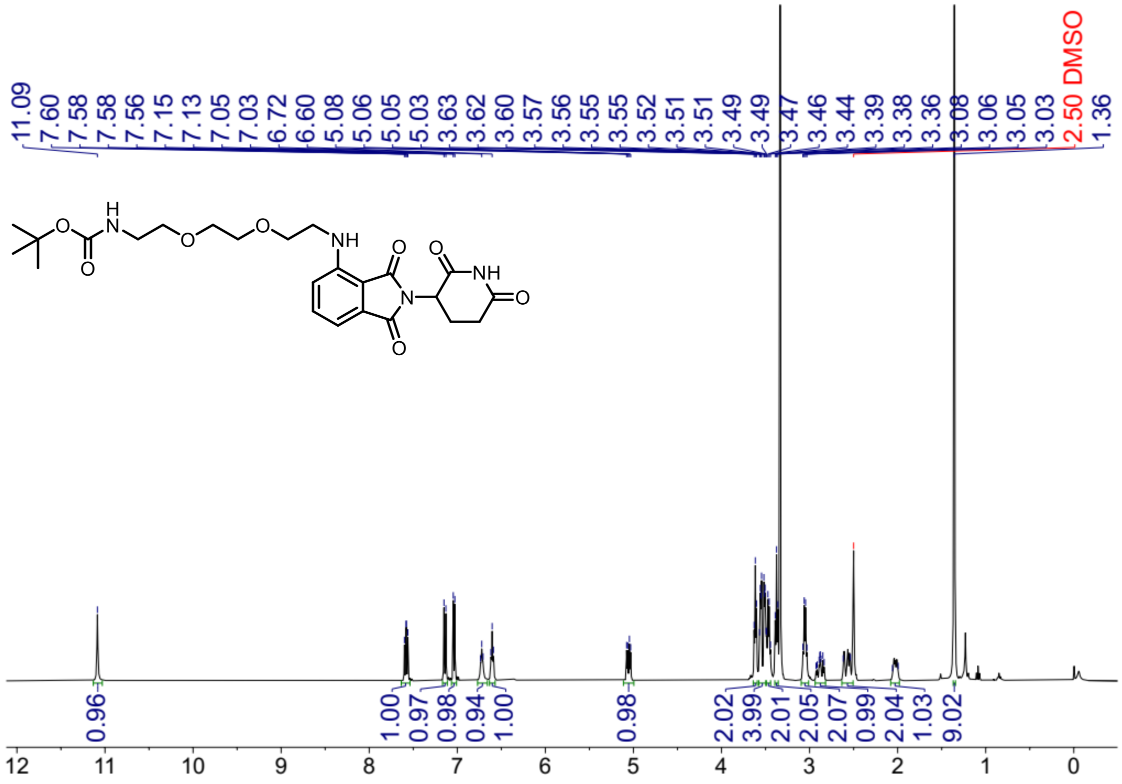

^{13}C NMR (101 MHz, DMSO-d_6) of compound 9h

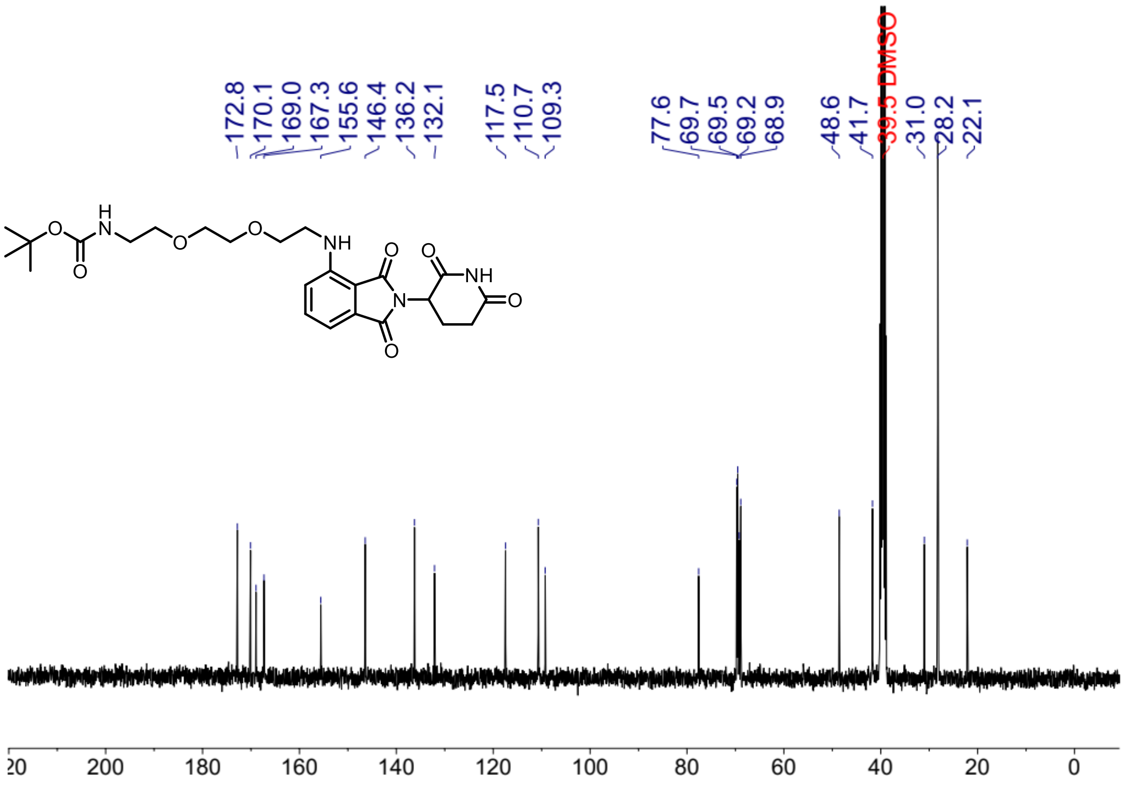

^{1}H NMR (400 MHz, DMSO-d_6) of compound 9i

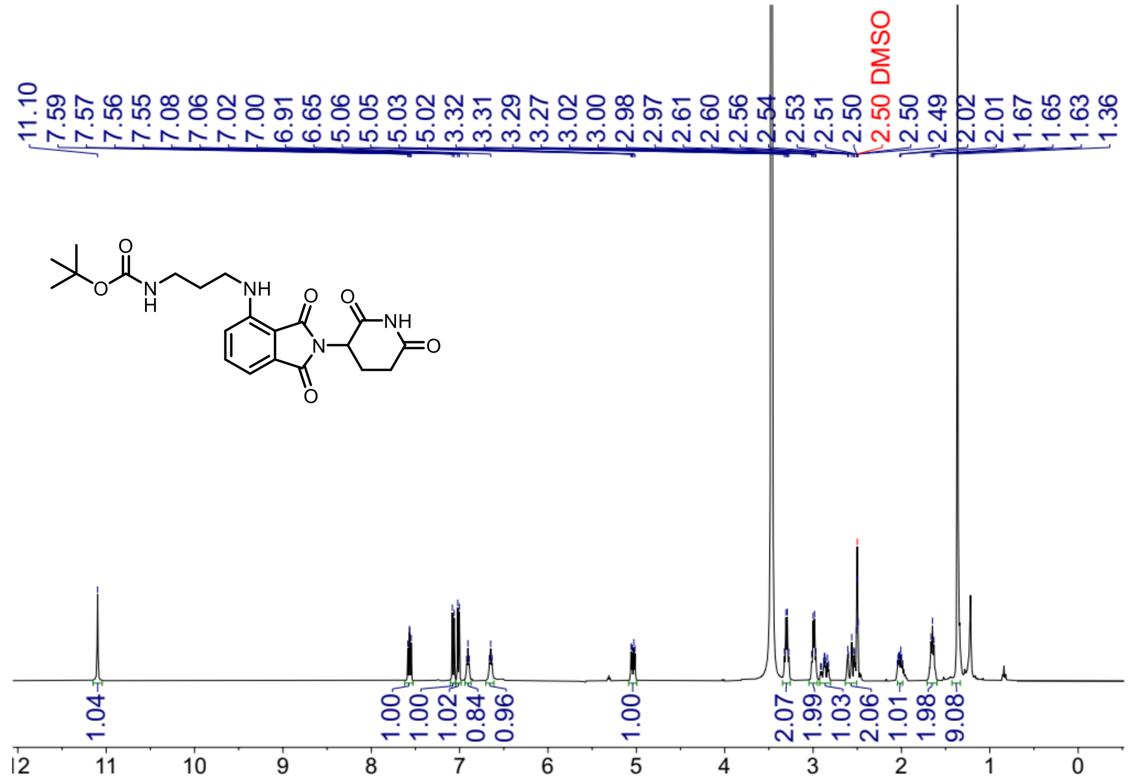

^{13}C NMR (101 MHz, DMSO-d_6) of compound 9i

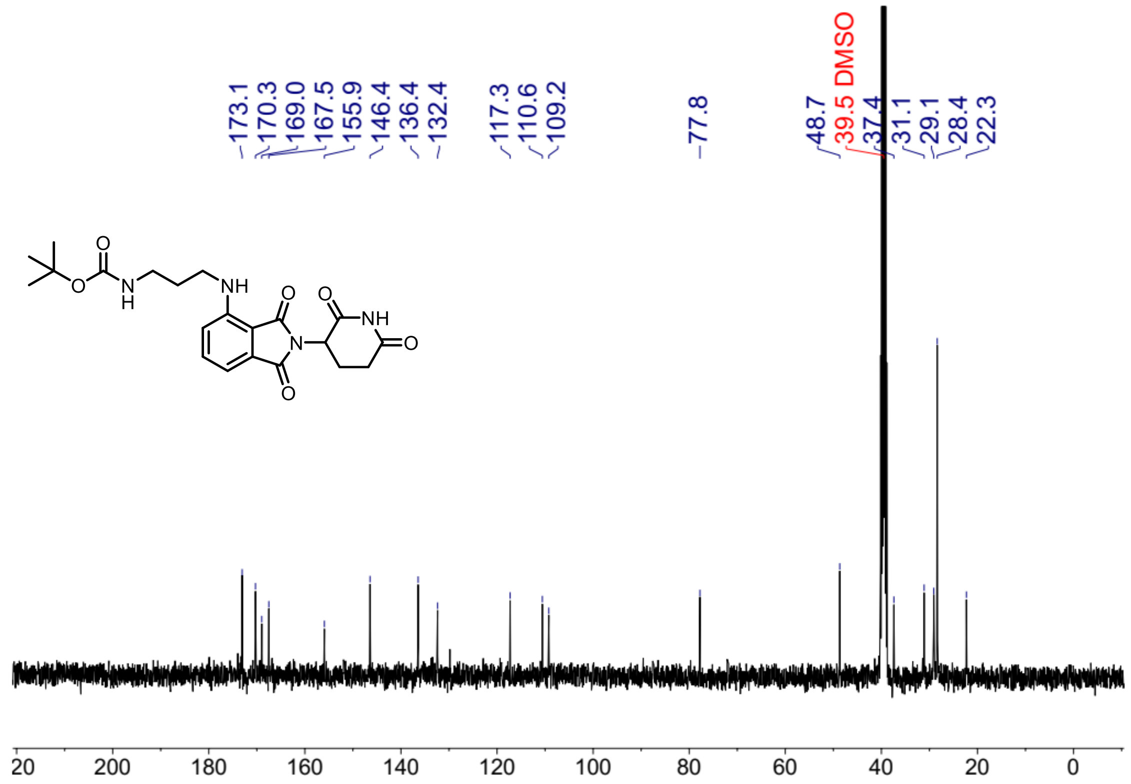

^{1}H NMR (400 MHz, DMSO-d_6) of compound 9j

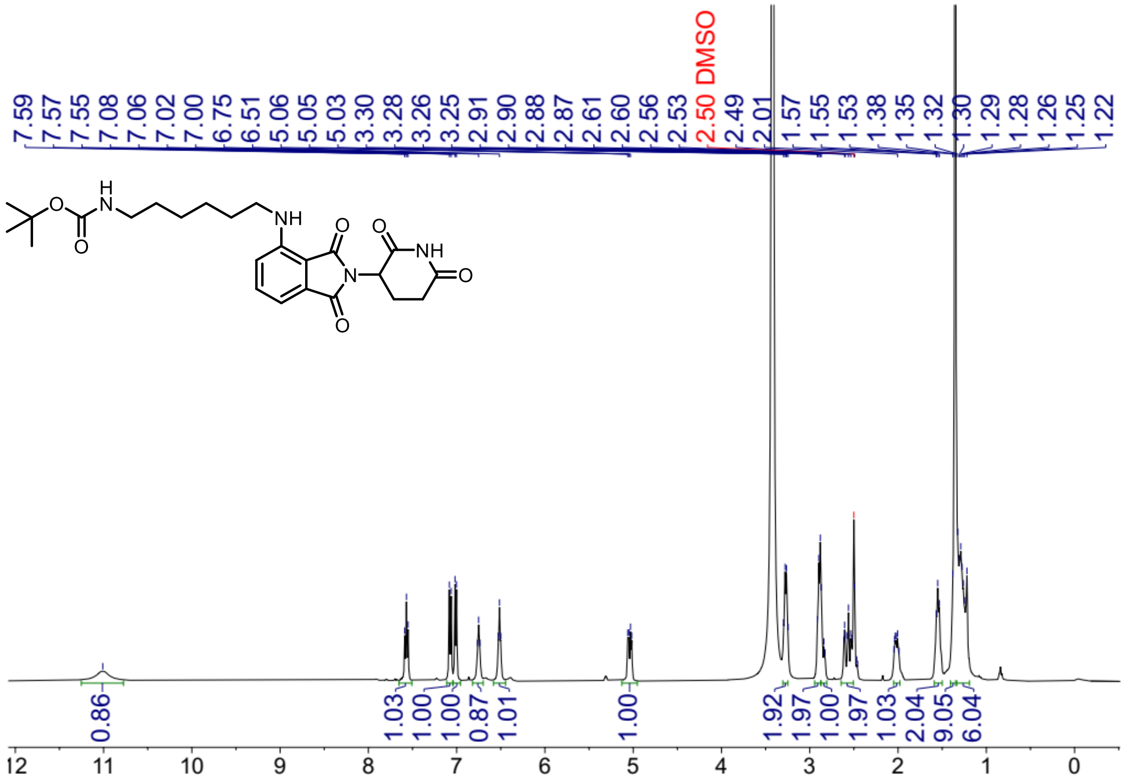

^{13}C NMR (101 MHz, DMSO-d_6) of compound 9j

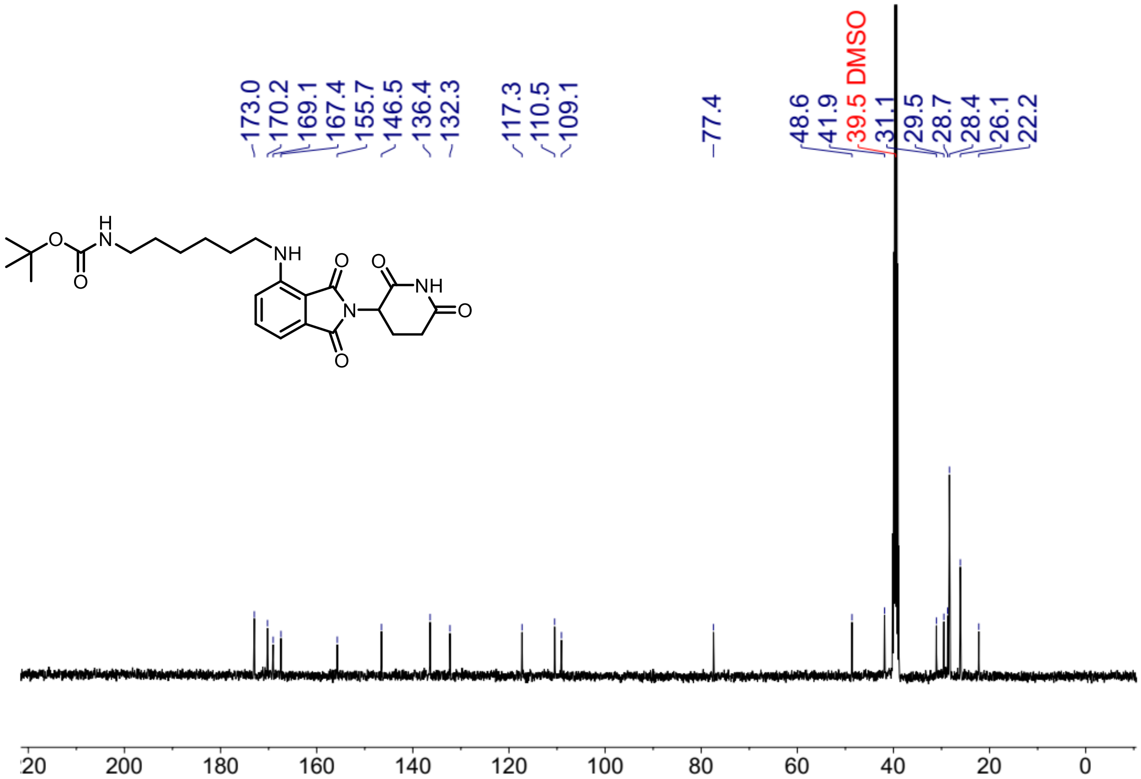

^{1}H NMR (400 MHz, DMSO-d_6) of t compound 9k

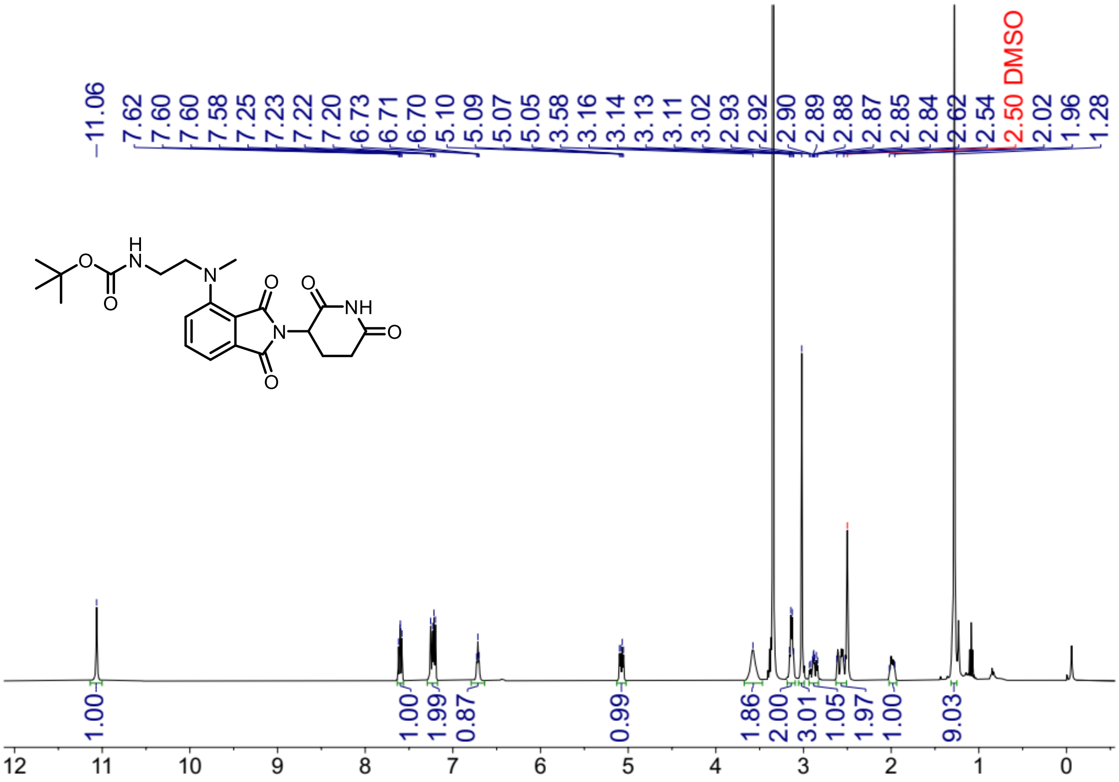

^{13}C NMR (101 MHz, DMSO-d_6) of compound 9k

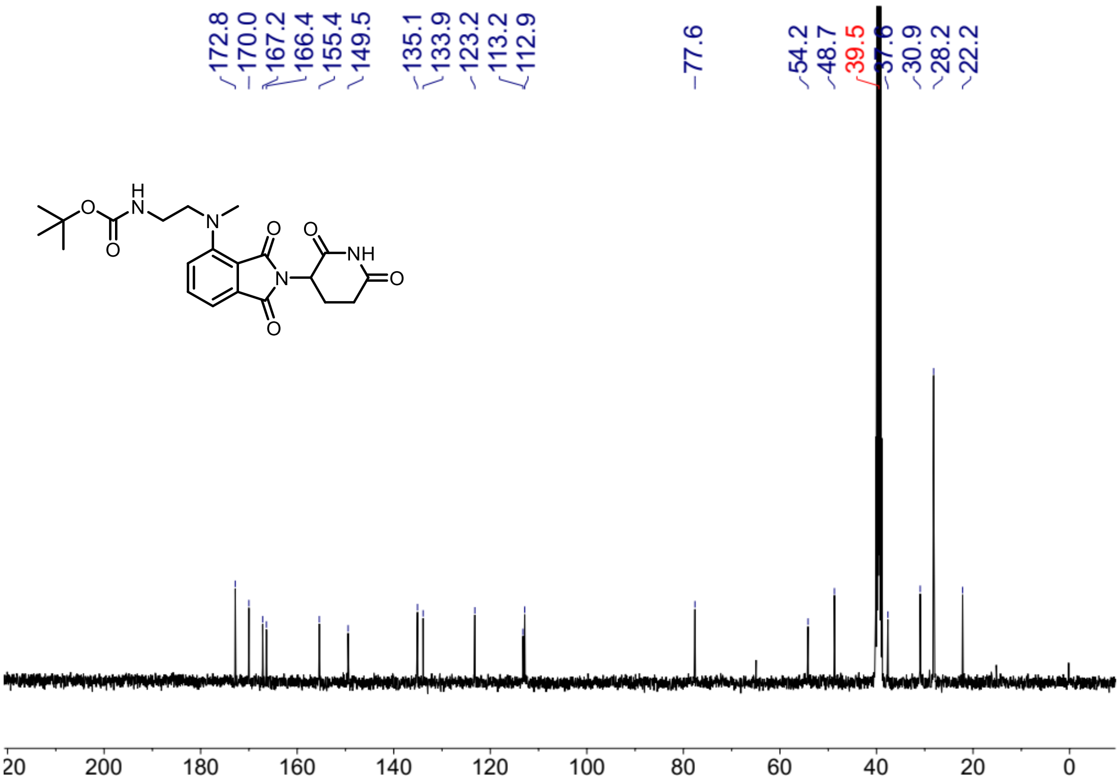

^{1}H NMR (400 MHz, DMSO-d_6) of compound 9l

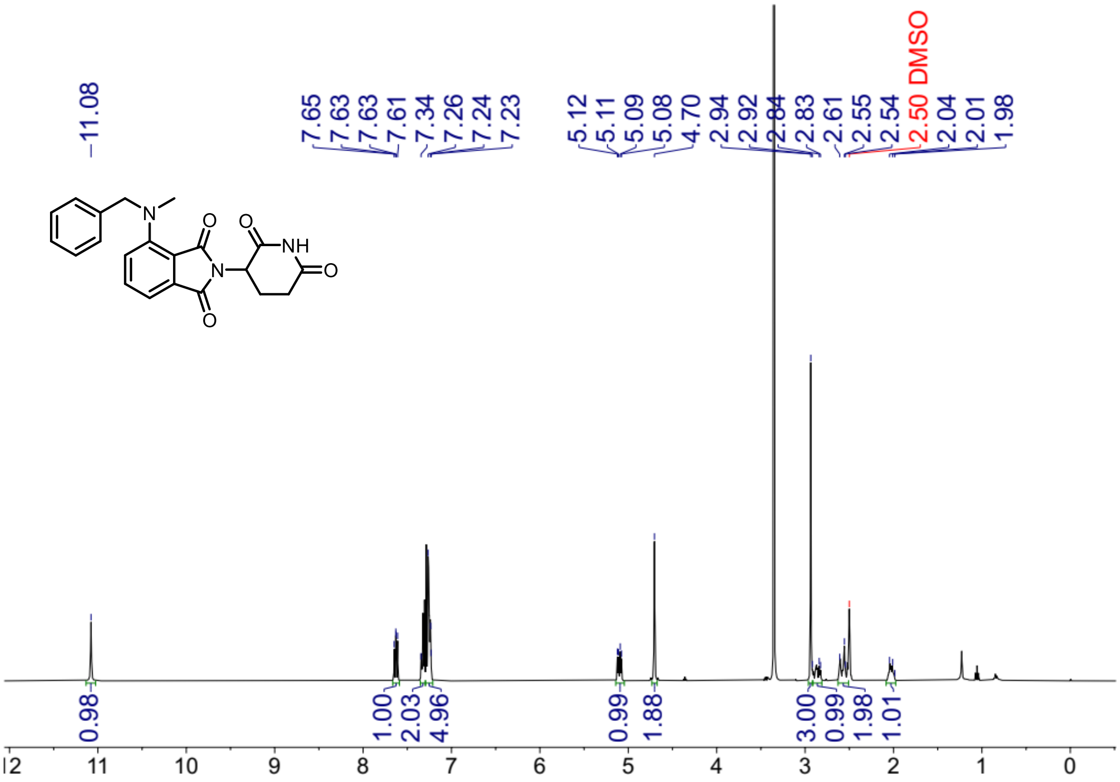

^{13}C NMR (101 MHz, DMSO-d_6) of compound 9l

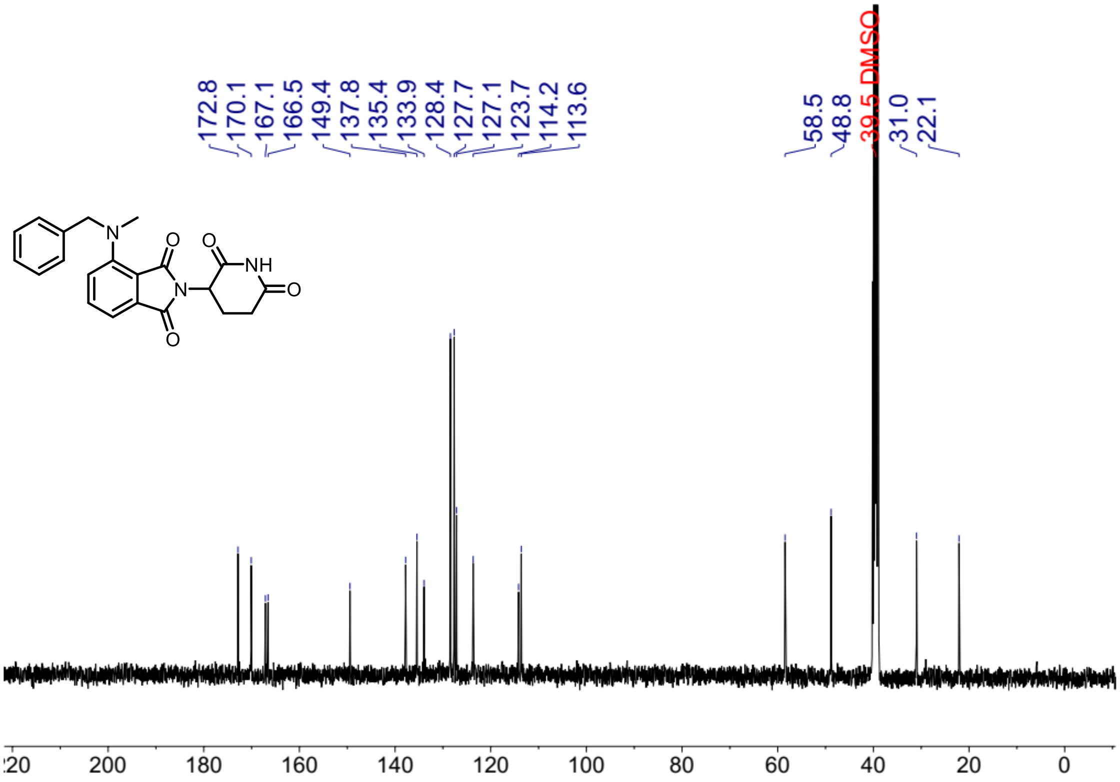

^{1}H NMR (400 MHz, DMSO-d_6) of compound 9m

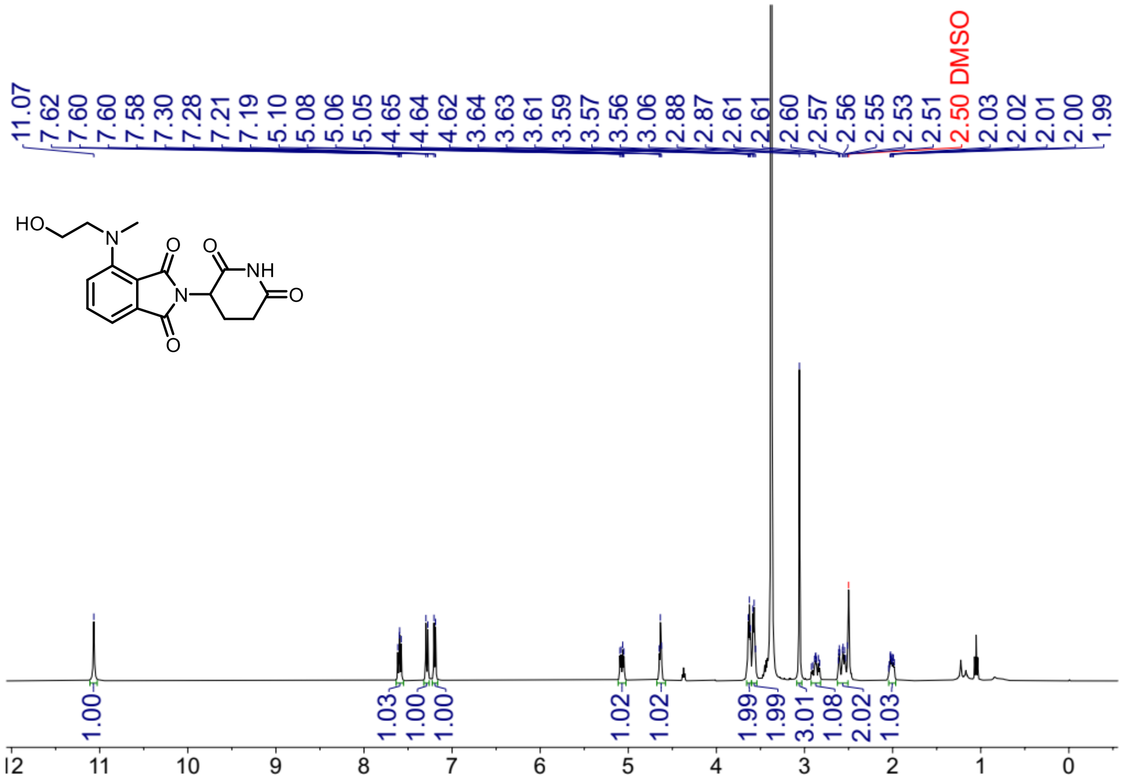

^{13}C NMR (101 MHz, DMSO-d_6) of compound 9m

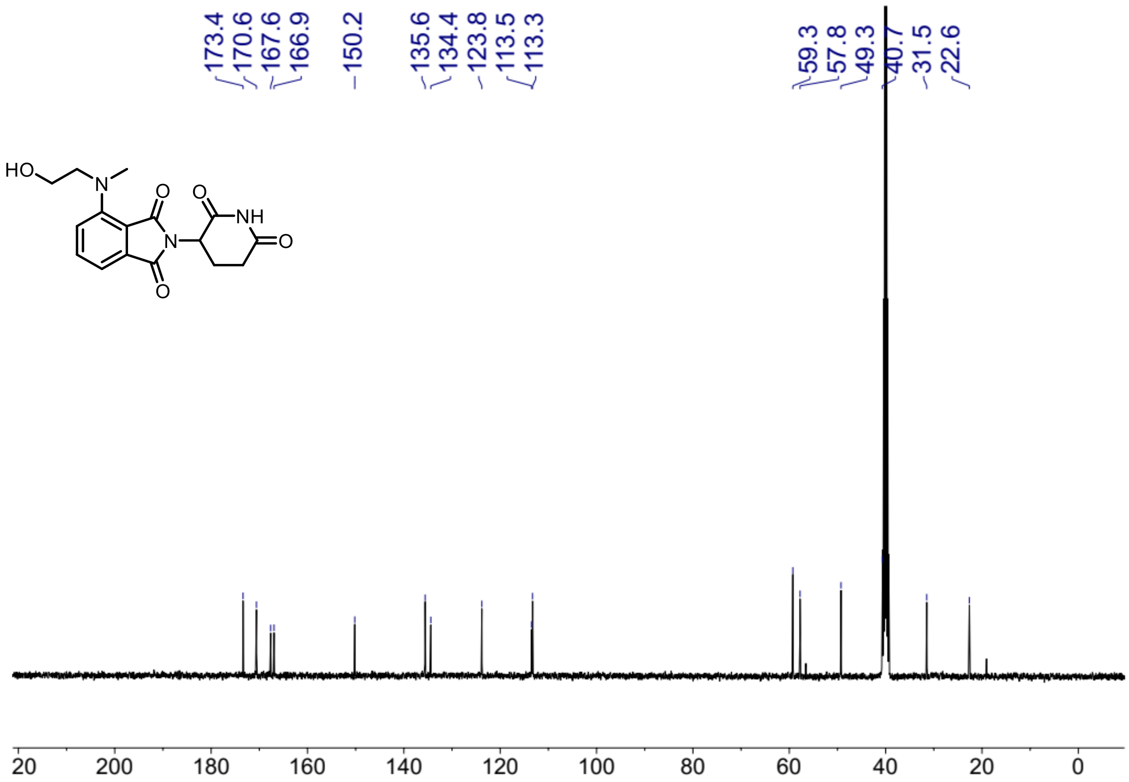

^{1}H NMR (400 MHz, DMSO-d_6) of compound 9n

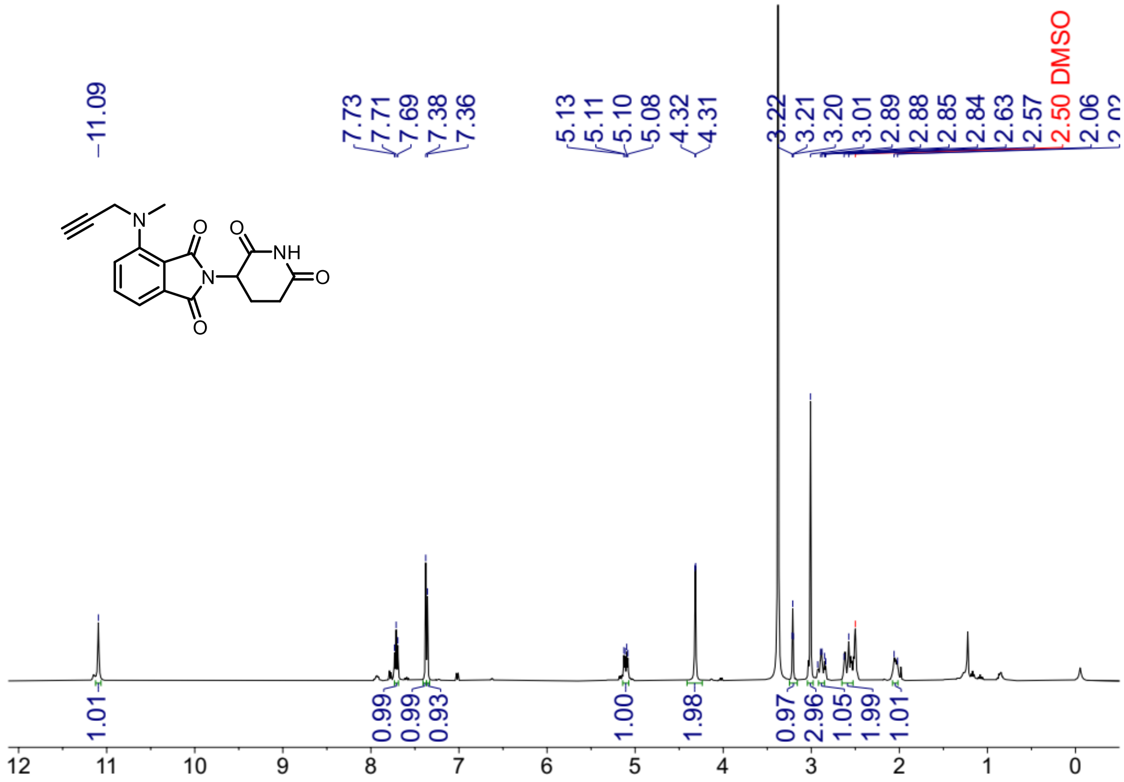

^{13}C NMR (101 MHz, DMSO-d_6) of compound 9n

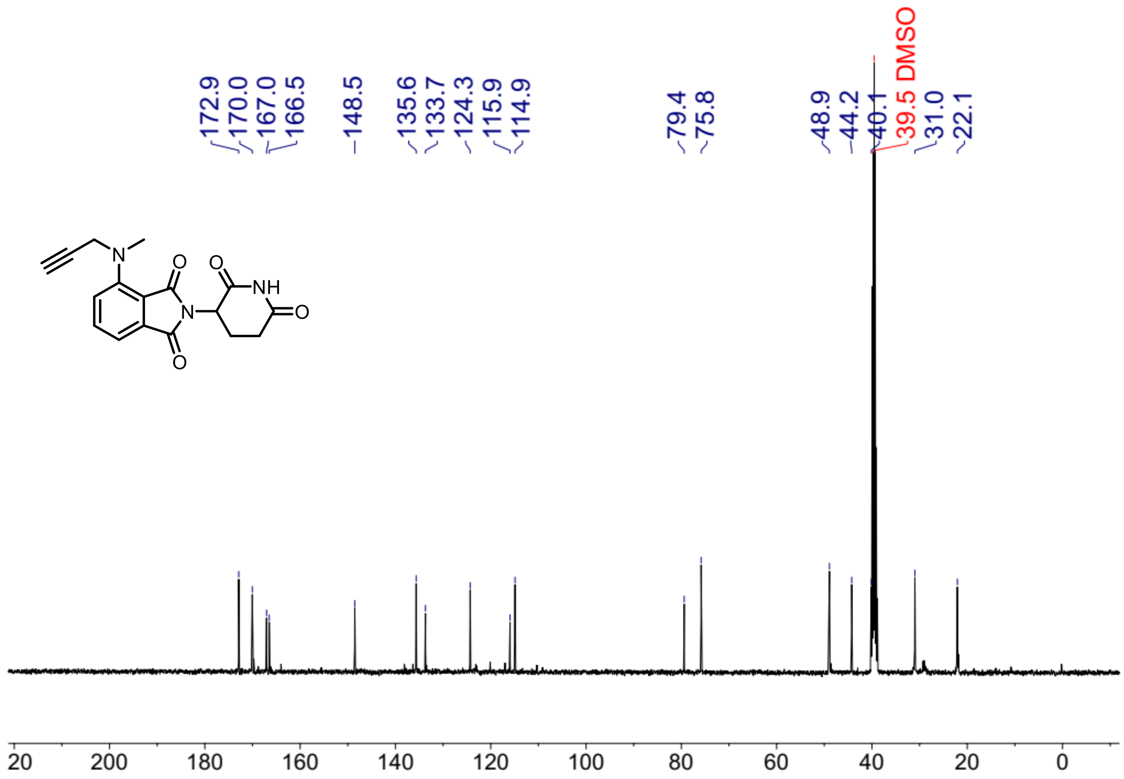

^{1}H NMR (400 MHz, DMSO-d_6) of compound 9o

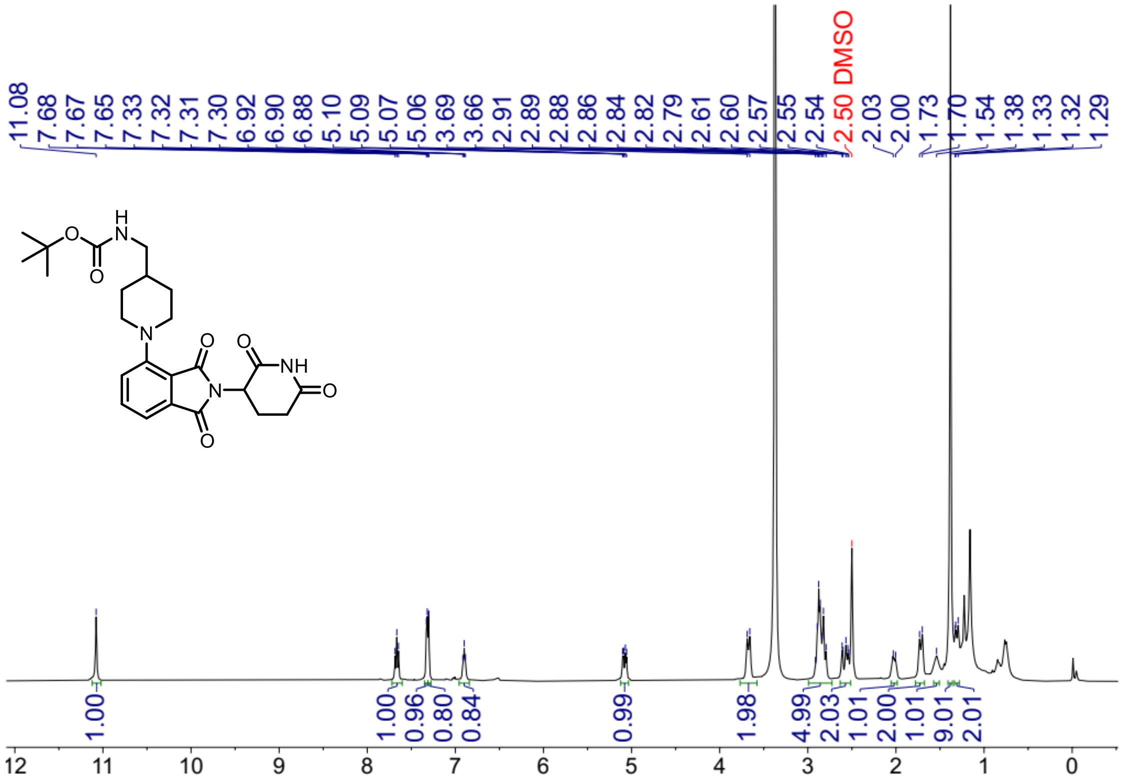

^{13}C NMR (101 MHz, DMSO-d_6) of compound 9o

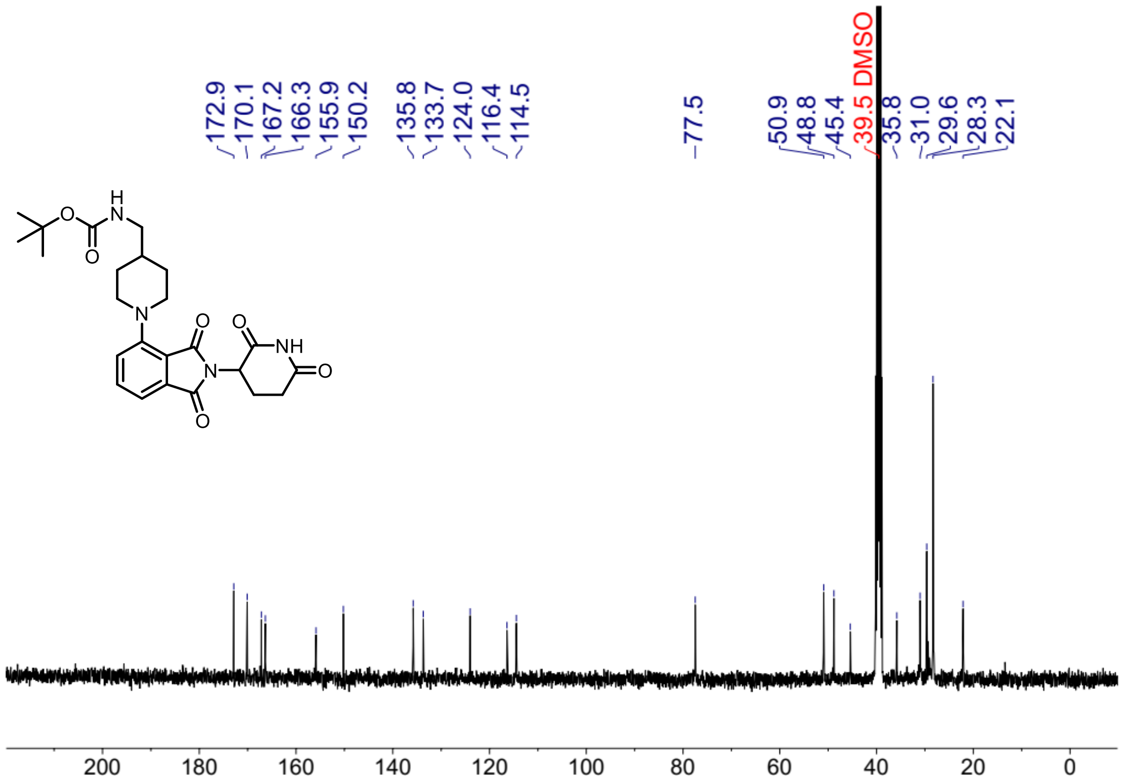

^{1}H NMR (400 MHz, DMSO-*d*$_6$) of compound 9p

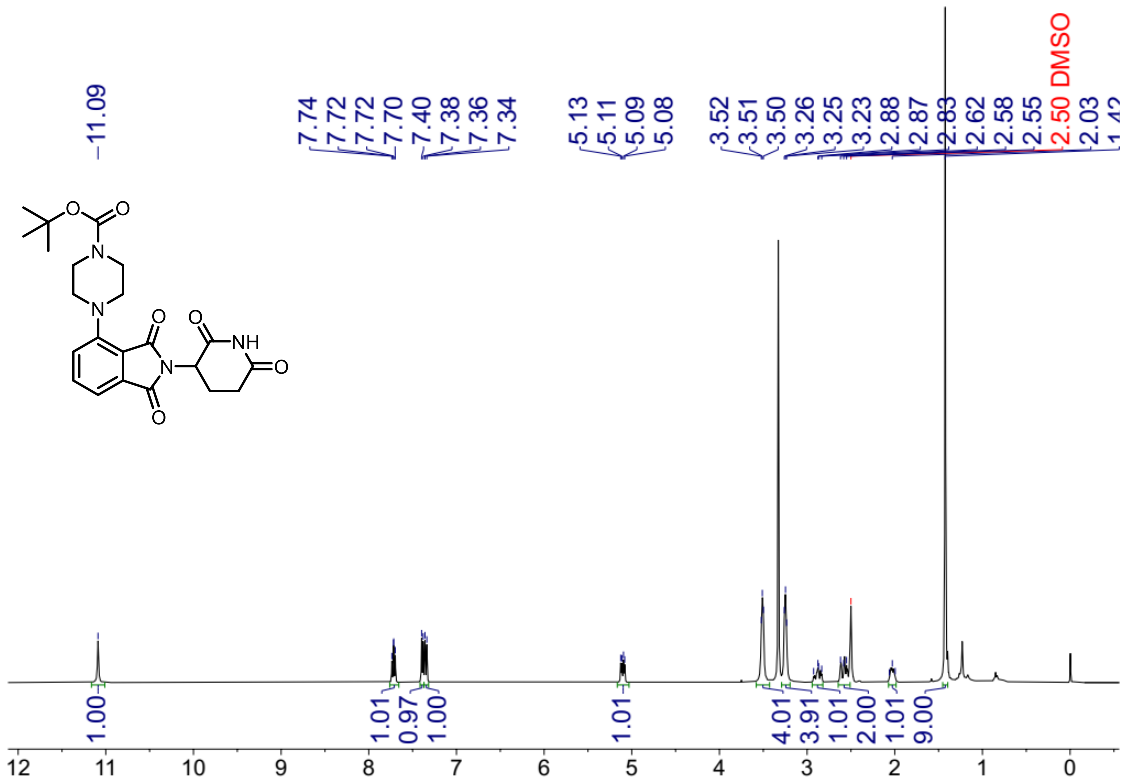

^{13}C NMR (101 MHz, DMSO-*d*$_6$) of compound 9p

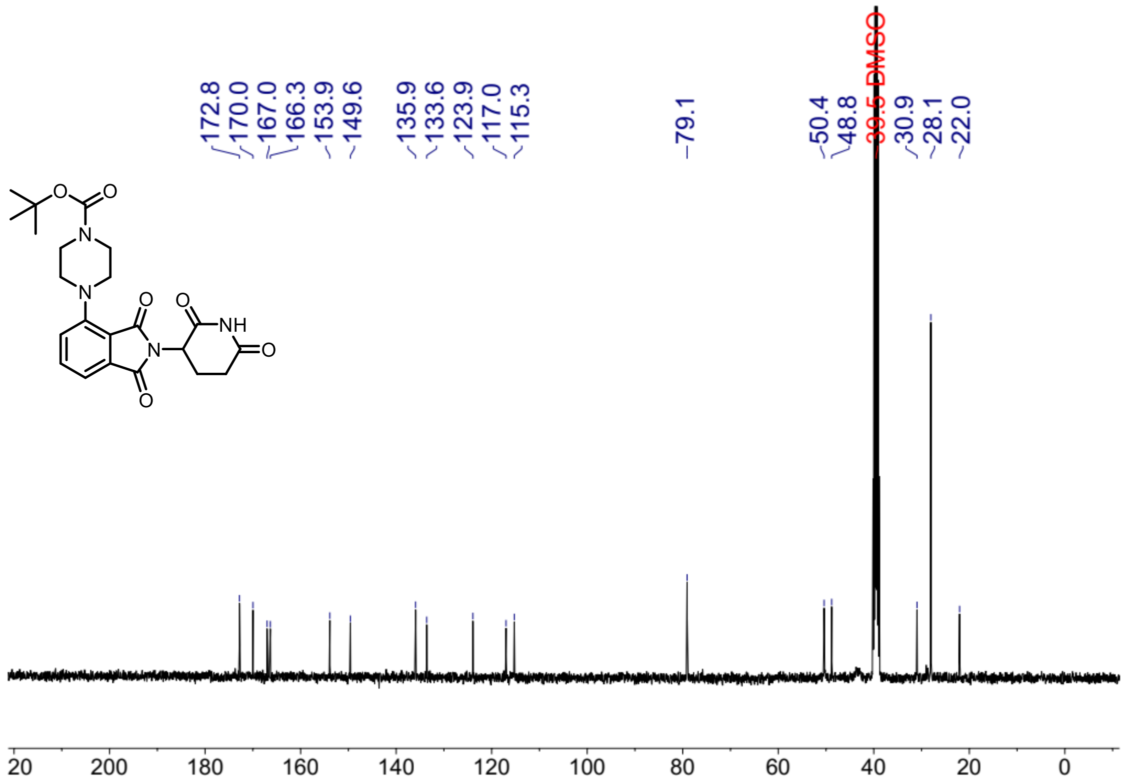

^{1}H NMR (400 MHz, DMSO-d_6) of compound 9q

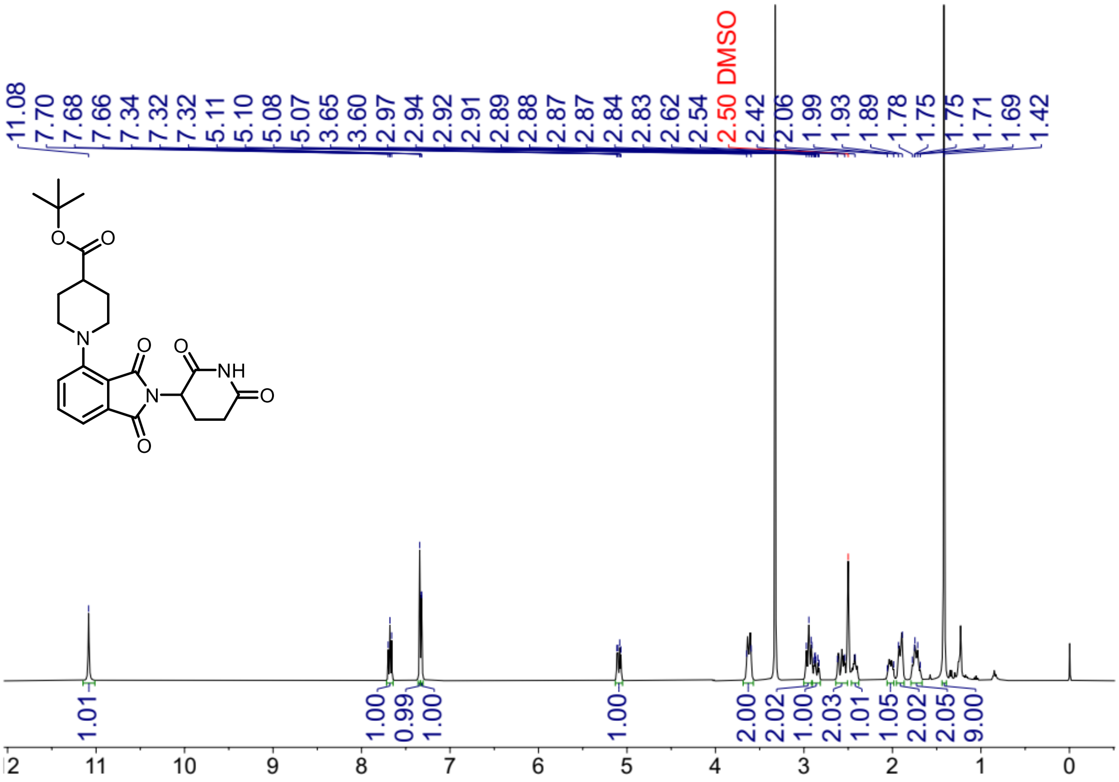

^{13}C NMR (101 MHz, DMSO-d_6) of compound 9q

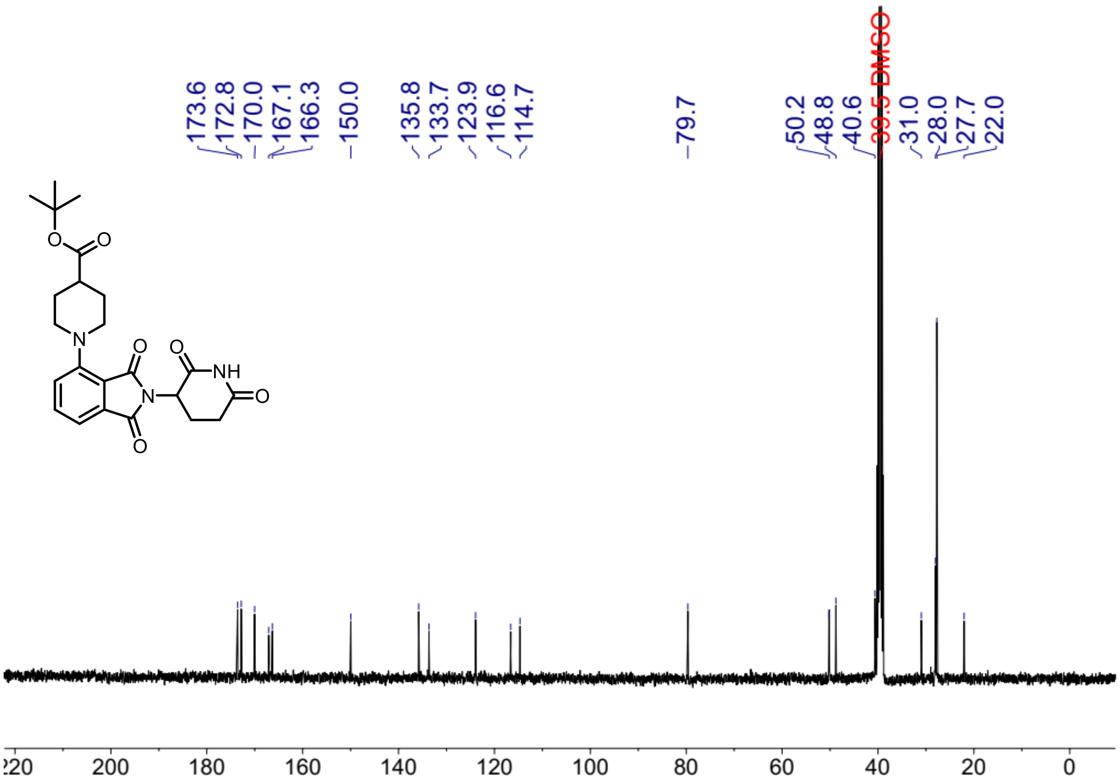

^{1}H NMR (400 MHz, DMSO-d_6) of compound 9r

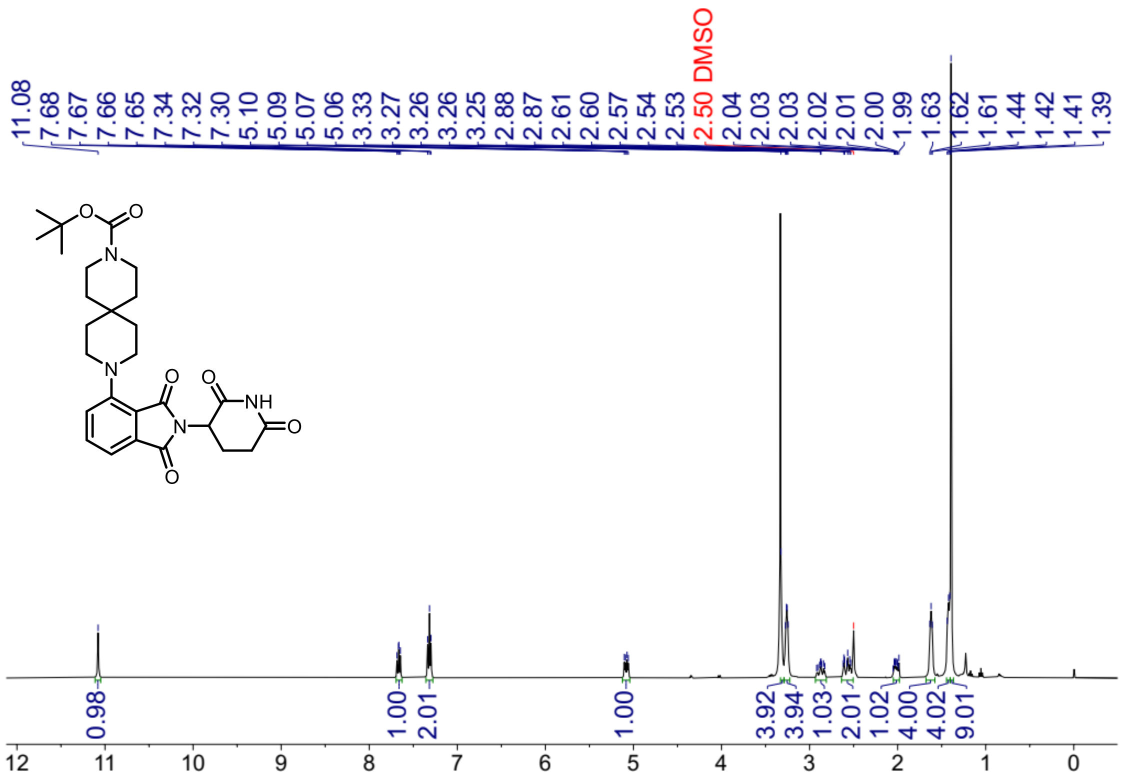

^{13}C NMR (101 MHz, DMSO-d_6) of compound 9r

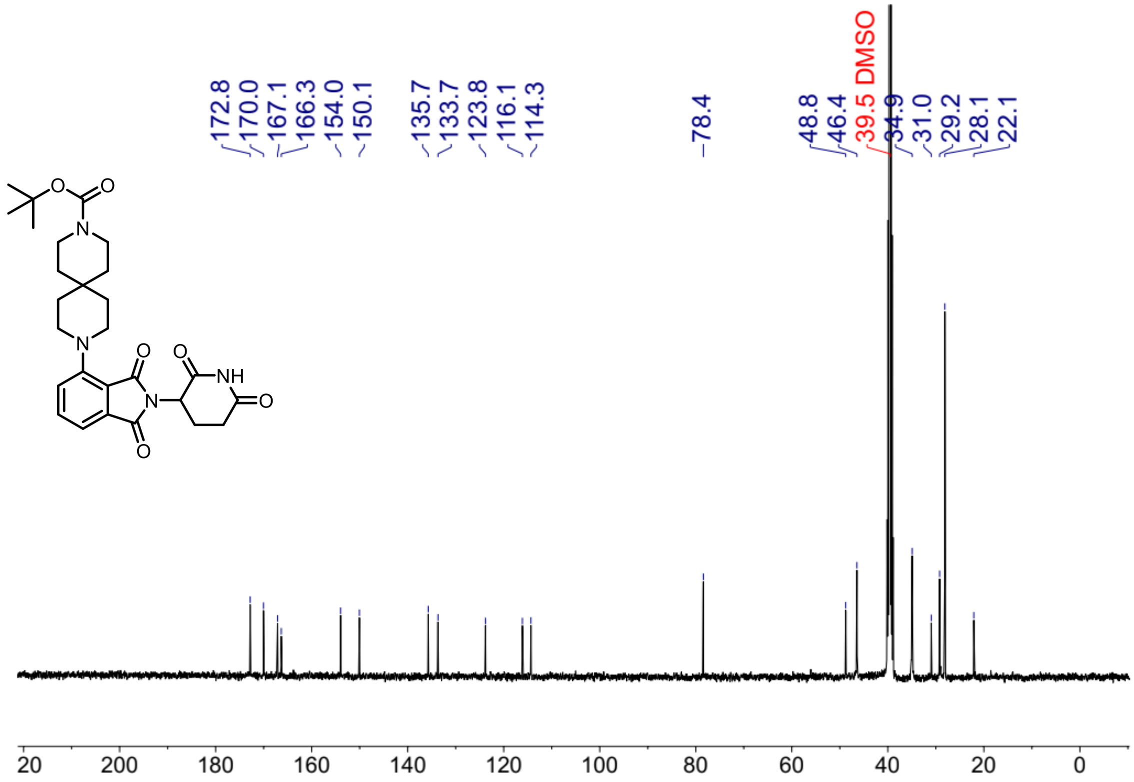

^{1}H NMR (400 MHz, DMSO-d_6) of compound 9s

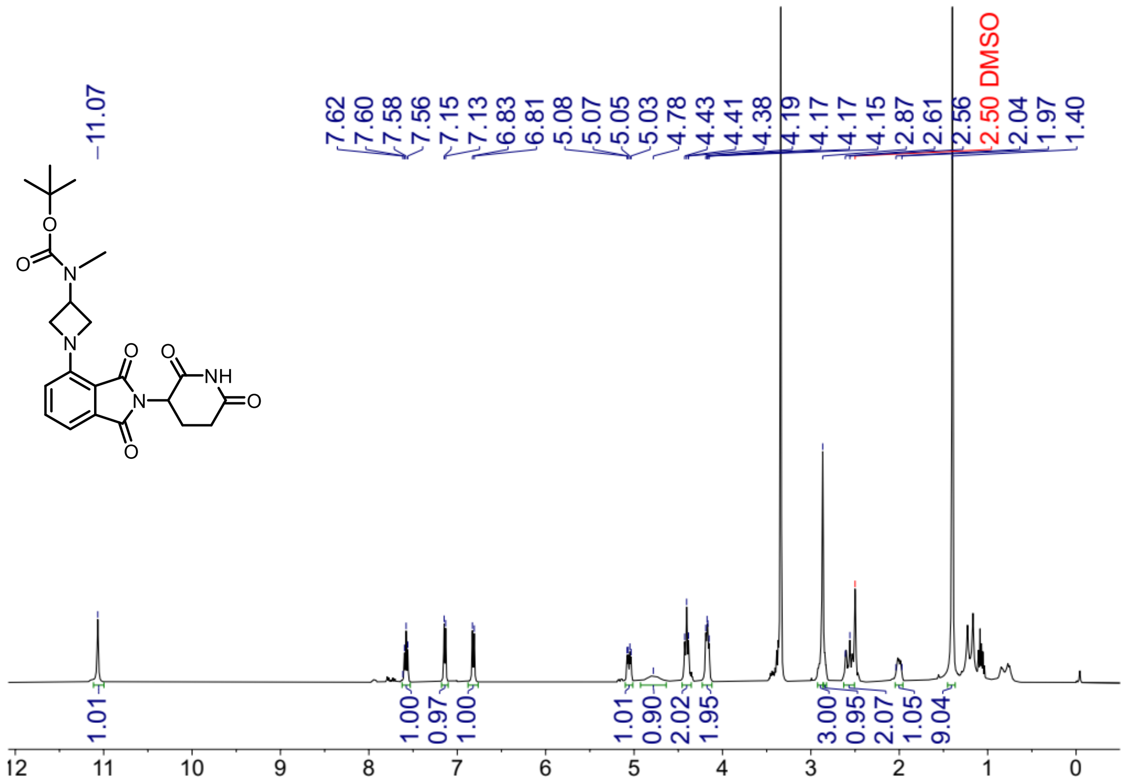

^{13}C NMR (101 MHz, DMSO-d_6) of compound 9s

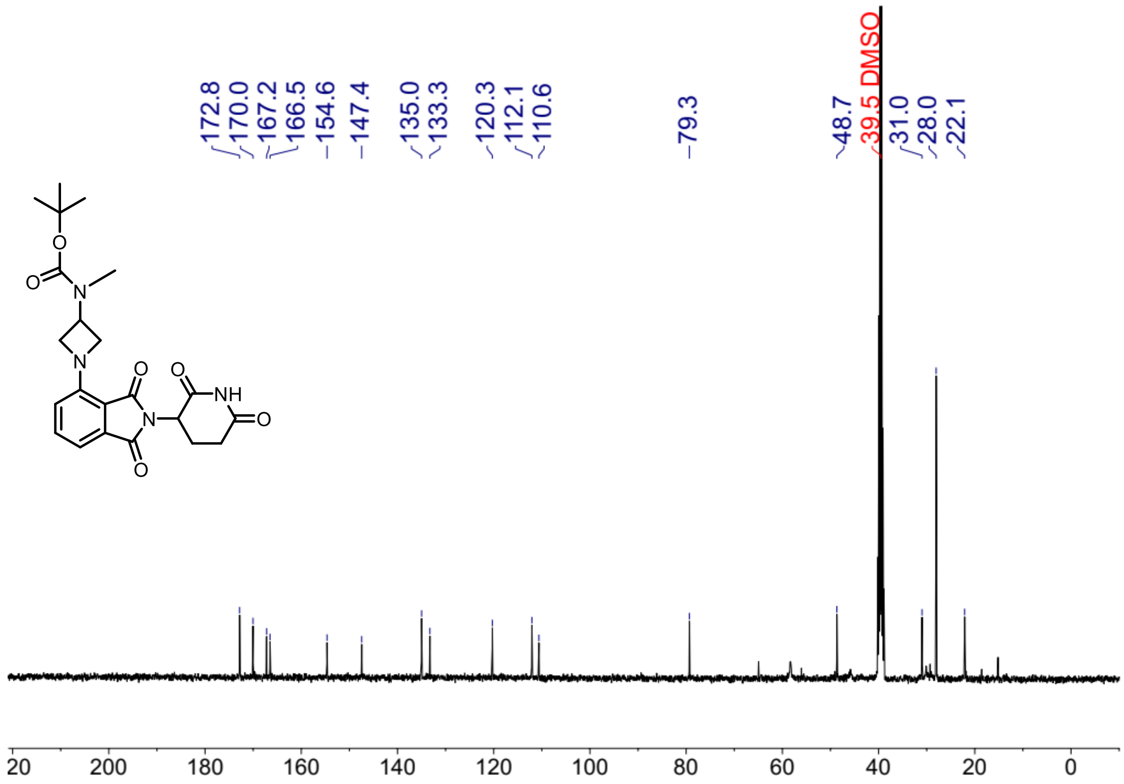

^{1}H NMR (400 MHz, DMSO-d_6) of compound 10a

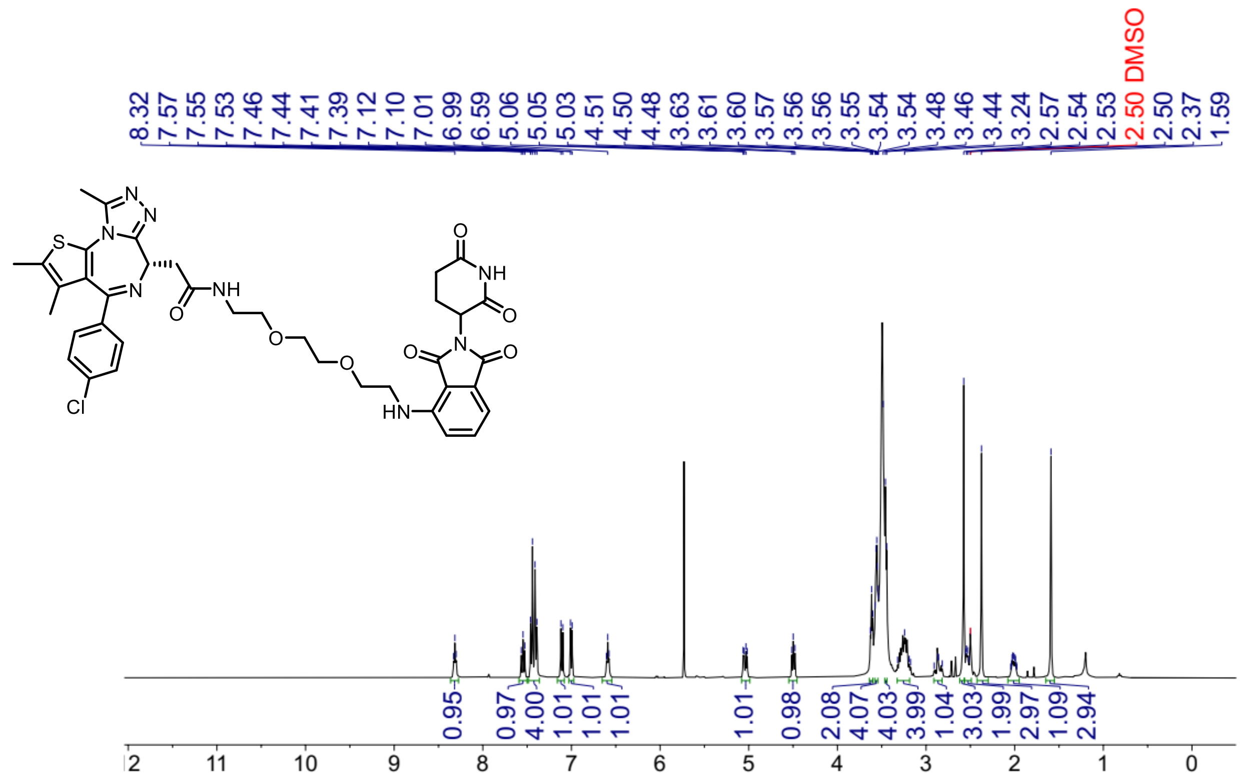

^{13}C NMR (101 MHz, DMSO-d_6) of compound 10a

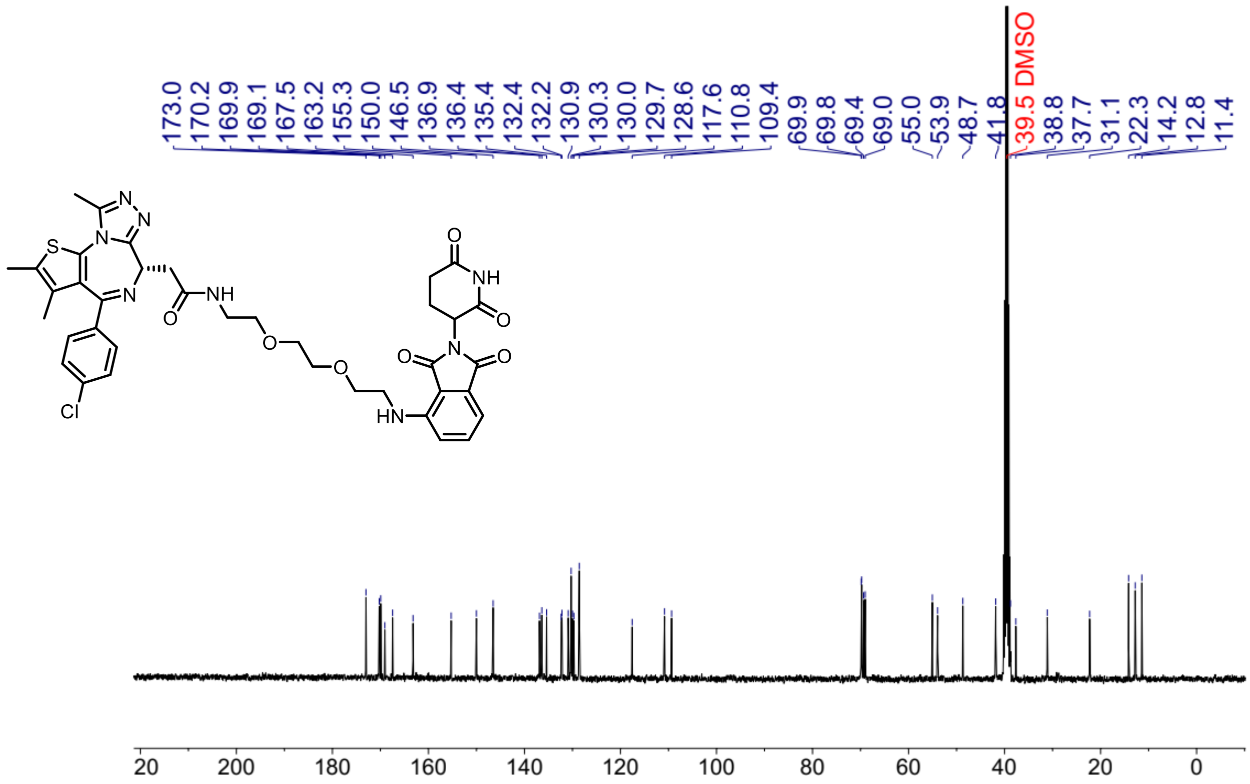

^{1}H NMR (400 MHz, DMSO-d_6) of compound 10b

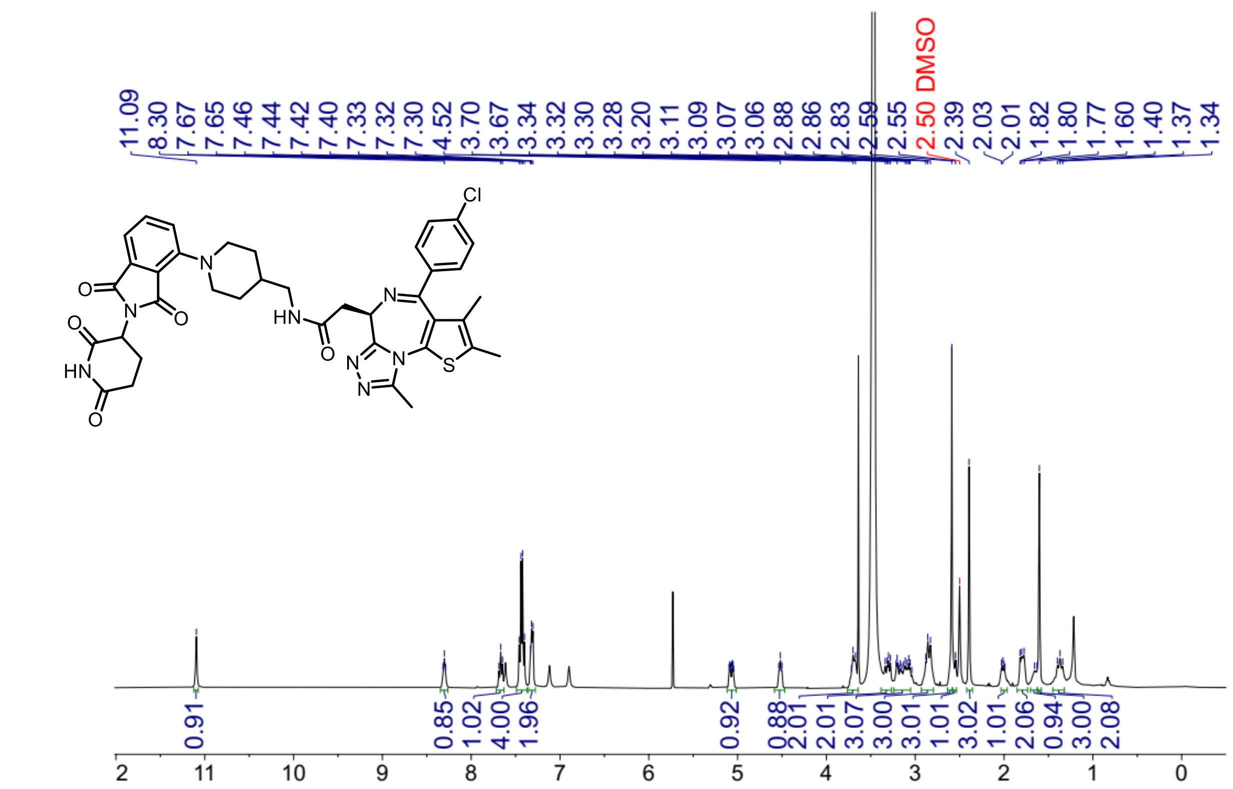

^{13}C NMR (101 MHz, DMSO-d_6) of compound 10b

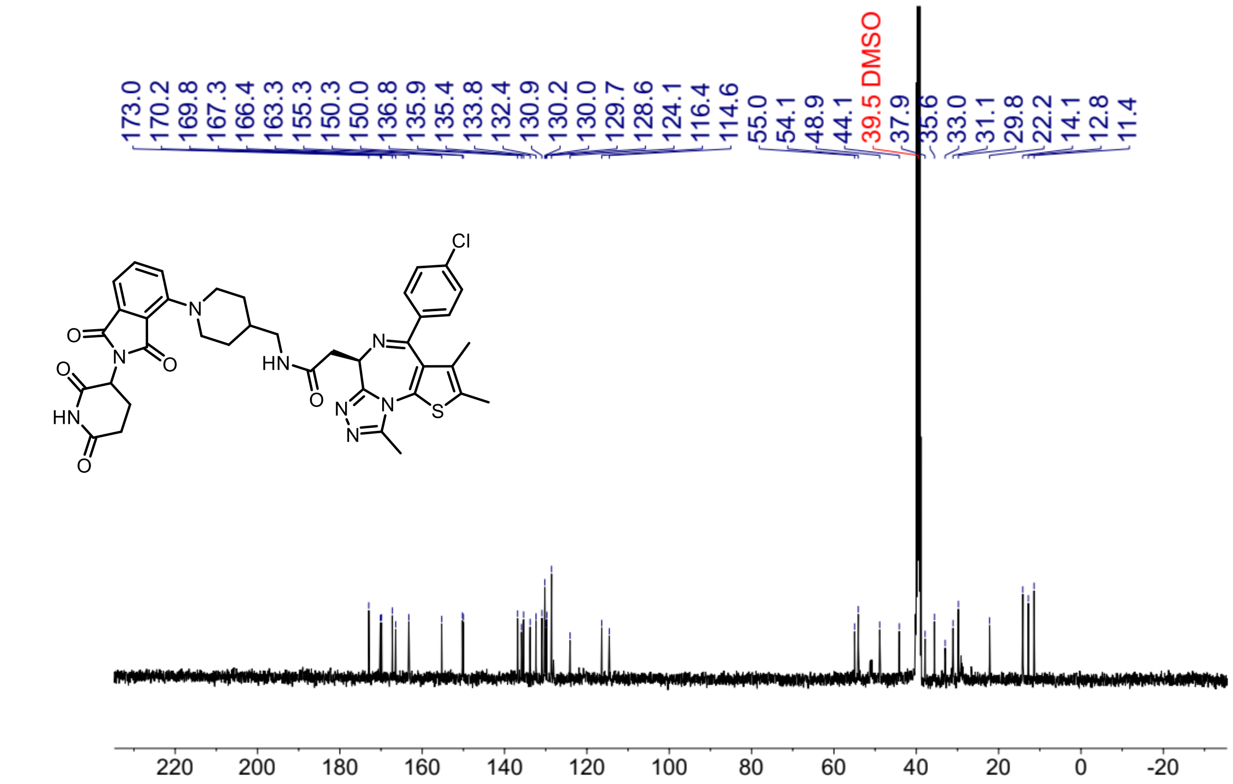

^{1}H NMR (400 MHz, DMSO-d_6) of compound 10c

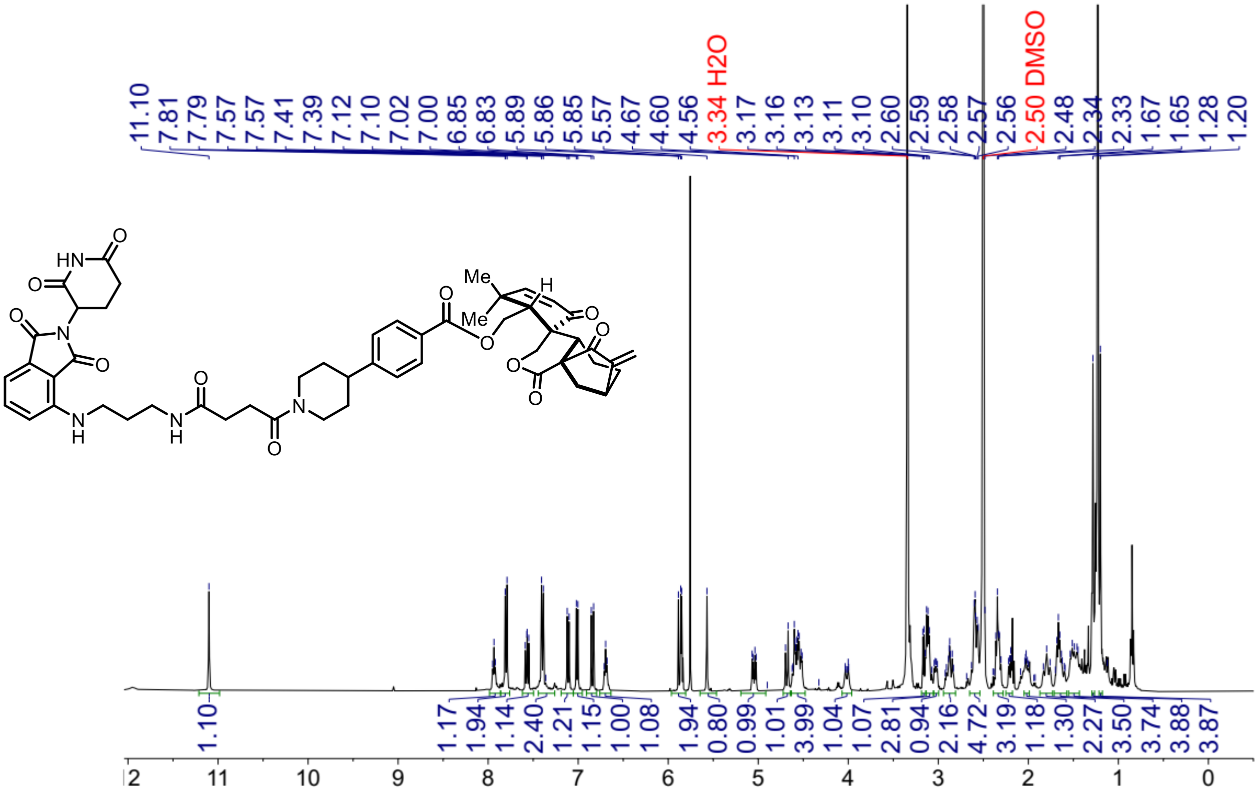

^{13}C NMR (101 MHz, DMSO-d_6) of compound 10c

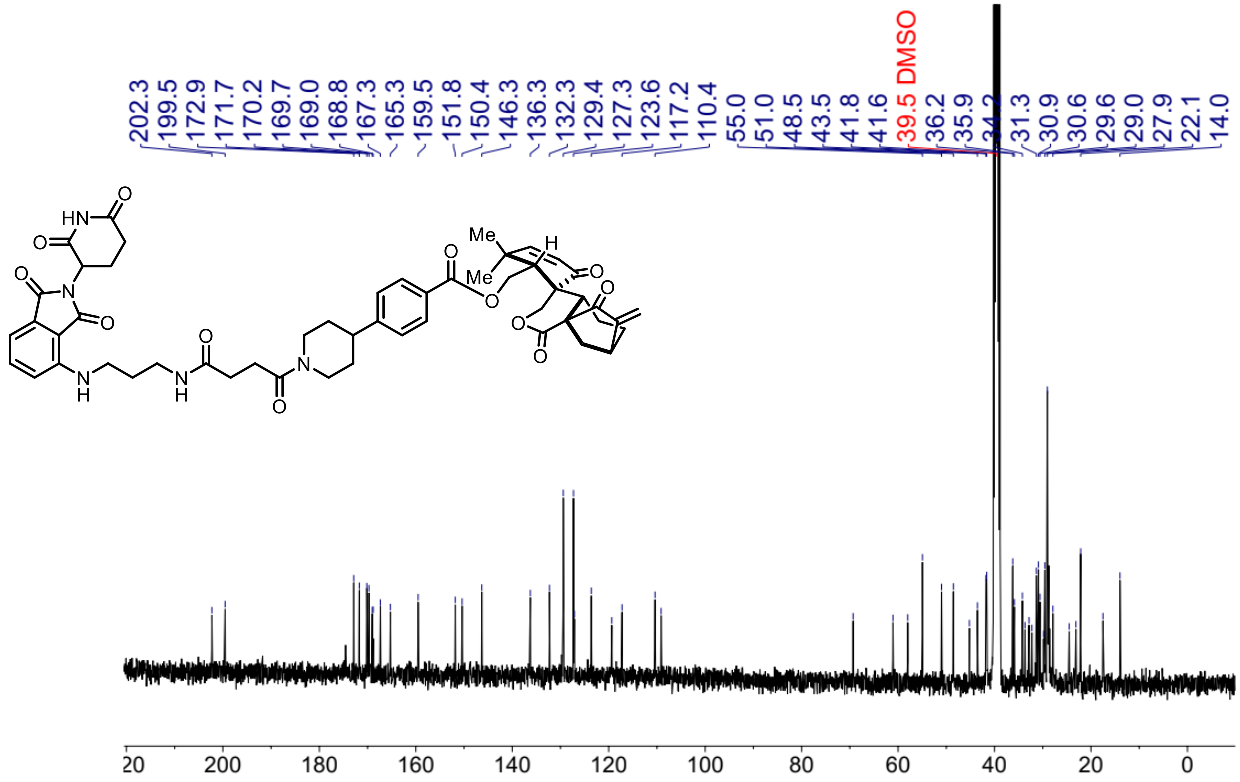

^{1}H NMR (400 MHz, DMSO-d_6) of compound 10d

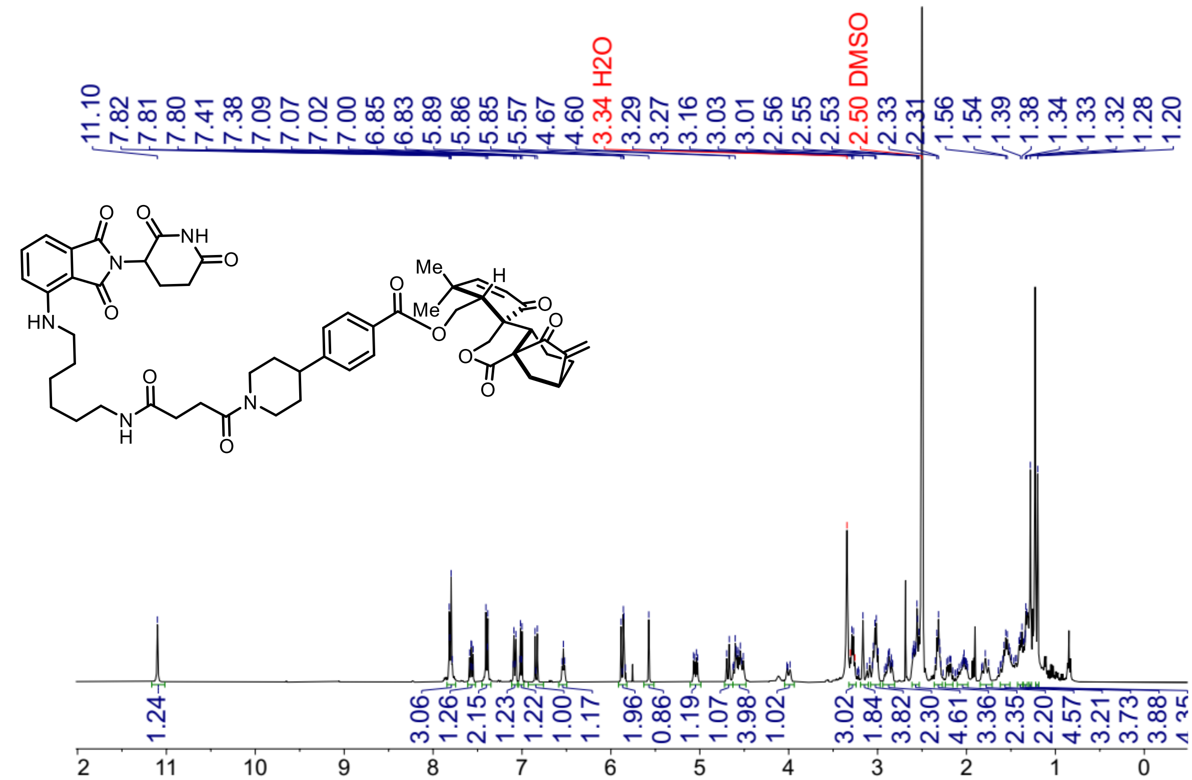

^{13}C NMR (101 MHz, DMSO-d_6) of compound 10d

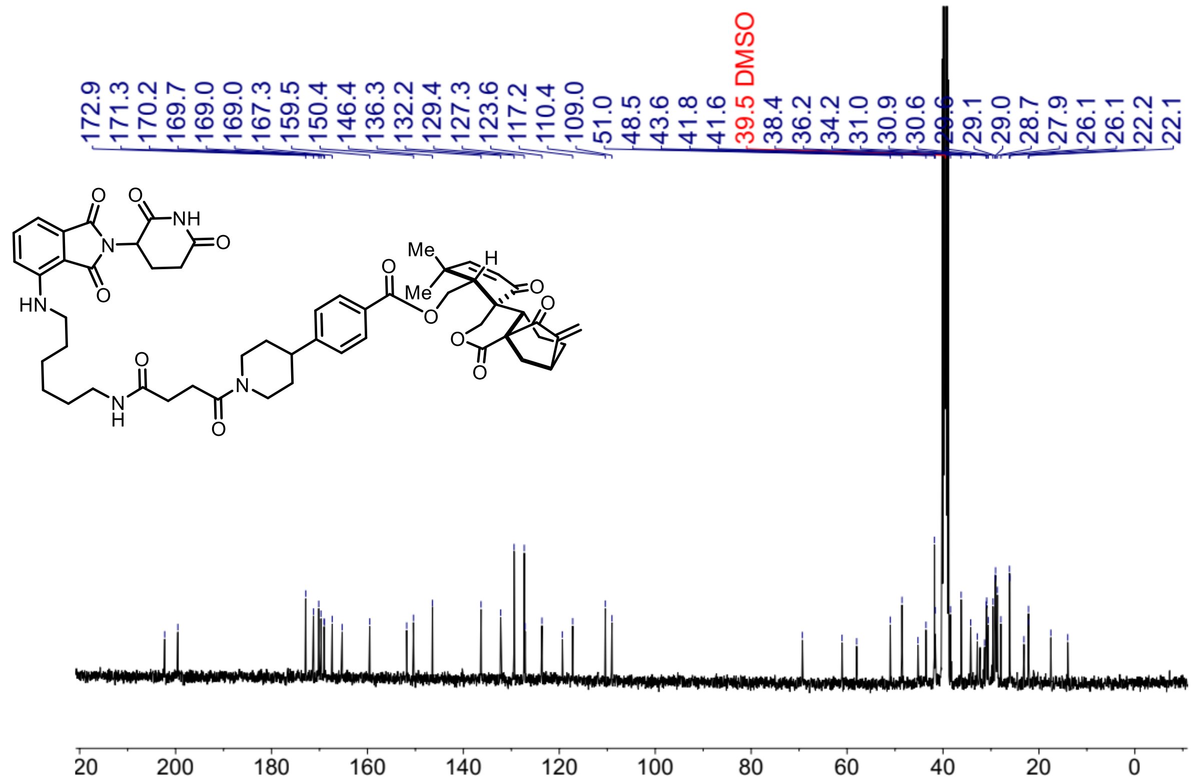

Supplement: Supplementary file 1 — Supporting information [file 42004_2026_1956_MOESM1_ESM.pdf]
